# Computational analysis reveals historical trajectory of East Polynesian lunar calendars

**DOI:** 10.1371/journal.pone.0353287

**Published:** 2026-07-29

**Authors:** Miguel Valério, Fabio Tamburini, Michele Corazza

**Affiliations:** 1 Department of Prehistory, Archaeology, Ancient History, Medieval History and Historiographic Sciences and Techniques, University of Murcia, Murcia, Spain; 2 FICLIT, University of Bologna, Bologna, Italy; 3 CIRSFID-ALMA AI, University of Bologna, Bologna, Italy; University of Passau: Universitat Passau, GERMANY

## Abstract

We investigate a type of lunar calendar known as lists of the ‘nights of the moon’, found throughout East Polynesia, including Rapa Nui (Easter Island). Using computational methods, we analyzed the lexical and structural divergence of 48 calendrical lists from all major archipelagos, each containing about 30 night names. Our results, presented as a rooted phylogenetic tree, show a split into two main groups: one including lists from Rapa Nui, Mangareva, and the Marquesas; the other comprising lists from New Zealand, Hawai’i, the Cook Islands, the Austral Islands, Tahiti, and the Tuamotu. This result aligns with a recent alternative classification of East Polynesian languages into ‘Distal’ (Marquesan, Mangarevan, Rapanui) and ’Proximal’ (Māori, Hawaiian, Tahitian, etc.) subgroups. Since both language and lunar calendars are symbolic systems passed down and changed within communities—and given the geographic isolation of many archipelagos—we interpret this correspondence as evidence that the early divergence of East Polynesian lunar calendars mirrors early population movements and language splits in the region.

## Introduction: The ‘nights of the moon’ in East Polynesia

Lunar calendars based on synodic months (complete cycles of the Moon’s phases) were once widespread in Polynesia, with the year divided into 12 or 13 lunations [1]. Each lunar month followed the astronomical 29.53-day lunar cycle, which includes the phases of the new moon, the first quarter, the full moon, the last quarter, and then cycles back to the new moon. Nights, rather than days, served as the reference point. In East Polynesia, each ‘night of the moon’ had a specific name, a tradition that extended across all islands where East Polynesian languages are still spoken today (Table 1). This *naming* practice contrasts with the calendars of West Polynesia and additional areas where other Polynesian languages are spoken, such Samoa, Tonga, and Tokelau. There, lunar calendars typically consisted of lists where nights were mostly *numbered* rather than named. Because the *numbering* calendars from West Polynesia and elsewhere included a few names shared with East Polynesia (see below), their type may have been the source of the *naming* system.

**Table 1:** Examples of East Polynesian lists of ‘nights of the moon’ from Rapa Nui/Easter Island, Hawai’i, Aotearoa/New Zealand, and Tahiti.

	Rapa Nui/Easter Island	Hawai’i	Aotearoa/New Zealand	Tahiti
	(RPN2)	(HAW1b)	(MAO3)	(TAH3)
1	Tireo	Hilo	Whiro	Tireo
2	Hiro	Hoaka	Tirea	Hiro-hiti
3	Oata	Kū Kahi	Hoata	Hoata
4	(O) Ari	Kū Lua	Oue	Hamiama-mua
5	Kokore tahi	Kū Kolu	Okoro	Hamiama-roto
6	Kokore rua	Kū Pau	Tamatea-tutahi	Hamiama-muri
7	Kokore toru	’Ole Kū Kahi	Tamatea-turua	Tamatea-mua
8	Kokore ha	’Ole Kū Lua	Tamatea-tutoru	Tamatea-roto
9	Kokore rima	’Ole Kū Kolu	Tamatea-tuwha	Tamatea-muri
10	Kokore ono	’Ole Kū Pau	Huna	Huna
11	Maharu	Huna	Ari	’Ari
12	(O) Hua	Mōhalu	Maure	Maharu
13	Atua	Hua	Mawharu	Hua
14	(O) Hotu	Akua	Ohua	Maitu
15	Maure	Hoku (Full Moon)	Atua	Hotu (Full Moon)
16	Ina-ira	Mālani	Oturu (Full Moon)	Mara’i
17	Rakau	Kulu	Rakau-nui	Turu
18	Motohi (Full Moon)	Lā’au Kū Kahi	Rakau-matohi	Rā’āu-mua
19	Kokore tahi	Lā’au Kū Lua	Takirau	Rā’āu-roto
20	Kokore rua	Lā’au Pau	Oike	Rā’āu-muri
21	Kokore toru	’Ole Kū Kahi	Korekore-tutahi	’Ore’ore-mua
22	Kokore ha	’Ole Kū Lua	Korekore-turua	’Ore’ore-roto
23	Kokore rima	’Ole Kū Pau	Korekore-piri-ki-nga-Tangaroa	’Ore’ore-muri
24	Tapume	Kanaloa Kū Kahi	Tangaroa-roto	Ta’aroa-mua
25	Matua	Kanaloa Kū Lua	Tangaroa-kiokio	Ta’aroa-roto
26	(O) Rongo	Kanaloa Pau	Tangaroa-whakapau	Ta’aroa-muri
27	(O) Rongo Tane	Kāne	Otane	Tāne
28	Mauri-nui	Lono	Orongonui	Ro’o-nui
29	Mauri-kero	Mauli	Mauri	Ro’o-mauri
30	(O) Mutu	Muku	Mutuwhenua / Omutu	Mutu

**Fig 1:**
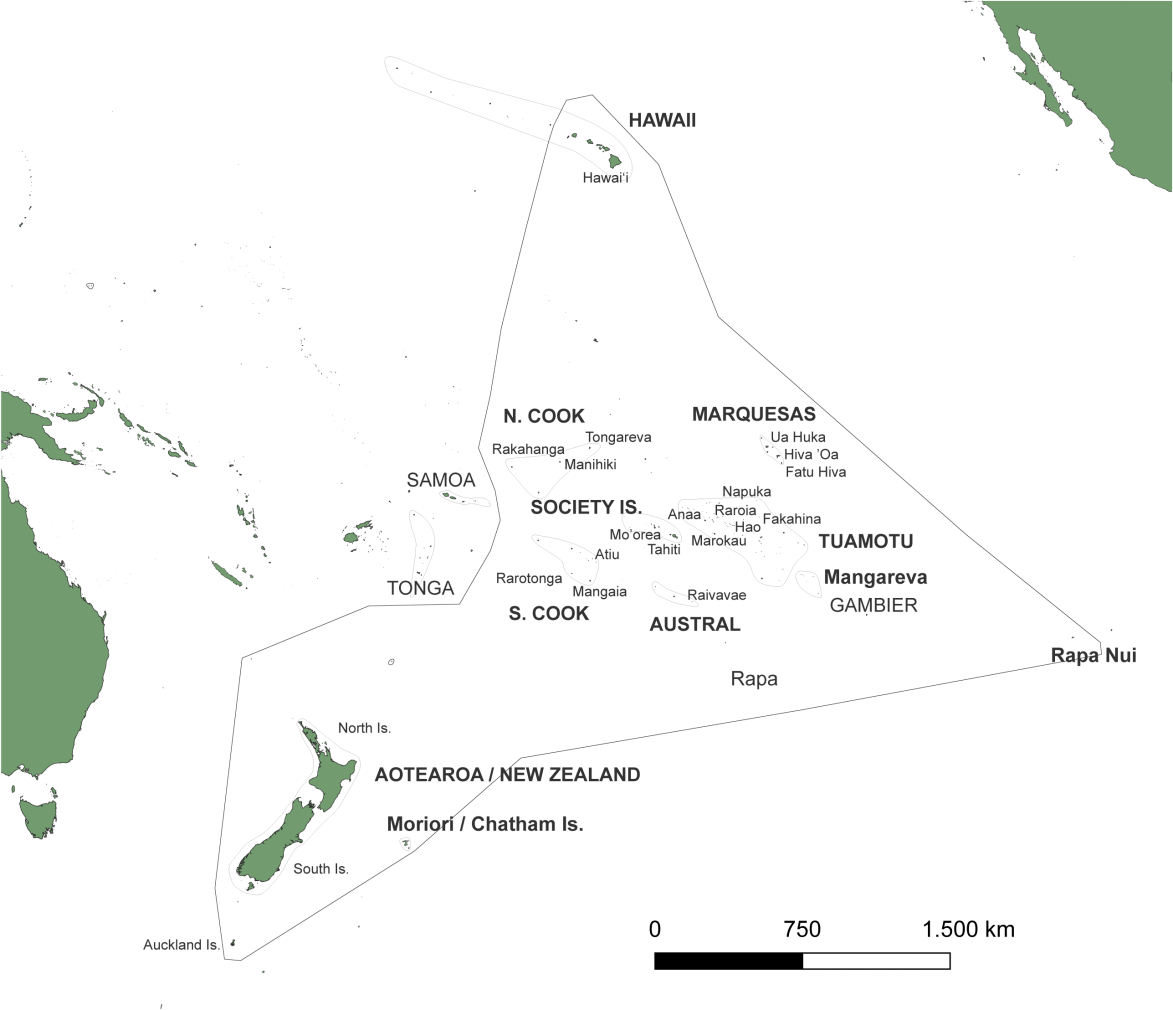
The ‘triangle’ of East Polynesian islands and the Samoan-Tongan area (West Polynesia). Made with Natural Earth. Free vector and raster map data @ naturalearthdata.com.

East Polynesia is a large oceanic region with shared linguistic and social traits, and the last to be settled by humans. It includes the islands of Aotearoa/New Zealand, Moriori/Chatham, Society (with Tahiti), northern and southern Cook, Austral, Gambier (with Mangareva), Tuamotu, Marquesas, Rapa Nui/Easter Island and Hawai’i, among others (Fig 1). The sequence of its settlement, whose point of origin is believed to be West Polynesia (the Samoa-Tonga area), is still debated based on archaeological, anthropological, linguistic, and genetic evidence [2–4]. Linguistically, East Polynesia is home to a subgroup of Polynesian tongues [5,6], spoken across islands in considerable geographical isolation. Since the typological difference between naming and numbering calendrical types separates the East-Polynesian-speaking archipelagos from West Polynesia [7,8], the prototype for the ‘naming’ lists may have originated in a community of Proto-East Polynesian speakers.

The lunar calendar was tied to precolonial ways of life, marking suitable times for food planting and, above all, fishing. It was transmitted orally, though there is evidence that on Rapa Nui it was also inscribed on a wooden tablet in the still undeciphered Rongorongo script [9–11]. After the introduction of the European solar (Gregorian) calendar and Christian festivities, this way of measuring time largely ceased to function in most places. Knowledge of it has survived because some native authorities and outsiders wrote down “lists of the nights of the moon” in the 19th and first half of the 20th century. In the early 1900s, a Tahitian royal person described one list as a *parau* (‘record’) for “fishermen, recounting the nights when the fish run, this kind and that kind, (...) and the days which are favourable for planting food-plants” [12]. Reports from various locations coincide in that some nights were ‘glossed’ orally with indications of which fishing methods (if any) were appropriate for specific moon phases, such as torchlight night fishing [12,13].

Names or parts of names for nights that repeat in all or nearly all East Polynesian locations (cited here in their reconstructed Proto-East Polynesian forms) were likely part of the proto-calendar. Some (e.g.,*Tu(ʔ)u, *Firo, *Tamatea, *Taŋaroa, *Taane, *Roŋo, *Mauri) match the names of deities or supernaturals [14,15]. Other forms seem to describe phases of the moon: *Maseʔa ‘faintly perceptible’, *(ʔ)Ari ‘clearly visible’, *Funa ‘hidden’, *Fua ‘blossom’, *Fotu ‘appear, rise’, *Matofi ‘split in two’, *mate ‘die’, *Mutu* ‘end’ [15] (see S1 Appendix). Some nights formed series with the same main name (*Maseʔa *Same(ʔ)a, *Korekore, *Taŋaroa, etc.), followed by a word that specified the position of each homonymous night in the series. For this purpose, different systems were used, the main ones being: *tahi, *rua, *toru… ‘one, two, three...’; *muʔa, *roto, *muri ‘before, inside, after’; and *tahi, *roto, *fakaʔoti ‘one, inside, final(izing)’. The numbering system for ordering series of nights may be the oldest, as it is reminiscent of the numbering lists from West Polynesia and other areas. Finally, other forms may also reflect how suitable for fishing a night was: one Tahitian account describes the *Korekore (literally ‘Lack(ing)’) series as nights “when the fish disappear” [12], though over time such names may have been reinterpreted in some islands.

The archipelagos of East Polynesia used variations of the list of ‘nights of the moon’, which, while related, show significant differences and imply divergence over time. Historical changes in the names of the nights and their sequence may be due to loss of collective memory and reinterpretation of the meaning of names. For instance, in the Tuamotu and the southern group of the Cook Islands, *Vari*, meaning ‘menstrual blood’, replaced *(*ʔ*)*Ari*, possibly after the original name became semantically opaque. The duration of the lunar cycle (roughly 29.5 nights) may also have contributed to the changes. Ideally, calendrical lists included 30 nights (some even contained 31 names), but in certain months the new moon would eventually appear one night earlier, which probably meant that one name had to be removed from the count [7]. Such oscillations might account for the ‘blending’ of night names seen in some calendars: e.g., *Roŋo Taane* (Rapa Nui) combined *Roŋo and *Taane, *Roŋo-Mauri* (several locations) combined *Roŋo and *Mauri, and *Korekore-piri-Tangaroa* (New Zealand), literally ‘Korekore-joining-Tangaroa’, occurs at the transition from the *Korekore to the *Taŋaroa series. Series were expanded or created anew and some nights were dropped. Variant names with extra descriptors, perhaps originally optional, came into use (see, e.g., *Mauri > *Mauri-Kero or *Mauri-mate, both meaning roughly ‘Dying Mauri’, when the moon was at the end of its waning phase). Another common type of divergence, more likely to be due to memory loss, is the switching of the positions of the nights: for example, we find derivatives of *(*ʔ*)*Ari* both immediately before and after derivatives of *Funa in different lists.

The similarities and differences between lists of nights of the moon from all parts of East Polynesia have long been examined comparatively to explore their relationships and common origins [7,16,17]. Early discussions mainly followed conventional and qualitative philological approaches, focusing on representative sets of lists. However, they overlooked some documented evidence and only partially addressed certain issues with the sources, such as transcription errors and interpretation of cognate relationships between night names. One early commentator [7] concluded that New Zealand, the Marquesas, Mangareva, and Rapa Nui had “a similar sequence”, despite noting some confusion, which he attributed to defective recording and disruption in oral transmission. This conclusion does not align with any current hypothesis of settlement sequence or language classification for East Polynesia. Names of nights have also been considered as evidence for the sub-grouping of East Polynesian languages [18–20], but strictly in terms of lexical or phonological changes, and not systematically.

To address the limitations of previous studies on the relatedness among lunar calendrical lists, we applied a computational and phylogenetic method to a dataset that is, to our knowledge, more detailed and complete than any previously available in the literature.

## Materials and methods

### Dataset

Our dataset (S2 Dataset; sources detailed in S1 Appendix), contains 48 ‘lists of the nights of the Moon’ from all the major archipelagos and transmitted in all East Polynesian languages, except for the Rapan-speaking island of Rapa Iti: Aotearoa/New Zealand (n = 10) and Moriori/Chatham Islands (n = 1), Hawai’i (n = 2), Mangareva (n = 1), the Marquesas (n = 8), Raivavae in the Austral Islands (n = 1), Rapa Nui/Easter Island (n = 4), the southern (n = 5) and northern Cook (n = 3) groups, Tahiti and neighboring islands (n = 4), and the Tuamotuan archipelago (n = 9). Therefore, it is representative of the divergence of the lists throughout East Polynesia.

### Analysis of cognacy

We define cognate names (or cognate components of names) of nights as those that (1) originate from the same etymological source and (2) occupy a similar relative position in the calendrical list, regardless of whether their phonological form in a given East Polynesian language strictly follows that language’s regular sound laws. This cognacy relationship is similar to the connection between the names for ’January’ in different languages, such as French *janvier*, German *Januar*, Italian *gennaio*, Portuguese *janeiro*, and Spanish *enero*.

Etymologies used to determine (1), as per above, follow for the most those of POLLEX (Polynesian Lexicon Project) Online. POLLEX is a large-scale comparative dictionary of Polynesian languages, which uses reference dictionaries and other scholarly works as primary sources [15]. One notable exception involves a series of nights attested in lists from many locations, and whose names were already identified as cognates by J. F. Stimson in the 1930s: Marquesan *Maheama*, Mangarevan *Ma*[*ʔ*]*ema*, Tahitian *Hamiāma*, Rurutu (Austral islands) *ʔamia*, Tongarevan *Samia*, Tuamotuan *Hania* or *Hanīa* [21]. Stimson assumed the following development: *Samia* > *Hamia* > *Hamia-ma*(-*tahi*, etc.)> *Mahiama* > *Maheama* / *Ma*[*ʔ*]*ema*. Yet this leaves the etymology of the name unexplained, as most forms are opaque. We argue that the path was the opposite. The starting form is Proto-East Polynesian *ma-seʔa ‘(be) perceptible’ [15], originating the structure *maseʔa(*-maa*)-NUMERAL, where *maa is a numeral connective particle that became lexicalized and fossilized as part of the names of nights only in some calendars. Thus, *Maseʔa(-*maa*)- developed not just into Marquesan *Mahea-ma* / Mangarevan *Ma*[*ʔ*]*ema* (with *s > *h*), but also into *Same[*ʔ*]*a*(-*ma*), which, following metathesis (and the irregular shift *e* > *i*) finally became Tongarevan *Samia*, Tahitian *Hamiama* (with *e* > *i*), and Tuamotuan *Hamia > *Hania* (irregular *m* > *n*). The etymology is supported by the semantics of Hawaiian *māhea* ‘hazy (moonlight)’, Rapanui *maʔeha* ‘brightness; lighten/brighten up’, and Tahitian *maheahea* ‘turn pale; fade’, keeping in mind that this series of nocturnal names referred to some of the first nights after the new moon, when the latter is barely visible.

In addressing the proximity between any pair of calendrical lists, we did not just compute which forms of night names, and how many, were shared (the ‘lexical’ aspect). We also considered how much the relative position of a pair of cognate forms diverged within the sequence of ca. 30 nights (the ‘structural’ aspect). Moreover, we analyzed cognacy among names or components of names (here referred to as ‘forms’) of the lunar nights, rather than focusing on segments or phonemes (see next subsection).

### Notation of the data

For compiling the dataset, our notation of the names of lunar nights from the calendrical lists followed seven (1–7) principles:

[1] *The basic unit of analysis is a ‘form’***,** which represents the name of a night or part thereof. This choice responds to the intricacies of the data. On the one hand, in Polynesian studies (especially in early or non-linguistic publications) the notation of morphemes varies, leading to instances where the same combination of morphemes can be spelled as one or multiple words: e.g., *Rongo nui* / *Rongo-nui* / *Rongonui* ‘Big Rongo’. On the other hand, many night names are composite. Therefore, our unit of analysis comprises *any name or part of a night name that we can examine separately for its presence or absence across calendrical lists*. This unit, which we termed ‘form’, comprises both full words and morphemes, as well as repeated parts of morphemes (such as ‘ko’, which is the partial reduplication of the morpheme ‘kore’ in ‘kokore’). We defined our set of forms inductively, by examining the dataset and the established relations of cognacy among night names (see the index of forms in S1 Appendix).
[2] Forms (not character strings) were compared as whole units and notated in normalized ways—either reconstructed proto-forms or language-specific reflexes—disregarding phonological variation and linguistic change. For instance, East Polynesian *Roŋo [15] appears as *Roŋo* (Maori, Rapanui, and Tuamotuan), *ʔOno*, *ʔOko* (Marquesan), *Lono* (Hawaiian), and *Roʔo* (Tahitian) in different lists and languages. To encode all these forms as cognates, we notated them as ‘roŋo’, after the proto-form.
[3] The ‘nights of the moon’ were often recited using extended formulas: e.g., Tahitian *ʔO Roʔo-nui te pō* “Roʔo-nui is the night”, Maori *He Otāne* “It is O Tāne” (where O Tāne is literally “[night] of Tāne”), or Rakahanga-Manihiki *Ko maraŋi* “Marangi”, with a prominence marker (see S1 Appendix). Thus, in considering only basic forms to compare the names of nights, we excluded from the analysis most grammatical words (‘particles’) such as markers *ko* / *ʔo*, *he*, *o*, and the article *te*.
[4] Use of symbol ‘/’: Where there are variants of a form that can be diagnostic of relatedness between lists, both ‘form’ and ‘variant’ were annotated for computational analysis, following the scheme FORM/VARIANT. For example, while most calendrical lists have a reflex of *ʔAri, Mangaian, Rarotongan and Tuamotuan lists have *Vari*, probably due to lexical reinterpretation (as mentioned above). The presence of one or the other variant was notated as ‘ʔari/ʔari’ vs ‘ʔari/vari’. The part before ‘/’ shows that some (any) form of the night occurs, while the part after ‘/’ indicates the specific variant. They are distinct features, each of which may or may not be shared by any given pair of lists.
[5] Use of symbol ‘#’: This was used for two purposes: (i) the comparison of lexicalized complexes of the type of FORM#FORM against a single FORM (e.g., ‘maseʔa#maa’ is fully cognate with another ‘maseʔa#maa’ but only partially cognate with ‘maseʔa’); (ii) the comparison of reduplicated vs partially reduplicated vs non-reduplicated expressions of the same form; notice that reduplication is a common morphological process in Polynesian languages and it affects some night names (e.g., ‘kore#kore’ vs ‘ko#kore’ vs ‘kore’).
[6] Use of symbol ‘_’: This was used to detect full cognacy between composite night names of the type of FORM_FORM, regardless of the fact that the forms changed syntactical position (especially in ordered series of nights). For instance, different Tuamotuan lists feature the variant names *Hania fakaoti* ‘Final Hania’ and *Te fakaotiga na Hania* ‘The finalizing of Hania’, but we treated them as cognate despite changes in word order (and exact grammatical form), so as not to generate artifacts.
[7] Added numbers (FORM1, FORM2, etc.) distinguish non-cognate homographic forms. To illustrate this point: some lists share the composite ‘roŋo#nui’ while others have a standalone ‘roŋo’, a subtle difference we needed to consider. Similarly, we needed to compute the greater proximity between nights called ‘fotu#nui’ as opposed to nights called simply ‘fotu’. However, we did not count as cognates ‘fotu#nui’ and ‘roŋo#nui’. Even though both forms are modified by an adjective *nui* ‘big’, they are nights with quite different positions in their lists and the addition is most probably a trivial innovation that occurred independently. To put it simply, the addition of the adjective ‘big’ to *Fotu is a separate phenomenon from its addition to *Roŋo. To handle this, we added numerical labels to homographic words—both lexical and grammatical—to mark them as non-cognate forms: e.g., tuu#nui1 vs. tamatea_nui2 vs. fotu/fotu#nui3, etc.; tuu#faka1#so#ʔata vs faka2#pau vs faka3#ʔoti, etc. (see S1 Appendix).

### Exclusion of other calendrical types

We did not explore the divergence between the ‘naming’ lists of East Polynesia and the ‘numbering’ lists of West Polynesia and other areas, although some of the latter include a few related forms (e.g., there are cognates of *tuu, *matofi, *fakaʔoti, *mate, etc.; see S1 Appendix). Three reasons hinder the application of our comparative method to a set containing both types. First, while we have complete records of lists for several locations that use the ‘numbering’ type (Pukapuka, Rennell and Bellona, Samoa, Takuu, Tokelau, Tonga, and Vaitupu), in other instances we located only partial data (Nanumea, Niue, and Sikaiana). Second, in some cases nights were described with long sentences (e.g., the 28th night in the Pukapukan list is *Koa wakatau wenake ma te ulu o te ata matua* “[The moon] rises at the same time as the darkness before dawn”) making it difficult to isolate diagnostic and comparable lexical forms. Third, and most importantly, number words are pervasive in most West Polynesian and other lists, with some sequences of nights numbered from ‘one’ to as many as ‘ten’. This creates a risk for our method, as it might falsely indicate greater similarity with the East Polynesian calendars that have longer numbered series (such as those from Rapa Nui), which may be an independent innovation.

We also did not apply our method to the year calendar used in both West and East Polynesia, which had months also with specific names [1,7,13]. This calendar has been the focus of comparative studies and is found in similar forms across several archipelagos, but some locations lack comprehensive documentation or show disruptions. Notably, the Marquesan lists suffer from transcription issues, and the surviving Rapa Nui month names have minimal to no similarities with other Polynesian calendars. Moreover, establishing cognacy and patterns of divergence is challenging due to the practice of renaming months after the stars marking their beginning in certain islands.

### The issue of borrowability

While the lexicon is typically the most easily borrowed part of a language—and lunar calendars could in principle fall into this category—the high degree of isolation of many East Polynesian islands makes the borrowing of individual lunar night names unlikely in most cases. Indeed, we found little linguistic evidence for horizontal transmission (borrowing) in our dataset. We examined the lists for forms of night names that potentially did not conform to known patterns of historical phonology in the local language but instead matched those of another archipelago; yet, we were unable to identify any compelling cases. Moreover, calques—that is, night names borrowed from another island’s language but adapted to the local phonology—are virtually impossible to detect.

Nevertheless, it remains plausible that borrowing of night names and mutual influence occurred within specific archipelagos. In cases in which contact was significative in relatively recent times, the wholesale borrowing of calendrical lists is conceivable (see below on historical contact between Tongareva and Rarotonga). Accordingly, we should note at the outset that the Society, Tuamotu, southern Cook and Austral islands represent some of the least isolated archipelagos within East Polynesia. In addition, during the late 18th and early 19th centuries a newly-formed Tahitian kingdom expanded its influence in the region [22]. This facilitated increased contact across these islands. As a result, the calendrical lists from Tahiti, Tuamotu, the Southern Cook, and Raivavae islands are the most likely candidates to have undergone horizontal transmission, a factor we took into account when evaluating the phylogeny derived from our analysis. The case of Tongareva (Northern Cook) is also noteworthy. Although the island is geographically closest to Rakahanga and Manihiki, historical records indicate that it maintained close contact with Rarotonga (Southern Cook) for nearly seven decades (1864–1929) before its calendrical list was collected [23]. Therefore, we considered the possibility that the Tongarevan calendar (NCOOK_TGV) reflects Rarotongan interference. Continued contact with Rarotongan-speaking communities may also explain why a recent source registers the first lunar night in Tongareva as Tiroe (instead of older Tireo), a form otherwise only attested in the Atiu atoll (Southern Cook; see list 27 in S1 Appendix). In this instance, however, an earlier version of the Tongarevan list is preserved that contains the older and regular form (*Tireo*). This version—without the potential borrowed night—was the one used in our study.

### Computational comparison of pairs of names of nights

To quantify similarity/difference between forms of night names in any pair of calendrical lists, we applied an algorithmic method that yielded numerical distances (see below). First, we introduced an adapted version of the Edit Distance metric to compare single forms of names. To find the probable cognate names of nights across pairs of lists, we produced a matching between nights with the lowest cumulative distance (i.e., the sum of all distances between matched nights). This yielded a first measure termed ‘lexical similarity’ (ℒex). Second, the algorithm analyzed how lexically cognate nights differ in their relative positions in any pair of lists, with the aim of computing the lists’ structural divergence (𝒮truct). The algorithm considers the cyclical pattern of lunar calendars: for example, if two nights are found in the 29th and 30th positions in one list, but their counterparts in another list occur in the 30th and 1st positions, respectively, this means that their relative positions are the same. Lastly, we combined the measures ℒex and 𝒮truct to obtain the final distance between any pair of lists (see below).

**Fig 2:**
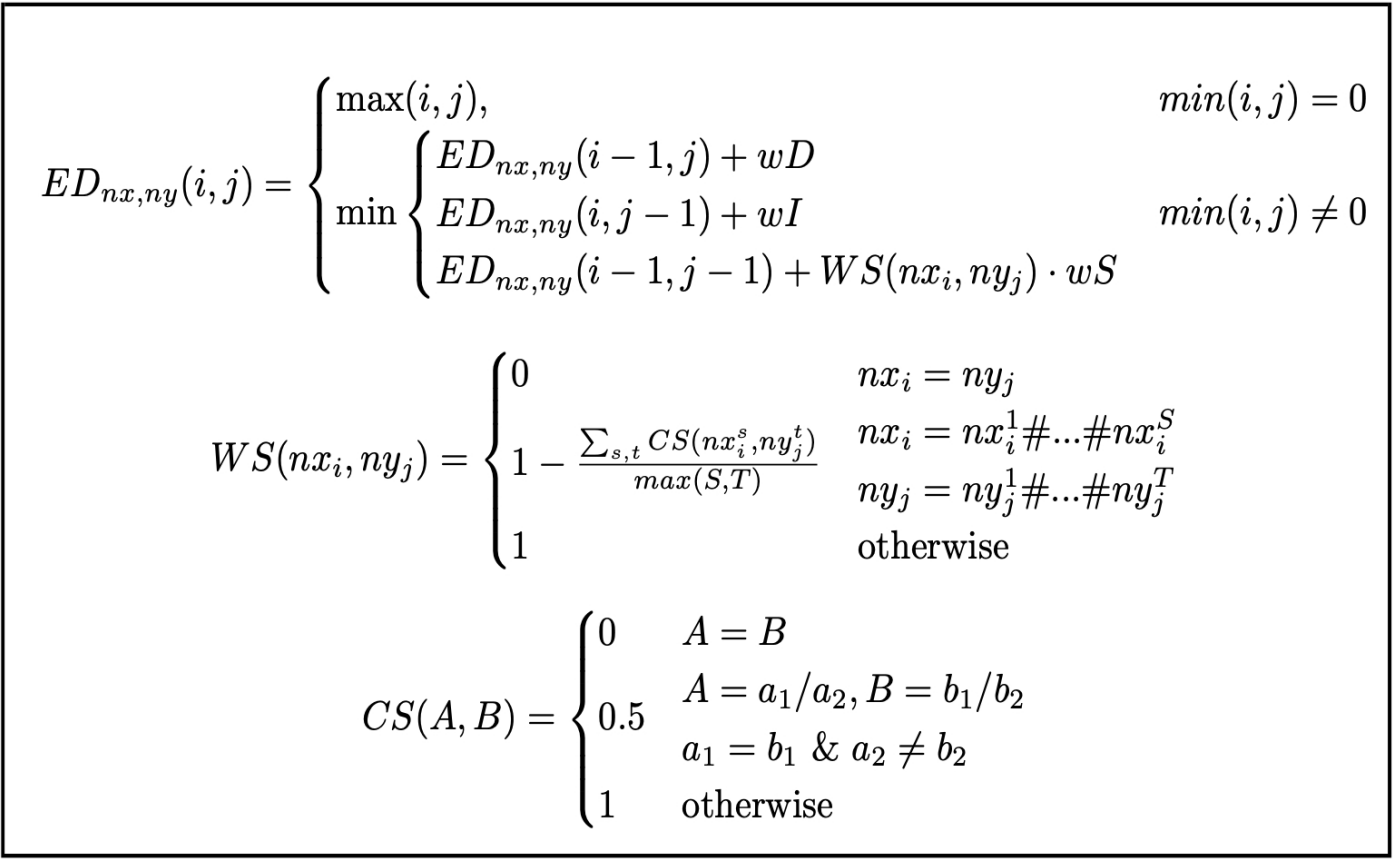
Definition of the Edit Distance: *wD*, *wI*, and *wS* represent the weight penalties for sign deletion, insertion, and substitution, respectively. In this study, *wD* = *wI* = 1 and *wS* = 2.

We represented ‘forms’ (i.e., names or parts of names of calendrical nights, as collected in S1 Appendix and S1 Dataset) as $n=\langle n_{1},...,n_{w}\rangle$. To accurately identify cognate names of nights, even in cases where linguistic phenomena changed the syntactical order of forms within a name (e.g., Tuamotuan *Hania fakaoti* and *Fakaoti-Hania*), we processed every possible permutation: $P^{n}=\{P_{1}^{n},...P_{w!}^{n}\}$. As the name of a night is comprised of only 1–4 forms, combinatorial explosion was not an issue. We compared pairs of names using a variation of the well-known Edit Distance, as defined in Fig 2.

The function was extended to compute similarity between forms (notating them as described above). The general Edit Distance, including our variation, does not consider word length and is not ideal for comparing the distance between sets of words. Thus, various studies have introduced a form of normalization for Edit Distance values. We relied on the valuable properties [24] of the *Generalized Edit Distance* (as proposed and analyzed by [25]): it is a metric (which means it is non-negative, symmetric, and it satisfies the triangle inequality), its upper bound is 1, and it does not escalate repetitions. Thus, we normalized $ED_{nx,ny}$ as


$$\begin{array}{c} {\overset{―}{ED}}_{nx,ny}=\frac{2\cdot ED_{nx,ny}}{|nx|+|ny|+ED_{nx,ny}} \end{array}$$

where |·| represents the number of compounded forms, separated by the symbol ‘_’, and forming the name of a night (e.g., |faka1#ʔoti#ŋa1_maseʔa/samia| = 2). Given also the need to address trivial grammatical phenomena (including word-order changes), we defined the distance between the names of two nights as


$$\begin{array}{c} ND_{nx,ny}=min_{P_{i}^{nx},P_{j}^{ny}}\;{\overset{―}{ED}}_{P_{i}^{nx},P_{j}^{ny}} \end{array}$$

searching for the pair of permutations of the two names of nights that have the minimal distance.

### Lexical measure

Relations of cognacy between names of nights from two calendrical lists entail 1-to-1 mappings between cognate nights and require finding matching pairs across lists. To address this and facilitate accurate evaluation, we relied on the standard *Linear Sum Assignment* (LSA) problem for matching the calendars’ cognates, which can be solved by the *Hungarian algorithm*. We defined $C_{X}=\langle nx_{1},...,nx_{K}\rangle$ and $C_{Y}=\langle ny_{1},...,ny_{M}\rangle$, that is, any two calendrical lists to be compared, as two tuples of nights. We also assumed that K≤M, without any loss of generality, enabling the comparison of lists with different numbers of items. We can define a matching solution $\Xi_{C_{X},C_{Y}}=\langle \xi_{i,j}\rangle$ as a matrix over the variables $\xi_{i,j}\in \{0,1\}$ representing the calendar alignment obtained by the LSA algorithm, with $\xi_{i,j}=1$ if and only if $nx_{i}$ is assigned to $ny_{j}$ and 0 otherwise. The LSA problem to be solved can then be expressed as


$$\begin{array}{c} ℒex_{C_{X},C_{Y}}=\frac{1}{K}\min\;\sum_{i=1}^{K}\sum_{j=1}^{M}\xi_{i,j}\cdot ND_{nx_{i},ny_{j}} \end{array}$$

$$\begin{array}{c} \text{s.t. }\sum_{i=1}^{K}\xi_{i,j}\leq 1,\;j=1,2,...,M\\ \sum_{j=1}^{M}\xi_{i,j}=1,\;i=1,2,...,K \end{array}$$

Note that: if K≤M, then M−K nights are not matched.

### Structural measure

The Lee Distance is a metric and for two general permutations of integers *p*_1_ and *p*_2_ of length *n* is:


$$\begin{array}{c} \delta (p1,p2)=\sum_{e\in p_{1}}min(|i-j|,K-|i-j|),\text{ where }p_{1}(i)=p_{2}(j)=e \end{array}$$

Considering that a LSA matching can be expressed as two permutations of positions of nights in $C_{X}$ and $C_{Y}$, it can be adapted to our problem as


$$\begin{array}{c} \delta (\Xi_{C_{X},C_{Y}})=\sum_{\xi_{i,j}=1}min(|i-j|,K-|i-j|). \end{array}$$

and then (following [26]) normalized as


$$\begin{array}{c} \overset{―}{\delta (\Xi_{C_{X},C_{Y}})}=\frac{\delta (\Xi_{C_{X},C_{Y}})}{\left(K*\lfloor M/2\rfloor \right)}. \end{array}$$

In some instances, two lists are identical except for the presence of an extra night (e.g., RPN1 and RPN3). In these cases, $\delta (\Xi_{C_{X},C_{Y}})$ is not a faithful measure as it counts a single shift multiple times. Thus, our method fixed the first list and shifted the nights of the second list in search for the best rotational matching:


$$\begin{array}{c} \delta (\Xi_{C_{X},C_{Y}}{)}_{k}=\sum_{\xi_{i,j}=1}min(|i-(j+k)|,K-|i-(j+k)|). \end{array}$$

Moreover, typically there was not a single best matching solution for any pair of lists, so we had to explore a set of equivalent solutions having the same ℒex value but different matching $\Xi_{C_{X},C_{Y}}$, and forming a set of possible solutions $\left\{\Xi_{C_{X},C_{Y}}^{z}\right\}$.

The final structural distance 𝒮truct can thus be defined as


$$\begin{array}{c} 𝒮truct_{C_{X},C_{Y}}=min_{z}\left[min_{k}\;\overset{―}{\delta (\Xi_{C_{X},C_{Y}}^{z}{)}_{k}}\right]. \end{array}$$

**Calendrical distance**

The final calendrical distance combined the lexical (ℒex) and the structural (𝒮truct) measures. We did not simply sum their contributions. If two lists are very different, then their ℒex score would be high—close to 1—and most of the distances between individual pairs of nights would also be high. This means that the LSA matching will, correctly, produce a high number of equivalent but poorly matching solutions, with nights matched almost randomly. If we searched for the best matching through double minimization in the definition of 𝒮truct, we would certainly find a suitable solution with a very low 𝒮truct score. This is evidently incongruous: the meaningfulness of the measure 𝒮truct had to be modulated in some way to avoid such degenerate behavior. Given that the contribution of the 𝒮truct distance must be maximal for ℒex near 0 and minimal for ℒex near 1, for deriving the final distance between two calendars $C_{X}$ and $C_{Y}$ we can combine the two measures as


$$\begin{array}{c} 𝒟ist_{C_{X},C_{Y}}=ℒex_{C_{X},C_{Y}}+(1-ℒex_{C_{X},C_{Y}})\cdot 𝒮truct_{C_{X},C_{Y}} \end{array}$$

Note that $𝒟ist_{C_{X},C_{Y}}$ was built starting from the Generalized Edit Distance ${\overset{―}{ED}}_{nx,ny}$ and the Lee Distance (both provably metrics), only using additions and multiplication by positive scalars, so that it can be proved that $𝒟ist_{C_{X},C_{Y}}$ is also a metric. The aim of this distance is precisely to create a metric that reduces the impact of the structural measure when the lexical one is very low, as a structural comparison between calendars with very little similarity in terms of the names of the nights does not constitute a meaningful measure. For this reason, the structural component in the formula is scaled by one minus the lexical component.

By combining the metrics described above to derive $𝒟ist_{C_{X},C_{Y}}$, we were then able to compare every calendrical list and produce a distance matrix suitable for the application of an algorithm that enabled us to construct a phylogenetic tree, as described next.

### Construction of the phylogenetic tree

**Fig 3:**
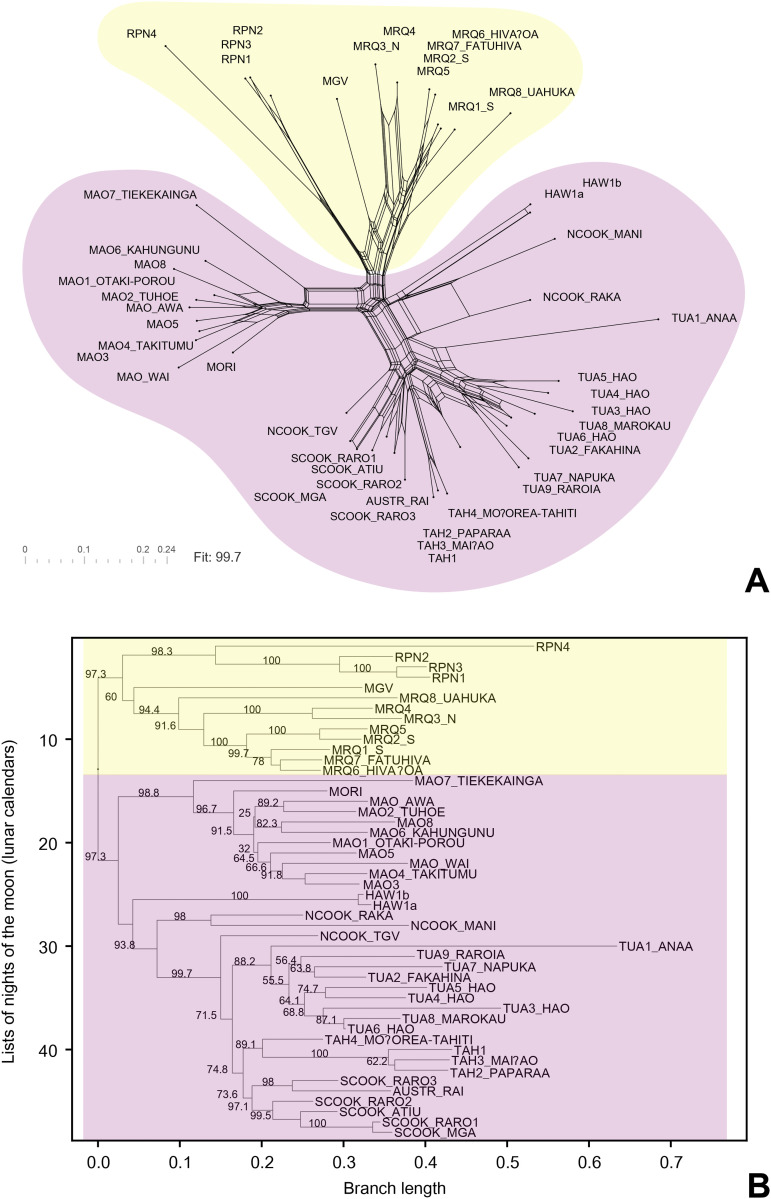
Phylogeny of 48 lists of ‘the nights of the moon’ from East Polynesia: split network graph using NeighborNet (A) and rooted phylogenetic tree using the MAD algorithm, with indication of node confidence values (B). Key to the macro-provenances (islands or archipelagos) of the calendrical lists: AUSTR: Austral Islands; HAW: Hawai’i; MAO: Aotearoa/New Zealand; MGV: Mangareva (in the Gambier archipelago) MRQ: Marquesas Islands; MORI: Moriori/Chatham Islands; NCOOK: Northern Cook Islands; SCOOK: Southern Cook Islands; RPN: Rapa Nui/Easter Island; TAH: Tahiti and neighboring islands (Society Islands, French Polynesia); TUA: Tuamotu Islands. See S1 Appendix for the specific source of each list. Pale yellow and light purple highlight the lists corresponding to the two main branches of the rooted tree, Branch 1 and Branch 2, respectively.

Before constructing a phylogenetic dendrogram, we assessed the extent to which our calendrical distance data were compatible with a tree-like representation. To this end, we used SplitsTree App [27] to calculate delta scores (Table 2) and to produce a split network graph (Fig 3A) based on the Neighbor-Net algorithm [28] from our distance matrix.

**Table 2:** Delta scores for the dataset of 48 East Polynesian lists of nights of the moon. Also included are the values for Q-residuals, which quantify departures from a strict tree (lower values indicate a better fit of the data to a phylogenetic tree) [29].

List no.	List label	Delta score	Q-residual
1	AUSTR_RAI	0.293400	0.013845
2	HAW1a	0.275940	0.009044
3	HAW1b	0.280880	0.009484
4	MAO1_OTAKI-POROU	0.236550	0.010222
5	MAO2_TUHOE	0.217370	0.008026
6	MAO3	0.213370	0.008856
7	MAO4_TAKITUMU	0.222830	0.008947
8	MAO5	0.222920	0.008324
9	MAO6_KAHUNGUNU	0.242290	0.010262
10	MAO7_TIEKEKAINGA	0.246260	0.010898
11	MAO8	0.230150	0.009460
12	MAO_AWA	0.227230	0.008527
13	MAO_WAI	0.239370	0.010651
14	MGV	0.250700	0.010906
15	MORI	0.235950	0.010043
16	MRQ1_S	0.241740	0.009825
17	MRQ2_S	0.215540	0.009420
18	MRQ3_N	0.226550	0.010843
19	MRQ4	0.212490	0.009764
20	MRQ5	0.223490	0.009015
21	MRQ6_HIVAʔOA	0.222720	0.009395
22	MRQ7_FATUHIVA	0.238340	0.010802
23	MRQ8_UAHUKA	0.247410	0.012306
24	NCOOK_MANI	0.300190	0.011536
25	NCOOK_RAKA	0.307320	0.013355
26	NCOOK_TGV	0.310630	0.015042
27	SCOOK_ATIU	0.258050	0.011058
28	SCOOK_MGA	0.282300	0.011060
29	SCOOK_RARO1	0.259980	0.009947
30	SCOOK_RARO2	0.256440	0.010676
31	SCOOK_RARO3	0.259250	0.010881
32	RPN1	0.237830	0.010767
33	RPN2	0.246460	0.010899
34	RPN3	0.242550	0.011071
35	RPN4	0.248860	0.011993
36	TAH1	0.272610	0.011989
37	TAH2_PAPARAA	0.282880	0.012731
38	TAH3_MAIʔAO	0.304170	0.016537
39	TAH4_MOʔOREA-TAHITI	0.283160	0.013536
40	TUA1_ANAA	0.317640	0.014858
41	TUA2_FAKAHINA	0.246550	0.011099
42	TUA3_HAO	0.308580	0.014903
43	TUA4_HAO	0.246390	0.010718
44	TUA5_HAO	0.259460	0.012773
45	TUA6_HAO	0.244130	0.012138
46	TUA7_NAPUKA	0.261080	0.010702
47	TUA8_MAROKAU	0.255990	0.011107
48	TUA9_RAROIA	0.256910	0.011041

A split graph visualizes both tree-like and conflicting signals in the data: branching structures indicate tree-like relationships, whereas box-like structures indicate conflicting arrangements. In our network, most reticulations are confined to lists from the same archipelagos or islands, notably the Marquesas, New Zealand/Chatham Islands, the South Cook Islands, and the Tuamotu (Fig 3A). This pattern is consistent with local interaction and possible horizontal transmission among geographically proximate communities, but does not obscure the overall tree-like structure of the dataset. More substantial reticulation is observed in the central region encompassing the Society, Austral, South Cook, and Tuamotu archipelagos. As stated above, this is precisely the area for which historical evidence suggests sustained inter-island contact from the late 18th onwards.

Delta scores, introduced by Holland et al. [30], provide an exploratory measure of the extent to which the data from a distance matrix departs from a tree-like structure. The method assigns values between 0 and 1 to individual taxonomic units according to the extent to which each ‘taxon’ is involved in conflicting phylogenetic signals. These unit-specific values can then be averaged to obtain an overall estimate of the tree-likeness of the dataset, with higher values indicating a greater degree of conflicting signal and reticulation [29]. Crucially, as Gray et al. [29] noted, there is no generally accepted method for assessing the statistical significance of delta scores. The average delta score of our calendrical dataset is 0.2544, which is substantially lower than the values reported for applications of the method to datasets of Indo-European and Austronesian languages by the same authors [29]. This suggests that our calendrical data does not exhibit a high level of conflicting signal and is sufficiently tree-like to support phylogenetic reconstruction.

We therefore produced a dendrogram by applying the Neighbor-Joining algorithm [31] to the matrix containing all pairwise distances. The resulting tree was rooted using the MAD (Minimal Ancestor Deviation) algorithm [32], which has been shown to outperform other rooting methods based on experiments with biological phylogenies. An additional reason for adopting this method is its robustness to evolutionary rate heterogeneity. This property is particularly relevant in the present study, as we made no a priori assumptions about the tempo of change of the calendar systems we investigated. Finally, MAD rooting is especially useful when a suitable outgroup is unavailable. This is the case here, since, for the reasons discussed above, we considered it unfeasible to use the numbering-based calendrical lists from West Polynesia and other regions as an outgroup. With regard to the applicability of the Neighbor-Joining clustering method and the MAD rooting algorithm to our distance matrix: the assumptions underlying Neighbor-Joining (namely, positivity, symmetry, a zero diagonal, and additivity of distances) are satisfied by the fact that $𝒟ist_{C_{X},C_{Y}}$ is a metric; the same preconditions hold also for the MAD algorithm [32].

After rooting the tree with MAD, we assessed the robustness of the inferred topology by estimating node confidence values using residual resampling. We followed the approach proposed by Waddell and Azad [33], which calculates residuals as the differences between the observed pairwise distances and the distances implied by the fitted tree. These residuals are then resampled with replacement to generate replicate distance matrices and reconstruct a Neighbor-Joining tree from each replicate. Ultimately, node confidence values correspond to the proportion of replicate trees in which a given clade was recovered. The resulting values indicate strong overall support for the inferred topology of our tree (Fig 3B).

### Code availability

The code and data needed to repeat the analysis, and generate all figures and intermediate results are available from https://zenodo.org/records/20702913.

## Results and discussion

Our tree (Fig 3B) accurately reflects the similarity between lunar night lists from geographically close areas. The sets from New Zealand, the Tuamotuan archipelago, Tahiti (and nearby islands), the Marquesas, and the southern Cook were all grouped together in specific branches or sub-branches. Moreover, the four lists from the Tuamotuan atoll of Hao not only appear closely related, but also share a common node (‘ancestor’) with the list from the neighboring atoll of Marokau. The Maori lists attributed to the Awa and Tuhoe tribal groups, both from the northeastern part of North Island (New Zealand), also branch out from the same node. The lists from the southern Marquesas are located in a separate sub-branch of the tree, divided from those of the northern Marquesas. Our method also succeeded in recognizing the correct phylogenesis of one ‘corrupted’ list from Rapa Nui (labeled RPN4) and two lists reconstructed from relatively late dictionary entries (TUA1, RARO3; see S1 Appendix for details). However, as also expected, the tree places these very divergent lists (RPN4, TUA1) at considerable distances to other taxonomic units with common nodes (i.e., ‘ancestor’ lists). Moreover, TUA1 shows the highest delta score, indicating greater conflicting signal and suggesting possible horizontal interference from other calendars.

Our phylogeny also aligns with the real distribution of shared vs non-shared night names. The tree has two main branches or groups of calendrical lists (Branch 1 and Branch 2), consistent with an ancient two-way split of the proto-lunar calendar. Branch 1 includes the lists from the Marquesas, Mangareva, and Rapa Nui, while Branch 2 encompasses the calendars from New Zealand, Hawai’i, the Cook and Austral islands, Tahiti, and the Tuamotu. We observe two key distinctions between the lists in these larger branches. First, those in Branch 1 feature a single *Raakau night, either before or after a night called *Matofi, whereas those in Branch 2 have two or more *Raakau nights forming a series and found alongside *Matofi only sometimes. Second, none of the lists in Branch 1 contains a night whose name is a reflex of *Tamatea. Conversely, all sub-ramifications of Branch 2 include a night or series of nights named *Tamatea, except for those from Hawai’i (although it is possible that *Tamatea originally existed in Hawai’i and was later substituted and dropped). When we consider further subdivisions of the tree, we find that in general they agree well with shared or non-shared features among calendars. Another advantageous property that emerged is that the ancestor of the Hawaiian lists is placed the closest to the ancestor of the Rakahanga and Manihiki ones, which agrees with their geographical positions (Fig 1) and a scenario in which Hawai’i was settled by migrants from islands located to the southwest of the archipelago.

Only the sub-branch with the Tuamotuan, Tongarevan, southern Cook and Tahitian lists poses issues. In these lists night series are ordered using *roto ‘middle’, probably a shared innovation. Yet, while most follow the pattern *tahi, *roto, *(*faka*)*ʔoti* ‘one, inside, final’, three of the Tahitian lists use *muʔa, *roto, *muri ‘before, inside, after’. In addition, the sets from Tongareva and Tahiti have a night called *Ari*. This is shared with other sub-branches of Branch 2 as well as Branch 1, and must therefore be a retention (‘archaism’). Conversely, the Tuamotu and southern Cook sets have *Vari* instead of *Ari*, likely an innovation (as noted in the Introduction). These features differentiate the Tahitian lists from those of the Tuamotu and southern Cook, but the tree does not imply the separate evolution of the Tahitian sets. Instead, it produces a common node (ancestor) for the Tahitian and southern Cook lists, and separates the Tuamotuan calendars. This result is not unexpected: as noted above, Tahiti exerted influence over neighboring archipelagos from the late 18th century onward, within a central area of East Polynesia that includes some of the least isolated islands. As a result, horizontal influence among calendars in this region is more likely. This may explain why the sub-branches do not show the Tahitian lists as more distinct from the others. Similarly, the fact that the Tongarevan list is not more closely aligned with the Rahakanga and Manihiki lists—despite their geographical proximity within the Northern Cook Islands—and instead clusters with the Tahitian, Tuamotuan, and Southern Cook sub-branch, is likely due to historically documented contact with populations from Rarotonga (Southern Cook) in the 19th century.

The sequence of settlement in East Polynesia is likely reflected—at least to some degree—in the historical development of both East Polynesian lunar calendars and languages, as both are symbolic systems transmitted and changed within communities. Although long-distance sea travel in the pre-industrial Pacific facilitated the settlement of Polynesia, it is important to remember the vast distances and significant isolation of many islands and archipelagos in the region, which greatly limited contact and borrowing. In this context, the current phylogeny of the lunar calendars offers implications worth considering.

As regards the historical development of East Polynesian languages, the most cited classification proposes a Proto-Central-Eastern Polynesian language, which first divided into Rapanui and Proto-East Polynesian. Proto-East Polynesian is subsequently divided into two major sub-groups: ‘Tahitic’ (including Tahitian, New Zealand Maori, Tuamotuan, etc.) and ‘Marquesic’ (comprising Hawaiian, Mangarevan, and Marquesan) [18]. More recently, two alternative classifications have been proposed. One, put forward by Walworth, suggests that Proto-East Polynesian first split into an isolated Rapa Nui branch and a Central Eastern Polynesian (CEP) group comprising all other languages. According to this proposal, the CEP languages did not divide into Marquesic and Tahitic groups as traditionally postulated, but rather developed through ongoing waves of contact driven by alleged high inter-island mobility, spreading linguistic features even to distant regions such as Aotearoa/New Zealand and Hawai’i [20]. The other alternative view, proposed by Wilson, suggests that Proto-East Polynesian split into two groups: a ‘Distal’ group, including Marquesan, Mangarevan, and Rapa Nui, and a ‘Proximal’ group, comprising the remaining languages, including Hawaiian [4].

**Fig 4:**
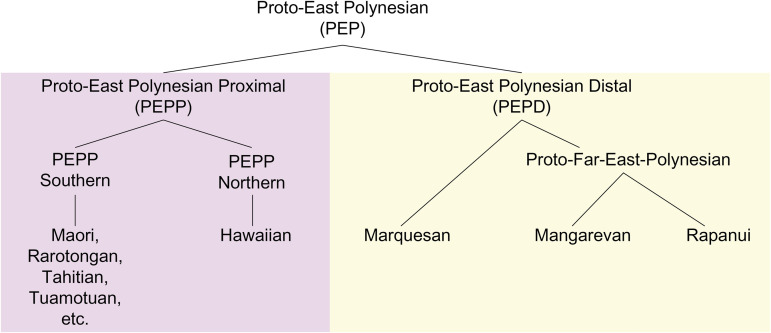
Wilson’s alternative subgrouping of East Polynesian languages into Proto-East Polynesian ‘Proximal’ (pale yellow) and ‘Distal’ divisions (light purple). Adapted from [4].

As it happens, our tree aligns with the division of East Polynesian languages in ‘Distal’ and ‘Proximal’ sub-groups. According to this classification, ‘Distal’ languages include Marquesan (with its two main dialects), Mangarevan, and Rapanui, while ‘Proximal’ languages comprise Hawaiian, Mangaian (southern Cook), Manihiki-Rakahanga, Moriori, New Zealand Maori, Tongarevan/Penrhyn, (Old) Rapan, Rarotongan (southern Cook), Tahitian, and Tuamotuan [4] (Fig 4). The archipelagos where these two linguistic subgroups were spoken closely match those where the lists in the two main branches of our calendrical phylogeny (those nearest the root of the tree) were used. Accordingly, our findings can be interpreted as evidence that the earliest major divergences in the East Polynesian proto-language and proto-lunar calendar occurred in parallel, likely reflecting an ancient movement of populations. In this scenario, speakers of ‘Proto-East Polynesian Distal’ used a lunar calendar ancestral to the Rapa Nui, Mangarevan, and Marquesan sets (the node of Branch 1), and their subsequent dispersal to these islands resulted in the parallel divergence of both their language and their lunar calendar. Likewise, we might infer that speakers of ‘Proto-East Polynesian Proximal’ used the ancestor to the remaining lists (node of Branch 2).

However, after this point our results do not necessarily align with Wilson’s linguistic phylogeny anymore. One tenet of his linguistic subgrouping is that ‘Proximal’ languages initially divided into ‘Southern’ and ‘Northern’ dialects, with the latter being ancestral to Hawaiian and the former giving rise to all other ‘Proximal’ tongues [4]. Conversely, our dendrogram indicates a greater divergence of the Maori (New Zealand) and related Moriori (Chatham Islands) calendrical lists with regard to the rest of areas where ‘Proximal’ tongues were spoken. This could simply reflect a higher degree of change in the New Zealand and Chatham calendars, but it may also warrant future linguistic re-examination of the relationship between ‘Proximal’ languages.

Finally, as with any phylogenetic approach applied to socially constructed systems, the method presented here entails assumptions and limitations. These concern, in particular, processes such as borrowing (discussed above) and uneven rates of change, which may not be fully captured in strictly phylogenetic approaches. Future work may therefore refine the modeling of the historical trajectory of East Polynesian calendars—for example, by exploring analytical methods that explicitly address possible horizontal transmission and convergence.

## Conclusions

The phylogeny of East Polynesian lists of “nights of the moon” presented here closely aligns with one proposed classification of East Polynesian languages, strongly suggesting parallel historical developments in both symbolic systems. We conclude that this correspondence is unlikely to be due to chance and reflects—at least to some extent—the pathways of human settlement in East Polynesia, with a probable two-way population split: one leading to the settlers of the Marquesas, Mangareva, and Rapa Nui, and the other to the settlers of the remaining East Polynesian-speaking archipelagos. This challenges previous models which propose that Rapanui was the first linguistic community to diverge from the rest. Although phylogenetic approaches have limitations in accounting for horizontal transmission, this issue is mitigated in the case of East Polynesia and its calendars by the great geographic isolation of the archipelagos and the limited evidence for borrowing of names of nights across islands. Potential exceptions are limited so far to the central regions, including the Society Islands (with Tahiti), the Tuamotu, the southern Cook and the Austral Islands. We also emphasize the independent nature of the evidence provided by the computational analysis of lexical and structural similarities across numerous calendrical lists, examined systematically here for the first time. These findings open a new perspective on the settlement history of East Polynesia and should be considered alongside archaeological, genetic, and linguistic data. Finally, the new and unique dataset of 48 calendrical lists presented here can serve as a foundation for further testing our proposal and for exploring alternative analytical approaches to the same data. It may also assist future efforts to reconstruct the original, unrecorded “proto-East Polynesian” list of the “nights of the moon.”

## Supporting information

S1 Appendix Documentary basis of the dataset, including calendrical lists, an index of forms, philological commentary, and bibliography. (PDF)

S1 Dataset Spreadsheet with the calendrical lists in notational form as used for computational analysis. (CSV)
