# Supplementary material for: Computational analysis reveals historical trajectory of East Polynesian lunar calendars
Source: PLoS One. 2026 Jul 29;21(7):e0353287. doi: 10.1371/journal.pone.0353287 (PMC13419179; doi:10.1371/journal.pone.0353287)
Supplement: S1 Appendix — (PDF) [file pone.0353287.s001.pdf]

S1 Appendix: Supporting Information for

Computational analysis reveals historical trajectory of East-Polynesian lunar calendars

S1A: The dataset and its sources

We investigated 48 lists of 'the nights of the moon' documented in all the major East Polynesian-speaking archipelagos and islands (with the exception of Rapa): Aotearoa/New Zealand (n=10) and Moriori/Chatham Island (n=1), Hawaii (n=2), Mangareva (n=1), the Marquesas (n=8), Raivavae in the Austral Islands (n=1), Rapa Nui/Easter Island (n=4), the southern (n=5) and northern Cook (n=3) groups, Tahiti and neighboring islands (n=4), and the Tuamotuan archipelago (n=9).

We primarily labeled the lists we collected in terms of macro-location (archipelago or island/atoll), followed, when necessary, by a micro-location (island/atoll within an archipelago, or region/district/tribe within an island), and a number. For instance: **RPN1** is our first and the oldest known list from Rapa Nui; **NCOOK_RAKA** refers to the list from the atoll of Rakahanga in the northern Cook Islands.

Key to the abbreviations of macro-locations:

- **AUSTR** = Austral Islands
- **HAW** = Hawaii
- **MAO** = Aotearoa/New Zealand
- **MGV** = Mangareva (in the Gambier archipelago)
- **MRQ** = Marquesas Islands
- **MORI** = Moriori/Chatham Islands
- **NCOOK** = Northern Cook Islands
- **SCOOK** = Southern Cook Islands
- **RPN** = Rapa Nui (Easter Island)
- **TAH** = Tahiti and neighboring islands (Society Islands, French Polynesia)
- **TUA** = Tuamotu Islands

Key to labels of micro-locations in Aotearoa/New Zealand:

- **AWA** = Ngāti Awa (Maori *iwi* or tribe), eastern Bay of Plenty Region
- **KAHUNGUNU** = Ngāti Kahungunu (Maori *iwi* or tribe)
- **OTAKI-POROU** = Ōtaki and Ngāti Porou (Maori *iwi* or tribes), North Island
- **TAKITUMU** = Takitumu district
- **TIEKEKAINGA** = Okirihau, i.e., Tieke Kāinga, in North Island
- **TUHOE** = Tūhoe (Maori iwi or tribe), Te Urewere, eastern North Island
- **WAI** = Waikato district

Key to label of micro-location Austral Islands:

- **RAI** = Raivavae

Key to labels of micro-locations in the Marquesas:

FATUHIVA = Fatu Hiva, island in the southern Marquesas
HIVAʔOA = Hiva Oa, island in the northern Marquesas
N = Northern Marquesas
S = Southern Marquesas
UAHUKA = Ua Huka, island in the southern Marquesas

Key to labels of micro-locations in the Northern Cook:

MANI = Manihiki, atoll where a Maori dialect termed Rakahanga-Manihiki is spoken
RAKA = Rakahanga, atoll where Rakahanga-Manihiki is also spoken
TGV = Tongareva / Penrhyn (atoll)

Key to labels of micro-locations in the Southern Cook:

ATIU = Atiu
MGA = Mangaia
RARO = Rarotonga (atoll)

Key to labels of micro-locations in the Society Islands / Tahiti:

PAPARAA = Paparā, location in the island of Tahiti
MAIʔAO = Mai'ao or Maiao, in the Windward Islands (eastern group of the Society Islands)
MOʔOREA-TAHITI = Mo'orea and Tahiti, in the Windward Islands (eastern group of the Society Islands)

Key to labels of micro-locations in the Tuamotu:

ANAA = Anaa, atoll in northwestern Tuamotu
FAKAHINA = Fakahina or Kaīna, atoll in northern Tuamotu
HAO = Hao, atoll in central Tuamotu
NAPUKA = Napuka, atoll in northeastern Tuamotu
MAROKAU = Marokau, atoll in central Tuamotu
RAROIA = Raroia, in central-northern Tuamotu

Importantly, we numbered Maori lists MAO1-MAO8 according to the same order in which they appeared in Best (1922) (*13*). Moreover, some lists have the same abbreviation and number, followed by a Latin letter in lowercase to indicate that they are variants of the same list, with only slight differences: HAW1a and HAW1b.

Here, we present the 48 calendrical lists from East Polynesian-speaking areas that constitute the dataset for this study. For each list, we give as tabulated text the following data: (a) the names of the 'nights of the moon' as found in the bibliographical sources, (b) the transcription of the names after necessary corrections (mainly of misprints and difficult orthographic or linguistic forms), and, finally, (c) their conversion into our annotation system as required for computational analysis. We provide the data along with the relevant references and supporting philological commentary. Finally, we detail our system of annotation and its criteria in section S1B.

List 1: AUSTR_RAI (Raivavae, Austral Islands). Reported by Edwards (2015) after Stimson, cited as: “Stimson, John Frank, Raivavaen Dictionary, unpublished incomplete manuscript with hand annotations by Donald Marshall and made public by David Mayer, original manuscript location is unknown but may be stored in the Phillips Library of the Peabody Essex Museum in Salem, Massachusetts, 1938” (*35*). The data reported surely reflects two or more different variant lists, including one that might show Tahitian or Tuamotuan influence, given the presence of nights called “Hiro Hiti”, “Maitu”, and “Rapu”. From this report we extracted for analysis the only coherent, sequential list that can be reconstructed:

	Original	Reconstructed	Normalized
1	Tireo / Hiro ~ Hiro Hiti ~ Hiro Tu Hiti	Tireo	tireo/tireo
2	Hiro ~ Hiro Hiti ~ Hiro Tu Hiti / Hoata	Hiro (Tu) Hiti	firo#fiti
3	Hoata / Hamiama (Tahi)	Hoata	so#ʔata
4	Hamiama (Tahi) / Roto o Te Hamiama	Hamiama (Tahi)	maseʔa/samia#maa_tahi
5	Roto o Te Hamiama / Ha’aoti o Te Hamiama	Roto o Te Hamiama	roto_maseʔa/samia#maa
6	Ha’aoti o Te Hamiama	Haʔaoti o Te Hamiama	faka3#ʔoti_maseʔa/samia#maa
7	Roto o Te ’Ore ’Ore	ʔOre ʔOre	kore#kore
8	Roto o Te ’Ore ’Ore / Ha'aoti o Te ’Ore ’Ore ~ ’Ore ’Ore	Roto o Te ʔOre ʔOre	roto_kore#kore
9	Ha’aoti o Te ’Ore ’Ore ~ ’Ore ’Ore / (O)Ari / Tamatea	Haʔaoti o Te ʔOre ʔOre	faka3#ʔoti_kore#kore
10	Huna	Huna	funa
11	(O)Ari / Rapu / Maharu	(O)Ari	ʔari/ʔari
12	Maharu	Maharu	maharu
13	Tireo / Ma Aitu ~ Ma Atua	Maitu / Ma Atua	maitu
14	Ma Aitu ~ Ma Atua / Hotu	Hotu	fotu/fotu
15	Marangi	Marangi	maa2#raŋi
16	Marangi / (O)Turu	Turu	turu
17	(O)Turu / Ra’au Tahi / Ti'a / Roto o Te Ra’au	Raʔau Tahi / Mua Raʔau	raakau_tahi
18	Ra’au Tahi / Mua Ra’au / Roto o Te Ra’au	Roto o Te Raʔau	roto_raakau
19	Roto o Te Ra’au / Ha’aoti o te Ra’au	Haʔaoti o te Raʔau	faka3#ʔoti_raakau
20	Ha’aoti o te Ra’au	(Mua) ʔOre ʔOre	muʔa_kore#kore
21	Mua ’Ore ’Ore /’Ore ’Ore	ʔOre ʔOre	kore#kore
22	Ha'aoti o Te ’Ore ’Ore ~ ’Ore ’Ore	Haʔaoti o Te ʔOre ʔOre	faka3#ʔoti_kore#kore
23	Ha'aoti o Te ’Ore ’Ore ~ ’Ore ’Ore / Tangaroa Tahi	Tangaroa Tahi / Mua Tangaroa	taŋaroa_tahi
24	Mua Tangaroa / Roto o Te Tangaroa	Roto o Te Tangaroa	roto_taŋaroa

25	Roto o Te Tangaroa / Ha’aoti o Te Tangaroa	Haʔaoti o Te Tangaroa	faka3#ʔoti_taŋaroa
26	Ha’aoti o Te Tangaroa / Tane	Tane	taane
27	Rongo Nui	Rongo Nui	roŋo#nui6
28	Rongo Nui / Mauri Mate	Mauri Mate	mauri#mate2
29	Mauri Mate / Rongo Ma Uri / Motu ~ (O)Mutu	Rongo Mauri	roŋo#mauri
30	Motu ~ (O)Mutu	Mutu	mutu

List 2: HAW1a (Hawaii). Reported in Fornander (1878: 126) and reproduced in Tregear (1891: 666) (*36, 37*). We find it already in the entries of Andrews' Hawaiian vocabulary (1836) and dictionary (1865) (*38*, *39*) (see next list). It is likely that the source was the native scholar Davida Malo (1795-1853), who provided an identical list in Malo (1951 [1838]: 31-35; see also Langlas 2017: 103, Tab. 1) (*40*, *41*).

	Fornander (1878)	Phonological spelling (after Pukui and Elbert 1986 [41] and Langlas 2017)	Normalized
1	Hilo	Hilo	firo
2	Hoaka	Hoaka	so#ʔata
3	Kukahi	Kū Kahi	tuu#tahi
4	Ku-lua	Kū Lua	tuu#rua
5	Ku-kolu	Kū Kolu	tuu#toru
6	Ku-pau	Kū Pau	tuu#pau
7	Ole-ku-kahi	ʔOle Kū Kahi	kore_tuu#tahi
8	Ole-ku-lua	ʔOle Kū Lua	kore_tuu#rua
9	Ole-ku-kolu	ʔOle Kū Kolu	kore_tuu#toru
10	Ole-ku-pau	ʔOle Kū Pau	kore_tuu#pau
11	Huna	Huna	funa
12	Mohalu	Mōhalu	maharu
13	Hua	Hua	fua
14	Akua	Akua	atua
15	Hoku	Hoku	fotu/fotu
16	Mahealani	Māhealani	maseʔa2#raŋi
17	Kulu	Kulu	turu
18	Laau-ku-kahi	Lāʔau Kū Kahi	raakau_tuu#tahi
19	Laau-ku-lua	Lāʔau Kū Lua	raakau_tuu#rua
20	Laau-pau	Lāʔau Pau	raakau_pau
21	Ole-ku-kahi	ʔOle Kū Kahi	kore_tuu#tahi
22	Ole-ku-lua	ʔOle Kū Lua	kore_tuu#rua
23	Ole-pau	ʔOle Kū Pau	kore_pau
24	Kaloa-ku-kahi	Kāloa (< Kanaloa) Kū Kahi	taŋaroa_tuu#tahi
25	Kaloa-ku-lua	Kāloa (< Kanaloa) Kū Lua	taŋaroa_tuu#rua
26	Kaloa-pau	Kāloa (< Kanaloa) Pau	taŋaroa_pau
27	Kane	Kāne	taane
28	Lono	Lono	roŋo
29	Mauli	Mauli	mauri
30	Muku	Muku	mutu

List 3: HAW1b (Hawaii). This list stems from the dictionary of Andrews (1865; revised by Parker in 1922) (*39*, *42*). As noted by Williams (1928: 355) (*44*), it differs from Fornander's calendar (see HAW1a) in two aspects: (1) *Malani* is given as an alternative name of *Mahealani*; (2) the form *Kanaloa* (< **Taŋaroa*) is given as the equivalent to *Kāloa*. These are most probably conservative forms of the same names.

	Andrews (1864)	Phonological spelling (based on Pukui and Elbert 1986 [41])	Normalized
1	Hilo	Hilo	firo
2	Hoaka	Hoaka	so#ʔata
3	Kukahi	Kūkahi	tuu#tahi
4	Ku-lua	Kūlua	tuu#rua
5	Ku-kolu	Kūkolu	tuu#toru
6	Ku-pau	Kūpau	tuu#pau
7	Ole-ku-kahi	ʔOle Kū Kahi	kore_tuu#tahi
8	Ole-ku-kulua	ʔOle Kū Lua	kore_tuu#rua
9	Ole-ku-kolu	ʔOle Kū Kolu	kore_tuu#toru
10	Ole-ku-pau	ʔOle Kū Pau	kore_tuu#pau
11	Huna	Huna	funa
12	Mohalu	Mohalu	maharu
13	Hua	Hua	fua
14	Akua	Akua	atua
15	Hoku	Hoku	fotu/fotu
16	Malani	[Mālani]	maa2#raŋi
17	Kulu	Kulu	turu
18	Laau-ku-kahi	Lāʔau Kū Kahi	raakau_tuu#tahi
19	Laau-ku-lua	Lāʔau Kū Lua	raakau_tuu#rua
20	Laau-pau	Lāʔau Pau	raakau_pau
21	Ole-ku-kahi	ʔOle Kū Kahi	kore_tuu#tahi
22	Ole-ku-lua	ʔOle Kū Lua	kore_tuu#rua
23	Ole-pau	ʔOle Pau	kore_pau
24	Kanaloa-ku-kahi	Kanaloa Kū Kahi	taŋaroa_tuu#tahi
25	Kanaloa-ku-lua	Kanaloa Kū Lua	taŋaroa_tuu#rua
26	Kanaloa-pau	Kanaloa Pau	taŋaroa_pau
27	Kane	Kāne	taane
28	Lono	Lono	roŋo
29	Mauli	Mauli	mauri
30	Muku	Muku	mutu

List 4: MAO1 (Ōtaki and Ngati-Porou tribe, North Island). Reported in Best (1922: 23) (*13*) and said to be "contributed by the late Metera Ao-marere, of Otaki, who had obtained it from Mita te Tai". It is nearly identical to the incomplete list contributed by Hone Parehuia, of Ngati-Porou, as reported in Williams (1928: 350, no. 18) (*44*).

	Original	Normalized
1	Whiro	firo
2	Tirea	tireo/tirea
3	Hoata	so#ʔata
4	Oue	oue
5	Okoro	okoro
6	Tamatea	tamatea
7	Tamatea-ngana	tamatea_ŋana
8	Tamatea-aio [Tamatea-a-io?]	tamatea_aio
9	Tamatea-whakapau	tamatea_faka2#pau
10	Huna	funa
11	Ari	ʔari/ʔari
12	Hotu	fotu/fotu
13	Mawharu	maharu
14	Atua	atua
15	Ohua	fua
16	Turu	turu
17	Rakau-nui	raakau#nui4
18	Rakau-matohi	raakau#matofi
19	Takirau	tVKirau/takirau
20	Oike	oika/oike
21	Korekore	kore#kore
22	Korekore-turua	kore#kore_tuu#rua
23	Korekore whakapiri ki nga Tangaroa	kore#kore_faka4#piri_taŋaroa
24	Tangaroa-a-mua	taŋaroa_muʔa
25	Tangaroa-a-roto	taŋaroa_roto
26	Tangaroa-kiokio	taŋaroa_kiokio
27	Otane	taane
28	Orongonui	roŋo#nui6
29	Maurea [Mauri]	mauri
30	Mutu	mutu

List 5: MAO2 (Tūhoe tribe, Te Urewere, eastern North Island). In Best (1899: 103-4; 1922: 24-5) (*13, 44*). It is identical to the Tūhoe list in Williams (1928: 350, no. 16) (*44*), contributed by Tutaka Ngahau.

	Original	Normalized
1	Whiro	firo
2	Tirea	tireo/tirea
3	Hoata	so#ʔata
4	Oue	oue
5	Okoro	okoro
6	Tamatea-tutahi	tamatea_tuu#tahi
7	Tamatea-a-ngana	tamatea_ŋana
8	Tamatea-a-io	tamatea_aio
9	Tamatea-kai-ariki-whakapa	tamatea_kai#ariki_faka2#pau
10	Ari-matanui	ʔari/ʔari#matanui
11	Huna	funa
12	Mawharu	maharu
13	Maure	maure
14	Ohua	fua
15	Atua	atua
16	Hotu	fotu/fotu
17	Turu	turu
18	Rakau-nui	raakau#nui4
19	Rakau-matohi	raakau#matofi
20	Takirau	tVKirau/takirau
21	Oika	oika/oika
22	Korekore-whakatehe	kore#kore_whakatehe
23	Korekore-piri-ki-te-Tangaroa	kore#kore_piri_taŋaroa
24	Tangaroa-a-mua	taŋaroa_muʔa
25	Tangaroa-a-roto	taŋaroa_roto
26	Tangaroa-kiokio	taŋaroa_kiokio
27	Otane	taane
28	Orongonui	roŋo#nui6
29	Mauri	mauri
30	Mutuwhenua	mutu#whenua

List 6: MAO3 (New Zealand, unspecified provenance). Reported by Williams (1844) and Best (1922: 25-6) (*13*, *46*).

	Original	Normalized
1	Whiro	firo
2	Tirea	tireo/tirea
3	Hoata	so#ʔata
4	Oue	oue
5	Okoro	okoro
6	Tamatea-tutahi	tamatea_tuu#tahi
7	Tamatea-turua	tamatea_tuu#rua
8	Tamatea-tutoru	tamatea_tuu#toru
9	Tamatea-tuwha	tamatea_tuu#faa
10	Huna	funa
11	Ari	ʔari/ʔari
12	Maure	maure
13	Mawharu	maharu
14	Ohua	fua
15	Atua	atua
16	Oturu (Full moon)	turu
17	Rakau-nui	raakau#nui4
18	Rakau-matohi	raakau#matofi
19	Takirau	tVKirau/takirau
20	Oike	oika/oike
21	Korekore-tutahi	kore#kore_tuu#tahi
22	Korekore-turua	kore#kore_tuu#rua
23	Korekore-piri-ki-nga-Tangaroa	kore#kore_piri_taŋaroa
24	Tangaroa-roto	taŋaroa_roto
25	Tangaroa-kiokio	taŋaroa_kiokio
26	Tangaroa-whakapau	taŋaroa_faka2#pau
27	Otane	taane
28	Orongonui	roŋo#nui6
29	Mauri	mauri
30	Mutuwhenua / Omutu	mutu#whenua

List 7: MAO4 (Takitumu district). Given in Best (1922: 26) (*13*).

	Original	Normalized
1	Whiro	firo
2	Tirea	tireo/tirea
3	Hoata	so#ʔata
4	Oue	oue
5	Okoro	okoro
6	Tamatea-tutahi	tamatea_tuu#tahi
7	Tamatea-turua	tamatea_tuu#rua
8	Tamatea-tutoru	tamatea_tuu#toru
9	Tamatea-tuwha	tamatea_tuu#faa
10	Huna	funa
11	Ari	ʔari/ʔari
12	Mawharu	maharu
13	Atua	fua
14	Ohua	atua
15	Oturu	turu
16	Rakau-nui	raakau#nui4
17	Rakau-matohi	raakau#matofi
18	Takirau	tVKirau/takirau
19	Oike	oika/oike
20	Korekore-tutahi	kore#kore_tuu#tahi
21	Korekore-turua	kore#kore_tuu#rua
22	Korekore-tutoru	kore#kore_tuu#toru
23	Tangaroa-a-mua	taŋaroa_muʔa
24	Tangaroa-roto	taŋaroa_roto
25	Tangaroa-kiokio	taŋaroa_kiokio
26	Kiokio	kiokio
27	Otane	taane
28	Orongonui	roŋo#nui6
29	Mauri	mauri
30	Mutuwhenua / Omutu	mutu#whenua

List 8: MAO5 (New Zealand, unspecified provenance). Best (1922: 26-7; see also Williams 1928: 351, no. 20) described this list as “culled from the late Mr. John White’s papers” (*13*, *44*).

	Original	Normalized
1	Whiro	firo
2	Tirea	tireo/tirea
3	Hoata	so#ʔata
4	Oue	oue
5	Okoro	okoro
6	Tamatea-kai-ariki	tamatea_kai#ariki
7	Tamatea-turua	tamatea_tuu#rua
8	Tamatea	tamatea
9	Tamatea whakapau	tamatea_faka2#pau
10	Hune [? Huna]	funa
11	Ari	ʔari/ʔari
12	Maure	maure
13	Mawharu	maharu
14	Atua	atua
15	Hotu	fotu/fotu
16	Oturu	turu
17	Rakau-nui	raakau#nui4
18	Rakau-matohi	raakau#matofi
19	Takirau	tVKirau/takirau
20	Oike	oika/oike
21	Korekore-tutahi	kore#kore_tuu#tahi
22	Korekore-turua	kore#kore_tuu#rua
23	Korekore-piri-ki-nga-Tangaroa	kore#kore_piri_taŋaroa
24	Tangaroa-a-mua	taŋaroa_muʔa
25	Tangaroa-a-roto	taŋaroa_roto
26	Kiokio	kiokio
27	Otane	taane
28	Orongonui	roŋo#nui6
29	Orongomauri	roŋo#mauri
30	Mutuwhenua	mutu#whenua

List 9: MAO6 (Ngāti Kahungunu). Best (1922: 28-9; also Williams 1928: 351, no. 21) (*13*, *44*).

	Original	Normalized
1	Whiro	firo
2	Tirea	tireo/tirea
3	Hoata	so#ʔata
4	Ouenuku	oue#nuku
5	Okoro	okoro
6	Tamatea-ngana	tamatea_ŋana
7	Tamatea-kai-ariki	tamatea_kai#ariki
8	Huna	funa
9	Ari-roa	ʔari/ʔari#roa
10	Maure	maure
11	Mawharu	maharu
12	Ohua	fua
13	Hotu	fotu/fotu
14	Atua	atua
15	Turu	turu
16	Rakau-nui	raakau#nui4
17	Rakau-matohi	raakau#matofi
18	Takirau	tVKirau/takirau
19	Oike	oika/oike
20	Korekore-te-whiwhia	kore#kore_whiwhia
21	Korekore-te-rawea	kore#kore_rawea
22	Korekore-hahani	kore#kore_hahani
23	Tangaroa-a(-)mua	taŋaroa_muʔa
24	Tangaroa-a(-)roto	taŋaroa_roto
25	Tangaroa-kiokio	taŋaroa_kiokio
26	Otane	taane
27	Orongonui	roŋo#nui6
28	Mauri	mauri
29	Omutu	mutu
30	Mutuwhenua	mutu#whenua

List 10: MAO7 (Okirihau, i.e., Tieke Kāinga, in North Island). Best (1922: 29-30) (*13*) reports this list as follows: "contributed by Wi Kingi, of Okirihau, in 1849 appears in Mr. White's MS. matter. It has the peculiarity of commencing with the disappearing of the old moon. It resembles a list given by the Rev. R. Taylor, and contains names not known on the eastern side of the Island. (...) The rest of the names agree pretty well with east-coast lists, save that Oike becomes Ohika, Rongomai replaces Orongonui, and the 15th and 25th nights both appear as Kiokio. Only twenty-nine names are given." This description matches the (complete) list given as no. 6 ("Taylor") in Williams (1928: 346) (*44*), except that in the latter work "Takatakaputei" and "Maweti" are given where Best writes "Takatakaputea" and "Mawete", respectively. Misprints may be involved. It is also very similar to the odd list of 33 nights given in Williams (1928: no. 8) (*44*).

	Original	Normalized
1	Nonihape	nonihape
2	Takataka-putea (Takatakaputei)	takatakapuutea
3	Whitikiraua	whitikiraua
4	Ohoata	so#ʔata
5	Ouenuku	oue#nuku
6	Mawete (Maweti)	mawete
7	Tutahi	tuu#tahi
8	Otama	otama
9	Pa	pa
10	Ari	ʔari/ʔari
11	Hune	funa
12	Mawaru	maharu
13	Hua	fua
14	Atua	atua
15	Kiokio	kiokio
16	Rakau nui	raakau#nui4
17	Rakau matohi	raakau#matofi
18	Takirau	tVKirau/takirau
19	Ohika	oika/oika
20	Korekore	kore#kore
21	Korekore tutahi	kore#kore_tuu#tahi
22	Korekore wakapou	kore#kore_faka2#pau
23	Tangaroa a mua	taŋaroa_muʔa
24	Tangaroa a roto	taŋaroa_roto
25	Kiokio	kiokio
26	Otane	taane
27	Rongomai	roŋo#mai
28	Mouri	mauri
29	Omutu	mutu

List 11: MAO8 (New Zealand, unspecified location). Reported by Best (1922: 30-31; also Williams 1928: 353, no. 25) (*13*, *44*) as “collected by the late Judge Fenton” from a “Native informant”. The latter refers to Fenton (1885: 131), which contains some typographical errors (e.g., Oue appears as “one” and Okoro as “okou”) (*47*).

	Original	Normalized
1	Whiro	firo
2	Tirea	tireo/tirea
3	Hoata	so#ʔata
4	Oue	oue
5	Okoro	okoro
6	Tamatea-ariki	tamatea_ariki
7	Tamatea-ananga	tamatea_ŋana
8	Tamatea-aio	tamatea_aio
9	Tamatea-whakapau	tamatea_faka2#pau
10	Huna	funa
11	Ari-roa	ʔari/ʔari#roa
12	Mawharu	maharu
13	Maurea	maure
14	Atua-whakahaehae	atua#whakahaehae
15	Turu	turu
16	Rakau-nui	raakau#nui4
17	Rakau-matohi	raakau#matofi
18	Takirau	tVKirau/takirau
19	Oika	oika/oika
20	Korekore	kore#kore
21	Korekore-turua	kore#kore_tuu#rua
22	Korekore-piri ki nga Tangaroa	kore#kore_piri_taŋaroa
23	Tangaroa-amua	taŋaroa_muʔa
24	Tangaroa-aroto	taŋaroa_roto
25	Tangaroa-kiokio	taŋaroa_kiokio
26	Otane	taane
27	Orongonui	roŋo#nui6
28	Mauri	mauri
29	Omutu	mutu
30	Mutuwhenua	mutu#whenua

List 12: MAO_AWA (Ngati-Awa tribe, eastern Bay of Plenty Region of New Zealand). Best (1899: 103-4) (*45*).

	Original	Normalized
1	Whiro	firo
2	Tirea	tireo/tirea
3	Hoata	so#ʔata
4	Ouenuku	oue#nuku
5	Okoro	okoro
6	Tamatea-ngana	tamatea_ŋana
7	Tamatea-kani	tamatea_kani
8	Tamatea-kai-ariki	tamatea_kai#ariki
9	Tamatea-aio	tamatea_aio
10	Tamatea-whakapa	tamatea_faka2#pau
11	Huna	funa
12	Ari	ʔari/ʔari
13	Maure	maure
14	Mawharu	maharu
15	Ohua	fua
16	Hotu	fotu/fotu
17	Atua	atua
18	Turu	turu
19	Rakau-nui	raakau#nui4
20	Rakau-matohi	raakau#matofi
21	Takirau	tVKirau/takirau
22	Oike	oika/oike
23	Korekore-piri-ki-nga-Tangaroa	kore#kore_piri_taŋaroa
24	Tangaroa-a-mua	taŋaroa_muʔa
25	Tangaroa-a-roto	taŋaroa_roto
26	Tangaroa-kiokio	taŋaroa_kiokio
27	O-tane	taane
28	O-rongo-nui	roŋo#nui6
29	Mauri	mauri
30	Mutuwhenua	mutu#whenua

List 13: MAO_WAI (Waikato district, New Zealand). Reported in Firth (1929: 63 and n. 1), after a list sent by W. Baucke, from Otorohanga (48).

	Original	Normalized
1	Atarau, also Pewa	pewa
2	Ahoroa, also Tirea	tireo/tirea
3	Aurei	aurei
4	Oue, also Ue	oue
5	Akoro, also Okoro	okoro
6	Ananga, also Tamatea-tutahi	tamatea_tuu#tahi
7	Ahotu, also Tamatea-turua	tamatea_tuu#rua
8	Aio, also Tamatea-to-toru	tamatea_tuu#toru
9	Kai-ariki, also Tamatea-tuwha	tamatea_tuu#faa
10	Hune, also Ngahuru (i.e., ‘Ten’)	funa
11	Ari	ʔari/ʔari
12	Maure	maure
13	Mawharu	maharu
14	Ohua	fua
15	Atua-mate-o-hotu	atua#mate1#fotu/fotu
16	Oturu	turu
17	Rakau-nui	raakau#nui4
18	Rakau-matohi	raakau#matofi
19	Takirau	tVKirau/takirau
20	Oika	oika/oika
21	Korekore	kore#kore
22	Korekore-ngana	kore#kore_ŋana
23	Korekore-piri	kore#kore_piri
24	Tangaroa-mua	taŋaroa_muʔa
25	Tangaroa-roto	taŋaroa_roto
26	Kiokio	kiokio
27	Otane	taane
28	Orongo-nui	roŋo#nui6
29	Mauri	mauri
30	Omutu-mutu whenua	mutu#mutu#whenua

List 14: MGV (Mangareva). The same list appears in Tregear's (1899: 46) Mangarevan dictionary and Janeau's (1908) grammar (*49*, *50*). Tregear's version shows minor differences. First, he gave the Marquesan-like form *Ma*[*ʔ*]*eama* instead of Mangarevan *Ma*[*ʔ*]*ema*. Second, there are two misprints: "Ohuma" for *O Huna, and "Ohiru" for *O Turu. Neither author notates the glottal stop *ʔ* (< **h*) and both have "Kore-tai" where we might expect **Korekore-taʔi* 'Korekore One.' Hiroa, who discussed the list at length (Hiroa 1938: 403-411) (*7*) correctly noted that "Ohoata" and "Tu-nui" should be at the beginning of the month. We used a version of the list that reflects this insight (even though our computational analysis would have been able to recognize the relative position of these nights regardless; see below).

	Tregear (1899)	Janeau (1908)	Normalization
29	Ohoata	Ohoata	so#ʔata
30	Tu-nui	Tunui	tuu#nui1
1	Ma[ʔ]eama-ta[ʔ]i	Ma[ʔ]ema ta(h)i	maseʔa/maseʔa#maa_tahi
2	Ma[ʔ]eama-rua	Ma[ʔ]ema rua	maseʔa/maseʔa#maa_rua
3	Ma[ʔ]eama-toru	Ma[ʔ]ema toru	maseʔa/maseʔa#maa_toru
4	Ma[ʔ]eama-riro	Ma[ʔ]ema riro	maseʔa/maseʔa#maa_riro
5	Kore-ta[ʔ]i	Kore ta(h)i	kore_tahi
6	Korekore-rua	Korekore rua	kore#kore_rua
7	Korekore-toru	Korekore toru	kore#kore_toru
8	Korekore-kaha	Korekore kaha	kore#kore_kaha
9	Oari	Oari	ʔari/ʔari
10	Ohuma [Ohuna]	Ohuna	funa
11	Omaharu	Omawaru [Omaharu]	maharu
12	Ohua	Ohua	fua
13	Oetua	Oetua	atua
14	Ohotu (full moon)	Ohotu (full moon)	fotu/fotu
15	Omaure	Oma(h)ure	maure
16	Ohiru [Oturu]	Oturu	turu
17	Orakau	Orakau	raakau
18	Omotohi	Omotohi, Omotahi	matofi
19	Korekore-ta[ʔ]i	Kore ta(h)i	kore_tahi
20	Korekore-rua	Korekore rua	kore#kore_rua
21	Korekore-toru	Korekore toru	kore#kore_toru
22	Korekore-riro	Korekore riro	kore#kore_riro
23	Vehi-tahi	Vehi ta(h)i	vehi_tahi
24	Vehi-rua	Vehi rua	vehi_rua
25	Vehi-toru	Vehi toru	vehi_toru
26	Vehi-riro	Vehi riro	vehi_riro
27	Otane	Otane	taane
28	Omouri	Omouri	mauri

List 15: MORI (Moriori / Chatham Islands). Reported by Shand (1898: 88) and presented also in Tregear (1891: 666, 1889: 77) and Best (1922: 32, no. 9) (*13*, *37, 51, 52*). Williams (1928: 355, no. 31) more appropriately places Shand's "Omuti" (a misprint for "Omutu") at the end of the list (*44*). We used this last version.

	Original	Normalized
1	Owhiro	firo
2	Otere [O Tirea]	tireo/tere
3	Ohewhata [O Hoata]	so#ʔata
4	Oua	oue
5	Okoro	okoro
6	Tamate-tutahi	tamatea_tuu#tahi
7	Tamate-turua	tamatea_tuu#rua
8	Tamate-nui	tamatea_nui2
9	Tamate-hokopa[u?]	tamatea_faka2#pau
10	Ohuna	funa
11	Howaru [Oari??]	ʔari/howaru
12	Hua	fua
13	Mawharu	maharu
14	Outua	atua
15	Ohotu	fotu/fotu
16	Maure	maure
17	Oturu	turu
18	Rakau-nui	raakau#nui4
19	Rakau-motohe	raakau#matofi
20	Takirau	tVKirau/takirau
21	Oika	oika/oika
22	Korekore-tutahi	kore#kore_tuu#tahi
23	Korekore-turua	kore#kore_tuu#rua
24	Korekore-hokopau	kore#kore_faka2#pau
25	Tangaro-amua	taŋaroa_muʔa
26	Tangaro-aroto	taŋaroa_roto
27	Tangaro-kikio	taŋaroa_kiokio
28	Otane	taane
29	Orongonui	roŋo#nui6
30	Orongomori [Orongomauri]	roŋo#mauri
31	Omutu	mutu

List 16: MRQ1 (Unspecified location in southeastern Marquesas). This list was first reported in Fornander (1878: 126) with 'Hawaiianizing' orthography (e.g., *k* for Marquesan *t*) (*36*). Tregear (1891: 666) amended it but failed to normalize it in full (*37*). In any event, once restored, the list is remarkably similar to other Marquesan versions. There are also obvious issues with the transcription. For Hotu-manae = Hotu-mauʔe, see next list.

	Original publication	Restoration (This paper)	Normalized
1	Tu-nui	Tū-nui	tuu#nui1
2	Tu-hawa	Tū-hava	tuu#hava
3	Hoata	Hoata	so#ʔata
4	Maheamatahi	Mahea ma tahi	maseʔa/maseʔa#maa_tahi
5	Maheamawaena	Mahea ma va[v]ena	maseʔa/maseʔa#maa_waheŋa
6	Koekoe-tahi	Ko[ʔ]eko[ʔ]e tahi	kore#kore_tahi
7	Koekoe-waena	Ko[ʔ]eko[ʔ]e va[v]ena	kore#kore_waheŋa
8	Poipoi-haapao	Poipoi ha[ʔ]apao	poipoi_faka2#pau
9	Huna	Huna	funa
10	A'i	Aʔi	ʔari/ʔari
11	Huka	Huka, Huta, or Hu<n>a(?)	huka
12	Meha'u	Mehaʔu	maharu
13	Hua	Hua	fua
14	Atua	Atua	atua
15	Hutu-nui	Hotu-nui	fotu/fotu#nui3
16	Hutu-manae	Hotu-manae [maure]	fotu/fotu#maure
17	Tu'u	Tuʔu	turu
18	Aniwa	ʔAniva	ʔaniwa
19	Ma tahi	Matohi	matofi
20	Kaau	[ʔā]kau	raakau
21	Kaekae-tahi	K<o>[ʔ]ek<o>[ʔ]e tahi	kore#kore_tahi
22	Waena	[Koʔekoʔe] va[v]ena	kore#kore_waheŋa
23	Haa pao	[Koʔekoʔe] ha[ʔ]apao	kore#kore_faka2#pau
24	Hanao tahi	<T>ana[ʔ]o[a] tahi	taŋaroa_tahi
25	Wawena	[Tanaʔoa] va[v]ena	taŋaroa_waheŋa
26	Haapaa	[Tanaʔoa] ha[ʔ]apa<o>	taŋaroa_faka2#pau
27	Puhiwa	Pū-hiva	puu#hiva
28	Tane	Tāne	taane
29	Ona-nui [ʔono nui]	ʔOno-nui	roŋo#nui6
30	Onamate	ʔOno-mate	roŋo#mate2

List 17: MRQ2 (Southeast Marquesas, unspecified location). In Audran (1929) (53*53*).

	Original publication	Analytical transcription	Normalized
1	Tunui	Tū-nui	tuu#nui1
2	Tuhava	Tū-hava	tuu#hava
3	Tuhakaoata	Tū-haka[h]oata	tuu#faka1#so#ʔata
4	Maheamatahi	Maheama-tahi	maseʔa/maseʔa#maa_tahi
5	Maheama-ivavana	Maheama-i-vavena	maseʔa/maseʔa#maa_waheŋa
6	Maheama-haapao	Maheama-ha[ʔ]apao	maseʔa/maseʔa#maa_faka2#pau
7	Ko[ʔ]ekoe-tahi	Koʔekoʔe-tahi	kore#kore_tahi
8	Koekoe-ivavena	Koʔekoʔe-i-vavena	kore#kore_waheŋa
9	Koekoe-haapao	Koʔekoʔe-haʔapao	kore#kore_faka2#pau
10	Ai	Aʔi	ʔari/ʔari
11	Huna	Huna	funa
12	Maheau	Maheaʔu	maharu
13	Ohua	O Hua	fua
14	Oatua	O Atua	atua
15	Ohotonui	O Hotu-nui	fotu/fotu#nui3
16	Ohotumaie	O Hotu-mauʔe	fotu/fotu#maure
17	O tu[ʔ]u	O Tuʔu	turu
18	Oaniva	O ʔAniva	ʔaniwa
19	Oakau	O ʔAakau	raakau
20	Onetohi	O Metohi	matofi
21	Koekoe-tahi	Koʔekoʔe-tahi	kore#kore_tahi
22	Koekoe-vaveka	Koʔekoʔe-vavena	kore#kore_waheŋa
23	Koekoe-haapao	Koʔekoʔe-haʔapao	kore#kore_faka2#pau
24	Tuhiva	Tū-hiva	tuu#hiva
25	Oatiati	O Atiati	atiati
26	Ovaka	O Vaka	vaka
27	Ovehi	O Vehi	vehi
28	O Tane	O Tāne	taane
29	Ono nui	ʔOno-nui	roŋo#nui6
30	Onomate	ʔOno-mate	roŋo#mate2

List 18: MRQ3 (Northwest Marquesas, unspecified location). In Audran (1929) (*53*). "Hatanui" (10th position) is surely a repetition of "Ohotonui", i.e., Hotu-nui (16th position). If we assume this is a mistake and remove Hatanui, the result is a regular list of 30 nights.

	Original publication	Analytical transcription	Normalized
1	Tuhava	Tū-hava	tuu#hava
2	Tunu [Tu-nui]	Tū-nui	tuu#nui1
3	Tuhakao[a]ta	Tū-haka<h>oata	tuu#faka1#so#ʔata
4	Maheamatahi	Maheama-tahi	maseʔa/maseʔa#maa_tahi
5	Maeheama-ua	Maheama-ʔua	maseʔa/maseʔa#maa_rua
6	Maheama-tou	Maheama-toʔu	maseʔa/maseʔa#maa_toru
7	Koekoe-tahi	Koʔekoʔe-tahi	kore#kore_tahi
8	Koekoe-ua	Koʔekoʔe-ʔua	kore#kore_rua
9	Koekoe-tou	Koʔekoʔe-toʔu	kore#kore_toru
10	Hatanui	Hotu-nui [repeated]	-
11	Ai	Aʔi	ʔari/ʔari
12	Hua	Hua	fua
13	Mahari	Maharu	maharu
14	Aivaku	Aivaku	aivaku
15	Atua	Atua	atua
16	Ohotunui	O Hotu-nui	fotu/fotu#nui3
17	Honu maue	O Honu-mauʔe	fotu/fonu#maure
18	Tuu	O Tuʔu	turu
19	Akau	O ʔĀkau	raakau
20	Motohi	Motohi	matofi
21	Tohiau	Tohiʔau	tVKirau/tohiʔau
22	Taukume	Taukume	taukume
23	Kumea	Kumea	kumea
24	Tea	Tea	tea3
25	Takaoatu-tahi	Takaʔoa-tūtahi	taŋaroa_tuu#tahi
26	Takaoatu-vavena	Takaʔoa-tū-vave<k>a	taŋaroa_tuu#waheŋa
27	Takaoatu-haapao	Takaʔoa-tū-haʔapao	taŋaroa_tuu#faka2#pau
28	Evehi	E Vehi	vehi
29	Tane	Tāne	taane
30	Mouiheo [Mouikeo]	Mouʔi-keʔo	mauri#kero
31	Okomate	ʔOko-mate	roŋo#mate2

List 19: MRQ4 (Marquesas, unspecified location). Reported in Handy (1932: 348-9), after a manuscript ("MS") by Dordillon (*54, 55*). It is very similar to MRQ3.

	Original publication	Analytical transcription	Normalized
1	O Tunui	Tū-nui	tuu#nui1
2	O Tuhava	Tū-hava	tuu#hava
3	O Tuhaka	Tū-haka<hoata>	tuu#faka1#so#ʔata
4	O Maheama tuatahi	O Maheama-tuatahi	maseʔa/maseʔa#maa tuu#tahi
5	O Maheama vaveka	O Maheama-vaveka	maseʔa/maseʔa#maa waheŋa
6	O Maheama hakapao	O Maheama-hakapao	maseʔa/maseʔa#maa faka2#pau
7	O Koekoe tuatahi	O Koʔekoʔe-tuatahi	kore#kore tuu#tahi
8	O Koekoe vaveka	O Koʔekoʔe-vaveka	kore#kore_waheŋa
9	O Koekoe hakapao	O Koʔekoʔe-hakapao	kore#kore faka2#pau
10	O Ai	O Aʔi	ʔari/ʔari
11	O Huna	O Huna	funa
12	O Mahau	O Maha[ʔ]u	maharu
13	O Ua	O <H>ua	fua
14	O Atua	O Atua	atua
15	O Tunui	O <H>otu-nui	fotu/fotu#nui3
16	O Mahuto	O Mahu<re>?	maure
17	O Tuu	O Tuʔu	turu
18	O Akau	O ʔĀkau	raakau
19	O Motohi	O Motohi	matofi
20	O Tohiau	O Tohiʔau	tVKirau/tohiʔau
21	O Taukume	O Taukume	taukume
22	O Kumea	O Kumea	kumea
23	O Eea	Tea	tea3
24	O Takaoa tutahi	O Takaʔoa-tūtahi	taŋaroa tuu#tahi
25	O Takaoa vaveka	O Takaʔoa-vaveka	taŋaroa tuu#waheŋa
26	O Takaoa hakapao	O Takaʔoa-hakapao	taŋaroa tuu#faka2#pau
27	O Vehi	O Vehi	vehi
28	O Tane	O Tāne	taane
29	O Mouikeo	O Mouʔi-keʔo	mauri#kero
30	O Oko mate	O ʔOko-mate	roŋo#mate2

List 20: MRQ5 (Unspecified location). Reported in Handy (1932: 348-9), after the dictionary of Dordillon (1931) (*54*, *55*). Oddly, it contains forms written both in the northern and southern dialects of Marquesan. We compared Handy's transcription against Dordillon's dictionary entries, and this resolved the interpretation of difficult names. A very similar list seems to be reported in Dordillon (1931: 328-329), but the dictionary contains data on only 27 nights and is therefore insufficient to reconstruct it in full: "Noms des jours des lunes : Okomate, Tuhava, Hoata, Maheama haápao, Koekoe tutahi, Koekoe vaveka, Koekore haápao, Aí, Huna, Mahaó[,] Hua, Honu nui, Honu maákau, Tuu, Ákau, Motohi, Aniva, Takava[,?] atiati, Takaoa tukete, Takaoakau, Vehi, Tane, Mouikeo (Muoitutu, Mauihahaka, Tetiaka, compris sous un seul jour qui s'apelle *Tu*)" (*55*).

	Original publication	Analytical transcription	Normalized
1	O Tunui	Tū-nui	tuu#nui1
2	O Tuhava	Tū-hava	tuu#hava
3	Tuhaahoata<br>(Tuhaá hoata / Tuhaka hoata)	Tū-haʔahoata	tuu#faka1#so#ʔata
4	Maheama tahi	Maheama-tahi	maseʔa/maseʔa#maa_tahi
5	Maheama vaveka	Maheama-vaveka	maseʔa/maseʔa#maa_waheŋa
6	Maheama hakapao	Maheama-hakapao	maseʔa/maseʔa#maa_faka2#pau
7	Ko[ʔ]eko[ʔ]e tutahi	Koʔekoʔe-tūtahi	kore#kore_tuu#tahi
8	Ko[ʔ]eko[ʔ]e vavena	Koʔekoʔe-vaveka	kore#kore_waheŋa
9	Ko[ʔ]eko[ʔ]e hakapao	Koʔekoʔe-hakapao	kore#kore_faka2#pau
10	O A[ʔ]i	O Aʔi	ʔari/ʔari
11	O Huna	O Huna	funa
12	Mahao [Mahaʔu]	Mahaʔu	maharu
13	Hua	Hua	fua
14	Atua	Atua	atua
15	Honu nui	Honu-nui	fotu/fonu#nui3
16	Honu maakau<br>(Honu ma ákau)	Honu-ma-ʔĀkau	fotu/fotu_raakau
17	Tu[ʔ]u	Tuʔu	turu
18	Aniva	ʔAniva	ʔaniwa
19	[ʔ]Akau	ʔĀkau	raakau
20	Tohi[ʔ]au	Tohiʔau	tVKirau/tohiʔau
21	Ko[ʔ]eko[ʔ]e ahi	Koʔekoʔe-tahi	kore#kore_tahi
22	Ko[ʔ]eko[ʔ]e vavena	Koʔekoʔe-vaveka	kore#kore_waheŋa
23	Ko[ʔ]eko[ʔ]e haapao	Koʔekoʔe-hakapao	kore#kore_faka2#pau
24	Tuhiva	Tū-hiva	tuu2#hiva
25	(Takaoa) atiati	(Takaʔoa) Atiati	atiati
26	Vaka	Vaka	vaka
27	Vehi	Vehi	vehi
28	Tane	Tāne	taane
29	[ʔ]Ononui	ʔOno-nui	roŋo#nui6
30	[ʔ]Onomate(?)	ʔOno-mate	roŋo#mate2

List 21: MRQ6 (Hiva ’Oa). Reported in Handy (1932: 348-9) (*54*).

	Original	Normalized
1	O Tunui	tuu#nui1
2	O Tuhava	tuu#hava
3	Tuha[k]ahoata	tuu#faka1#so#ʔata
4	Maheama tahi	maseʔa/maseʔa#maa_tahi
5	Maheama vavena	maseʔa/maseʔa#maa_waheŋa
6	Maheama hakapao	maseʔa/maseʔa#maa_faka2#pau
7	Ko[ʔ]eko[ʔ]e tahi	kore#kore_tahi
8	Ko[ʔ]eko[ʔ]e vavena	kore#kore_waheŋa
9	Ko[ʔ]eko[ʔ]e haapao	kore#kore_faka2#pau
10	O A[ʔ]i	ʔari/ʔari
11	O Huna	funa
12	Meha[ʔ]u	maharu
13	Ua	fua
14	Atua	atua
15	Hotunui	fotu/fotu#nui3
16	Hotu mau[ʔ]e / Mau[ʔ]e	fotu/fotu#maure
17	Tu[ʔ]u	turu
18	Aniva	ʔaniwa
19	Metohi	matofi
20	[ʔ]Akau	raakau
21	Ko[ʔ]eko[ʔ]e tahi	kore#kore_tahi
22	Ko[ʔ]eko[ʔ]e vavena	kore#kore_waheŋa
23	Ko[ʔ]eko[ʔ]e haapao	kore#kore_faka2#pau
24	Tana[ʔ]oa tahi	taŋaroa_tahi
25	Tana[ʔ]oa vavena	taŋaroa_waheŋa
26	O Tana[ʔ]oa haapao	taŋaroa_faka2#pau
27	Tane	taane
28	Mou[ʔ]i	mauri
29	[ʔ]Ononui	roŋo#nui6
30	[ʔ]Onomate	roŋo#mate2

List 22: MRQ7 (Fatu Hiva). Reported in Handy (1932: 348-9) (*54*).

	Original publication	Normalized
1	O Tunui	tuu#nui1
2	O Tuhava	tuu#hava
3	Hoata	so#ʔata
4	Maheama tahi	maseʔa/maseʔa#maa_tahi
5	Maheama vaveka	maseʔa/maseʔa#maa_waheŋa
6	Maheama hakapao	maseʔa/maseʔa#maa_faka2#pau
7	Ko[ʔ]eko[ʔ]e tahi	kore#kore_tahi
8	Ko[ʔ]eko[ʔ]e vavena	kore#kore_waheŋa
9	Ko[ʔ]eko[ʔ]e hakapao	kore#kore_faka2#pau
10	O A[ʔ]i	ʔari/ʔari
11	O Huna	funa
12	Meha[ʔ]u	maharu
13	Ua	fua
14	Atua	atua
15	Hotunui	fotu/fotu#nui3
16	Hotu [ʔ]akau	fotu/fotu_raakau
17	Tu[ʔ]u	turu
18	Aniva	ʔaniwa
19	Metohi	matofi
20	[ʔ]Akau	raakau
21	Ko[ʔ]eko[ʔ]e tahi	kore#kore_tahi
22	Ko[ʔ]eko[ʔ]e vavena	kore#kore_waheŋa
23	Ko[ʔ]eko[ʔ]e haapao	kore#kore_faka2#pau
24	Tuhiva	tuu2#hiva
25	Tana[ʔ]oa tahi	taŋaroa_tahi
26	Tana[ʔ]oa vavena	taŋaroa_waheŋa
27	Tana[ʔ]oa haapao	taŋaroa_faka2#pau
28	Tane	taane
29	[ʔ]Ononui	roŋo#nui6
30	[ʔ]Onomate	roŋo#mate2

List 23: MRQ8 (Ua Huka). Reported in Handy (1932: 348-9) (*54*). The odd and unparalleled 'Tana hau' (27th and 28th nights) is likely a misprint for southern Marquesan 'Tana[ʔ]oa', the name of a series of nights usually found around the positions 24th-27th. 'Tana hau' is not found elsewhere. Still, we treated it as a night without cognates to avoid making strong assumptions.

	Original	Normalized
1	Tu	tuu
2	Maheama tutahi	maseʔa/maseʔa#maa_tuu#tahi
3	Maheama vavena	maseʔa/maseʔa#maa_waheŋa
4	Maheama tuhakapau	maseʔa/maseʔa#maa_tuu#faka2#pau
5	Ko[ʔ]eko[ʔ]e tutahi	kore#kore_tuu#tahi
6	Ko[ʔ]eko[ʔ]e tuvavena	kore#kore_tuu#waheŋa
7	Ko[ʔ]eko[ʔ]e tuhakapau	kore#kore_tuu#faka2#pau
8	Hai [Aʔi]	ʔari/ʔari
9	[ʔ]Una	funa
10	Maha[ʔ]u	maharu
11	Hua	fua
12	Tua [Atua]	atua
13	ʔOtu-nui	fotu/fotu#nui3
14	Otu-mauii	fotu/fotu#maure
15	[ʔ]otu	fotu/fotu
16	Neva	ʔaniwa
17	Metohe	matofi
18	Hakahau [Akau?]	raakau
19	Ko[ʔ]eko[ʔ]e tahi	kore#kore_tahi
20	Ko[ʔ]eko[ʔ]e vavena	kore#kore_waheŋa
21	Ko[ʔ]eko[ʔ]e pau	kore#kore_pau
22	Vehe [Vehi]	vehi
23	Tane	taane
24	Hee hee ia	heeheeia
25	Taukame [Taukume]	taukume
26	Ku mea	kumea
27	Tana hau vaka	tanahau_vaka
28	Tana hau vehi	tanahau_vehi
29	Nunui [ʔono-nui?]	roŋo#nui6
30	Nu mata [ʔono-mate?]	roŋo#mate2

List 24: NCOOK_MANI (Manihiki). After Hiroa (1932: 221, Tab. 17) (*16*). Some peculiar aspects of this list must be interpreted in conjunction with the similar one from the neighboring atoll of Rakahanga (see NCOOK_RAKA below). Thus, the form *Tūtahi* 'self-standing, solitary' (as seen in Rakahanga and other calendars) was clearly reinterpreted as *Tuatahi* 'first' at Manihiki. This meant the replacement of the morpheme *Tū* with the ordinalizing prefix *tua-*, which then spread to the name of the first night. Thus, Manihiki has *Tiatua* = **Te a Tua* vs. Rakahanga *Te atu* = *Te a Tū* (?). For our study, we normalized it as "tuu." It is also odd that Mutuŋa, literally 'Ending,' appears in this list as the first rather than last night of the list.

	Original (manuscript forms)	Interpretation	Normalized
1	Te Mutunga	Te Mutuŋa	mutu#ŋa4
2	Tiatua	Te a Tua (< *Tū)	tuu
3	Tuatahi	Tuatahi (*Tūtahi)	tuu#tahi
4	Tuatahi-rua	Tuatahi (*Tūtahi) rua	tuu#rua
5	Tuatahi-toru	Tuatahi (*Tūtahi) toru	tuu#toru
6	Tamatea-tuatahi	Tamatea tuatahi	tamatea_tuu#tahi
7	Tamatea-turua	Tamatea tūrua	tamatea_tuu#rua
8	Tamatea-tuturo	Tamatea tūtoru	tamatea_tuu#toru
9	Tehari	Te (ʔ)Ari	ʔari/ʔari
10	Korekore	Korekore	kore#kore
11	Tehune [Hune]	Te Hune	funa
12	Tiohua [Hua]	Te o Hua	fua
13	Temahari	Te Maʔaru	maharu
14	Tihotuamua [Otua-mua]	Te Otua-mua	atua_muʔa
15	Tihotuamuri [Otua-muri]	Te Otua-amuri	atua_muri
16	Tihowhotu [Whotu]	Te whotu	fotu/fotu
17	Te marangi	Te maaraŋi	maa2#raŋi
18	Whetau-maro	Whetau-maro	hetaumaro
19	Rakau-tahi	Rākau tahi	raakau_tahi
20	Rakaurua	Rākau rua	raakau_rua
21	Rakau-toru	Rākau toru	raakau_toru
22	Korekore-tahi	Korekore tahi	kore#kore_tahi
23	Korekore-rua	Korekore rua	kore#kore_rua
24	Korekore-toru	Korekore toru	kore#kore_toru
25	Tangaroa-tahi	Taŋaroa tahi	taŋaroa_tahi
26	Tangaroa-rua	Taŋaroa rua	taŋaroa_rua
27	Tangaroa-toru	Taŋaroa toru	taŋaroa_toru
28	Te Tane	Te Tāne	taane
29	Te Rongonui	Te Roŋo-nui	roŋo#nui6
30	Te Mauri	Mauri	mauri

List 25: NCOOK_RAKA (Rakahanga). Hiroa (1932: 218, Tab. 16): "obtained from the writings of an old man named Haumata-tua, who recorded them in a ledger now in the possession of Aporo of Rakahanga. The names as spelled in this manuscript are given in table 16. The actual night names without the introductory particle *ko* or the definite articles *te*, *ti*, or *ta*, with the pronounced h and wh inserted, are given in parentheses" (*16*). Some odd aspects of this list have been interpreted considering the similar list from the neighboring atoll of Manihiki (see NCOOK_MANI above). For instance, given its position after Hune and before Maharu, "Ohau" must be corrected to "O Hua."

	Original (manuscript forms)	Only names (according to Hiroa 1932)	Interpretation (This paper)	Normalized
1	Te atu	Atu	Te a Tū	tuu
2	Te turai	Tutahi	Te Tū(-)tahi	tuu#tahi
3	Te turoto	Turoto	Te Tūroto	tuu#roto
4	Tamatea tutai	Tamatea tutahi	Tamatea tūtaʔi	tamatea_tuu#tahi
5	Tamatea turoto	Tamatea turoto	Tamatea tūroto	tamatea_tuu#roto
6	Tamatea akaoti	Tamatea whakaoti	Tamatea ʔakaoti	tamatea_faka3#ʔoti
7	Ko Tiooata	Hoata	Ko te ʔoata	so#ʔata
8	Ko Tiari	Ari	Ko te Ari	ʔari/ʔari
9	Kote korekore tai	Korekore tahi	Ko te Korekore taʔi	kore#kore_tahi
10	Ko Uune	Hune	Ko ʔUne	funa
11	Ko Tioau	Ohau	Ko te *ʔua	fua
12	Ko Tamaaru	Maharu	Ko te Maʔaru	maharu
13	Ko Tiotua mua	Otua mua	Ko te Otua mua	atua_muʔa
14	Ko Tiotua muri	Otua muri	Ko te Otua muri	atua_muri
15	Ko Tiotu	Hotu	Ko te ʔOtu	fotu/fotu
16	Ko marangi	Marangi	Ko Maaraŋi	maa2#raŋi
17	To etau maro	Whetau-maro	To Hetau-maro	hetaumaro
18	Rakau tai	Rakau tahi	Rākau taʔi	raakau_tahi
19	Rakau roto	Rakau roto	Rākau roto	raakau_roto
20	Rakau akaoti	Rakau whakaoti	Rākau ʔakaoti	raakau_faka3#ʔoti
21	Korekore tutahi	Korekore tutahi	Korekore tūtaʔi	kore#kore_tuu#tahi
22	Korekore roto	Korekore roto	Korekore roto	kore#kore_roto
23	Korekore akaoti	Korekore whakaoti	Korekore ʔakaoti	kore#kore_faka3#ʔoti
24	Tangaroa tutai	Tangaroa tutahi	Taŋaroa tūtaʔi	taŋaroa_tuu#tahi
25	Tangaroa roto	Tangaroa roto	Taŋaroa roto	taŋaroa_roto
26	Tangaroa akaoti	Tangaroa whakaoti	Taŋaroa ʔakaoti	taŋaroa_faka3#ʔoti
27	Ko Tirongonui	Ko te Rongonui	Ko te Roŋo-nui	roŋo#nui6
28	Ko Tane	Tane	Tāne	taane
29	Ko Te Mauri	Mauri	Mauri	mauri
30	Ko Te mutunga	Mutunga	Mutuŋa	mutu#ŋa4

List 26: NCOOK_TGV (Tongareva/Penrhyn): Hiroa (1932: 216) (*23*) reported the following list, stating that "[n]o explanatory details could be obtained". Shibata (2003, also cited in POLLEX) provides the same list, but with forms of the nights' names that are clearly altered (*14*, *56*). For example, we used Tireo (documented in 1932) instead of Tiroe (reported in 2003), as this is most likely a late borrowing from the southern Cook Islands (specifically, see the list SCOOK_ATIU below).

	Hiroa (1932)	Shibata (2003)	Normalized
1	Tireo	tīroe	tireo/tireo
2	Hiro	hiro	firo
3	Soata	soata	so#ʔata
4	Tahi no te Samia	te tahi o te sāmita	tahi_maseʔa/samia
5	E roto no te Samia	te roto o te sāmita	roto_maseʔa/samia
6	E hakaoti no te Samia	te hakaoti o te sāmita	faka3#ʔoti_maseʔa/samia
7	E tahi o te Tamatea	te tahi o te tamatea	tahi_tamatea
8	E roto o te Tamatea	te roto o te tamatea	roto_tamatea
9	E hakaoti o te Tamatea	te hakaoti o te tamatea	faka3#ʔoti_tamatea
10	Huna	huunā	funa
11	Hoari [O Ari?]	hōari	ʔari/ʔari
12	Hua	hua	fua
13	Maharu	tamaharu [Ta maharu]	maharu
14	Atua	atia [atua]	atua
15	Hotu	hotu	fotu/fotu
16	Marangi	mārangi	maa2#raŋi
17	Turu	turu	turu
18	E tahi o te Rakau	te tahi o te rākau	tahi_raakau
19	E roto no te Rakau	to roto o te rākau	roto_raakau
20	E hakaoti o te Rakau	te hakaoti o te rākau	faka3#ʔoti_raakau
21	Te tahi o te Korekore	te tahi o te korekore	tahi_kore#kore
22	Te roto o te Korekore	te roto o te korekore	roto_kore#kore
23	Te hakaoti o te Korekore	te hakaoti o te korekore	faka3#ʔoti_kore#kore
24	Te tahi o te Tangaroa	te tahi o te tangaroa	tahi_taŋaroa
25	Te roto o te Tangaroa	te roto o te tangaroa	roto_taŋaroa
26	Te hakaoti o te Tangaroa	te hakaoti o te tangaroa	faka3#ʔoti_taŋaroa
27	Tane	tāne	taane
28	Mauri	maui	mauri
29	Rongonui	rongo nui	roŋo#nui6
30	Mutu	motu ~ mutu	mutu

List 27: SCOOK_ATIU (Atiu, southern Cook Islands). Reported by Mokoroa (1984: 124-5) (*57*). The author notes: “It has been suggested that *iro* and *mauriao* were added at the time of renewal contact with Tahiti in the 1840’s.” *Mauriao* is given as 31st night, and it means literally “Mauri of evening”. It may be a description of a part of the last day of the month, rather than the name of an additional night. This would make the list end with Mutu as the 30th night, like other lists in the Cook Islands.

	Original	Normalized
1	Tiroe	tireo/tiroe
2	[ʔ]Iro	firo
3	[ʔ]Oata	so#ʔata
4	Amiami	maseʔa/samia#maa
5	[ʔ]Akaoti Amiama	faka3#ʔoti_maseʔa/samia#maa
6	Tamatea	tamatea
7	[ʔ]Akaoti Tamatea	faka3#ʔoti_tamatea
8	Korekore	kore#kore
9	[ʔ]Akaoti Korekore	faka3#ʔoti_kore#kore
10	Vari	ʔari/vari
11	[ʔ]Una	funa
12	Ma[ʔ]aru	maharu
13	[ʔ]Ua	fua
14	Maitu	maitu
15	[ʔ]Otu	fotu
16	Marangi	maa2#raŋi
17	Turu	turu
18	Rakau ta[ʔ]i	raakau_tahi
19	Roto-rakau	roto_raakau
20	[ʔ]Akaoti-rakau	faka3#ʔoti_raakau
21	Korekore	kore#kore
22	Roto Korekore	roto_kore#kore
23	[ʔ]Akaoti Korekore	faka3#ʔoti_kore#kore
24	Tangaroa	taŋaroa
25	Roto Tangaroa	roto_taŋaroa
26	[ʔ]Akaoti Tangaroa	faka3#ʔoti_taŋaroa
27	Tane	taane
28	Rongonui	roŋo#nui6
29	Rongomauri	roŋo#mauri
30	Motu/Mutu	mutu
(31)	(Mauriao)	-

List 28: SCOOK_MGA (Mangaia, southern Cook Islands). In Gill (1876: 318; also Best 1922: 32-33, no. 10) (*13*, *58*). The list given as from Rarotonga (Cook Islands) in Tregear (1891: 666) is identical and must therefore be the same (*37*).

	Original	Normalized
1	[ʔ]Iro	firo
2	[ʔ]Oata	so#ʔata
3	[ʔ]Amiama	maseʔa/samia#maa
4	[ʔ]Amiama-akaoti	maseʔa/samia#maa_faka3#ʔoti
5	Tamatea	tamatea
6	Tamatea-[ʔ]akaoti	tamatea_faka3#ʔoti
7	Korekore	kore#kore
8	Korekore-[ʔ]akaoti	kore#kore_faka3#ʔoti
9	O Vari<br>(Vari-ma-te-takere = Originator-of-all-things)	ʔari/vari
10	Una [ʔuna]	funa
11	Maaru [Maʔaru]	maharu
12	Ua [ʔua]	fua
13	E atua (= A god)	atua
14	O Tu [ʔotu]	fotu/fotu
15	O Mārangi (Full Moon)	maa2#raŋi
16	Oturu	turu
17	Rakau	raakau
18	Rakau-roto	raakau_roto
19	Rakau-[ʔ]akaoti	raakau_faka3#ʔoti
20	Korekore	kore#kore
21	Korekore-roto	kore#kore_roto
22	Korekore-[ʔ]akaoti	kore#kore_faka3#ʔoti
23	Tangaroa	taŋaroa
24	Tangaroa-roto	taŋaroa_roto
25	Tangaroa-[ʔ]akaoti	taŋaroa_faka3#ʔoti
26	O Tane	taane
27	Rongo-Nui	roŋo#nui6
28	Mauri	mauri
29	Omūtu (=ended)	mutu
30	Otireo / Otire o [ʔ]Avaiki<br>("Lost in the depths of Avaiki")	tireo/tireo#ʔavaiki

List 29: SCOOK_RARO1 (Rarotonga). For this Rarotongan list, Tregear (1891: 666) gave Atua and Maitu as alternative names of the 13th night (*37*). The version with Atua is therefore identical to SCOOK_MGA. Accordingly, we have considered the version with Maitu.

	Original	Normalized
1	[ʔ]Iro	firo
2	[ʔ]Oata	so#ʔata
3	[ʔ]Amiama	maseʔa/samia#maa
4	[ʔ]Amiama [ʔ]aka oti	maseʔa/samia#maa faka3#ʔoti
5	Tamatea	tamatea
6	Tamatea [ʔ]aka oti	tamatea faka3#ʔoti
7	Korekore	kore#kore
8	Korekore [ʔ]aka oti	kore#kore faka3#ʔoti
9	O Vari	ʔari/vari
10	[ʔ]Una	funa
11	Ma[ʔ]aru	maharu
12	[ʔ]Ua	fua
13	E atua (or Maitu)	maitu
14	O Tu [ʔotu]	fotu/fotu
15	Marangi	maa2#raŋi
16	Oturu	turu
17	Rakau	raakau
18	Rakau-roto	raakau roto
19	Rakau [ʔ]aka oti	raakau_faka3#ʔoti
20	Korekore	kore#kore
21	Korekore-roto	kore#kore_roto
22	Korekore-[ʔ]akaoti	kore#kore_faka3#ʔoti
23	Tangaroa	taŋaroa
24	Tangaroa roto	taŋaroa_roto
25	Tangaroa [ʔ]aka oti	taŋaroa faka3#ʔoti
26	O Tane	taane
27	Rongo-Nui	roŋo#nui6
28	Mauri	mauri
29	O Mutu	mutu
30	Otireo / Otire o [ʔ]Avaiki (“Lost in the depths of Avaiki”)	tireo/tireo#ʔavaiki

List 30: SCOOK_RARO2 (Rarotonga). S. Percy Smith apud Williams (1928: 356, no. 35) (*44*). “Mauri,” annotated as “? Maure,” is surely a misprint for Ma[ʔ]aru, based on its position vis-à-vis similar calendars.

	Original	Normalized
1	Tireo	tireo/tireo
2	[ʔ]Iro	firo
3	[ʔ]Oata	so#ʔata
4	[ʔ]Amiama	maseʔa/samia#maa
5	([ʔ]Amiama) [ʔ]Akaoti	maseʔa/samia#maa_faka3#ʔoti
6	Tamatea ta[ʔ]i	tamatea_tahi
7	Tamatea	tamatea
8	Korekore ta[ʔ]i	kore#kore_tahi
9	[ʔ]Akaoti te Korekore	faka3#ʔoti_kore#kore
10	O Vari	ʔari/vari
11	[ʔ]Una	funa
12	Mauri (? Maure)	maharu
13	[ʔ]Ua	fua
14	Maitu	maitu
15	[ʔ]Otu	fotu/fotu
16	Marangi	maa2#raŋi
17	Oturu	turu
18	Rakau ta[ʔ]i	raakau_tahi
19	Roto te Rakau	roto_raakau
20	[ʔ]Akaoti te Rakau	faka3#ʔoti_raakau
21	Korekore ta[ʔ]i	kore#kore_tahi
22	Roto te Korekore	roto_kore#kore
23	[ʔ]Akaoti te Korekore	faka3#ʔoti_kore#kore
24	Tangaroa ta[ʔ]i	taŋaroa_tahi
25	Tangaroa roto	roto_taŋaroa
26	[ʔ]Akaoti te Tangaroa	faka3#ʔoti_taŋaroa
27	Rongonui	roŋo#nui6
28	Tane	taane
29	Mauri	mauri
30	O Mutu	mutu

List 31: SCOOK_RARO3 (Rarotonga): Reconstructed from the dictionary entries of Buse (1996) (*59*), in which some nights are given with uncertainty, and by comparison with earlier records (see SCOOK_RARO1a-b):

- *'iro* = "name of the second (?) night" (p. 132).
- *'ōata* = "the third in the series (according to most reckonings)" (p. 278).
- *tamatea* = "fourth to sixth nights"; *tamatea ta'i*, *roto tamatea*, *'akaoti tamatea* (p. 436).
- *korekore* = "approximately the seventh to the ninth nights and the nineteenth to the twenty-first, but usage varies"; *korekore ta'i*, *roto korekore* , *'akaoti korekore* (pp. 193-4).
- *'ōvari* = "one of the nights of the moon, the tenth in some reckonings" (p. 296)
- *ma'aru* = "one of the nights of the moon, the eleventh or twelfth according to some" (p. 207)
- *maitu* = "one of the nights of the moon, according to some, the fourteenth" (p. 214).
- *'ōtu* = "one of the nights of the moon: according to some reckonings, the fourteenth night, and forming with *turuki* (the fifteenth night) the period of *mārangi* (full moon)" (p. 295)
- *turu* = "Sixteenth night of the moon = *tūruki*" (pp. 530-1).
- *rākau* = "A phase of the moon"; *rākau ta'i* "sixteenth night", *roto te rākau* "seventeenth night"; *'akaoti te rākau* "eighteenth night" (p. 378).
- *Tangaroa* = "A phase of the moon, the twenty-second to the twenty-fourth nights"; *Tangaroa ta'i* "twenty-second", *roto tangaroa* "twenty-third"; *'akaoti tangaroa* "twenty-fourth" (p. 415).
- *'ōtāne* = "one of the nights of the moon, according to some accounts the twenty seventh or twenty fifth night" (p. 294)
- *rongonui* = "the twenty-sixth night" (p. 398).
- *mauri mate* = "one of the nights of the moon, the twenty-seventh or twenty-ninth, according to some reckonings" (p. 243).

This list can be matched neatly with other sets from Rarotonga if we assume that: (1) some of the missing nights not included in the dictionary occupied the same positions as the earlier list (SCOOK_RARO1a-b); (2) *marangi*, mentioned as the period of the full moon with *'otu* and *turu*, was actually a night in its own right. This resolves some of the ambiguities and contradictions of the definitions provided by Buse.

Hence, the resulting list is as follows:

	Original	Normalized
1	[Tireoʔ]	tireo/tireo
2	ʔIro	firo
3	ʔōata	so#ʔata
4	Tamatea taʔi	tamatea_tahi
5	Roto tamatea	roto_tamatea
6	ʔAkaoti tamatea	faka3#ʔoti_tamatea
7	Korekore taʔi	kore#kore_tahi
8	Roto korekore	roto_kore#kore
9	ʔAkaoti korekore	faka3#ʔoti_kore#kore
10	ʔOvari	ʔari/vari
11	[ʔunaʔ]	funa
12	Maʔaru	maharu
13	Maitu	maitu
14	ʔOtu	fotu/fotu
15	Mārangi (full moon)	maa2#raŋi
16	Turu = Tūruki	turu
17	Rākau taʔi	raakau_tahi
18	Roto te rākau	roto_raakau
19	ʔAkaoti te rākau	faka3#ʔoti_raakau
20	Korekore taʔi	kore#kore_tahi
21	Roto korekore	roto_kore#kore
22	ʔAkaoti korekore	faka3#ʔoti_kore#kore
23	Tangaroa taʔi	taŋaroa_tahi
24	Roto tangaroa	roto_taŋaroa
25	ʔAkaoti tangaroa	faka3#ʔoti_taŋaroa
26	ʔōtane	taane
27	Rongonui	roŋo#nui6
28	Mauri mate	mauri#mate2
29	[Mutuʔ]	mutu

List 32: RPN1 (Rapa Nui). Collected by Thomson in 1886 (published in Thomson 1889: 546) (*60*). "Tueo" is a misprint for Tireo, probably due to the misinterpretation of a handwritten manuscript. The possessive particle *O* is included and attached to the name of several names (e.g., "Ohotu" for "O Hotu"). Only 29 nights are listed and *Hiro* (which is reported in other Rapa Nui lists and present in calendars from other islands) is missing. Thomson did not number the list, but he gave *Oari* as the new moon.

	Original [annotated]	Normalized
1	Oari (New Moon)	ʔari/ʔari
2	Kokore tahi	ko#kore_tahi
3	Kokore rua	ko#kore_rua
4	Kokore toru	ko#kore_toru
5	Kokore ha [Kokore haa]	ko#kore_faa
6	Kokore rima	ko#kore_rima
7	Kokore ono	ko#kore_ono
8	Maharu	maharu
9	Ohua	fua
10	Otua	atua
11	Ohotu	fotu/fotu
12	Maure	maure
13	Ina-ira	inaira
14	Ra Kau	raakau
15	Omotohi (Full Moon)	matofi
16	Kokore tahi	ko#kore_tahi
17	Kokore rua	ko#kore_rua
18	Kokore toru	ko#kore_toru
19	Kokore ha	ko#kore_faa
20	Kokore rima	ko#kore_rima
21	Tapume	tapume
22	Matua	matua
23	Orongo	roŋo
24	Orongo taane	roŋo#taane
25	Mauri nui	mauri#nui5
26	Marui Kero [Mauri Kero]	mauri#kero
27	Omutu	mutu
28	Tueo [Tireo]	tireo/tireo
29	Oata	so#ʔata

List 33: RPN2 (Rapa Nui). Reported by A. Métraux (1940: 50-51) (*17*). Thomson is cited as a source, but there are differences in the list, most notably the inclusion of *Hiro* as second night. Métraux wrote Thomson's versions of some names in parenthesis.

	Original	Normalized
1	Tireo	tireo/tireo
2	Hiro	firo
3	Ata (Oata)	so#ʔata
4	Ari (Oari)	ʔari/ʔari
5	Kokore tahi	ko#kore_tahi
6	Kokore rua	ko#kore_rua
7	Kokore toru	ko#kore_toru
8	Kokore ha	ko#kore_faa
9	Kokore rima	ko#kore_rima
10	Kokore ono	ko#kore_ono
11	Maharu	maharu
12	Hua (Ohua)	fua
13	Atua (Otua)	atua
14	Hotu (Ohotu)	fotu/fotu
15	Maure	maure
16	Ina-ira	inaira
17	Rakau	raakau
18	Motohi	matofi
19	Kokore tahi	ko#kore_tahi
20	Kokore rua	ko#kore_rua
21	Kokore toru	ko#kore_toru
22	Kokore ha	ko#kore_faa
23	Kokore rima	ko#kore_rima
24	Tapume	tapume
25	Matua	matua
26	Rongo (Orongo)	roŋo
27	Rongo Tane (Orongo Taane)	roŋo#taane
28	Mauri-nui	mauri#nui5
29	Mauri-kero	mauri#kero
30	Mutu (Omutu)	mutu

List 34: RPN3 (Rapa Nui). Reported by Englert (1974 [1948]: 243-44), presumably based on fieldwork conducted in the 1930s, but also compared against the list of Thomson (*61*). While *Ohiro* (*Hiro*) was given to him as the "new moon," apparently, he knew of *Oari* only from Thomson and marked it with a question mark in parenthesis. Notably, even with *Oari*, the list would contain only 29 nights, because Englert did not include *Hotu* (probably a misprint?).

	Original	Normalized
(1-)2	(Oari?) Ohiro (new moon)	ʔari/ʔari
		firo
3	Kokore tahi	ko#kore_tahi
4	Kokore rua	ko#kore_rua
5	Kokore toru	ko#kore_toru
6	Kokore há	ko#kore_faa
7	Kokore rima	ko#kore_rima
8	Kokore ono	ko#kore_ono
9	Maharu	maharu
10	Ohua	fua
11	Otua	atua
12	Maúre	maure
13	Ina-Ira	inaira
14	Rakau	raakau
15	Omotohi (full moon)	matofi
16	Kokore tahi	ko#kore_tahi
17	Kokore rua	ko#kore_rua
18	Kokore toru	ko#kore_toru
19	Kokore ha	ko#kore_faa
20	Kokore rima	ko#kore_rima
21	Tapume	tapume
22	Matua	matua
23	Orongo	roŋo
24	Orongo Taane	roŋo#taane
25	Maúri-nui	mauri#nui5
26	Maúri kero	mauri#kero
27	Omutu	mutu
28	Tireo	tireo/tireo
29	Oata [ʔohata]	so#ʔata

List 35: RPN4 (Rapa Nui). In a manuscript by the native Esteban Atan, dated from 1936. It was found in 1955-56 and published in Kondratov (1965) (*62*). See also the commentary in Horley (2011) (*9*). The list is clearly related to the older calendars given above, but also deviates a great deal from it, as if reflecting the loss of remembrance.

	Original [Interpretation]	Normalized
1	hetahi kokore [he tahi kokore "the first kokore"]	tahi_ko#kore
2	herua kokore [he rua kokore "the second kokore"]	rua_ko#kore
3	hetoru kokore [he toru kokore "the third kokore"]	toru_ko#kore
4	hehá kokore [he haa kokore "the fourth kokore"]	faa_ko#kore
5	herima kokore [he rima kokore "the fifth kokore"]	rima_ko#kore
6	heóno kokore [he ono kokore "the sixth kokore"]	ono_ko#kore
7	hehitu kokore [he hitu kokore "the seventh kokore"]	fitu_ko#kore
8	hevaú te o hua [he vaʔu te o hua "the eight of Hua"]	waru_fua
9	heharu teá [he haru tea]	haru_tea1
10	hepopo tea [he poopoo tea "the white p."]	popo_tea1
11	hepopo mea [he poopoo mea "the red p."]	popo_mea
12	hepopo úri [he poopoo uri "the dark p."]	popo_ʔuri
13	hepopo hega [he poopoo heŋa "the reddish p."]	popo_heŋa
14	heraa kaú [he raakau]	raakau
15	heomo tohi [he o motohi] (full moon)	matofi
16	he maúre	maure
17	heó hiró [he o hiro]	firo
18	herua te o hiro [he rua te o hiro "the second O Hiro"]	rua_firo
19	he toru teo hiro [he toru te o hiro "the third O Hiro"]	toru_firo
20	he ha teo hiro [he haa te o hiro "the fourth O Hiro"]	faa_firo
21	he rimá teo hiro [he rima te o hiro "the fifth O Hiro"]	rima_firo
22	he óno teo hiro [he ono te o hiro "the sixth O Hiro"]	ono_firo
23	he ohea	ohea
24	he ohaú	ohau
25	he ohuri	ohuri
26	o ari	ʔari/ʔari
27	o ata [ʔohata]	so#ʔata
28	a raga	a#raŋa
29	a tai	a#tai
30	The manuscript shows a scratched crescent	-

List 36: TAH1 (Unspecified location). Reported by Ellis (1829: 420) (*63*). It also appears in Fornander (1878: 126) where it is attributed to the "Society Islands" (*36*). Williams (1928: 356, no. 36) gave this list "with corrections from the dictionary" (these corrections are given here in parenthesis), so the original forms are easily restored (*44*). It is remarkably similar to, but not identical with, TAH2 and TAH3.

	Original	Analytical transcription	Normalized
1	Ohirohiti	O Hiro-hiti	firo#fiti
2	Hoata	Hoata	so#ʔata
3	Hami-ami-mua (Hamiama-mua)	Hamiama-mua	maseʔa/samia#maa_muʔa
4	Hami-ami-roto (Hamiama-roto)	Hamiama-roto	maseʔa/samia#maa_roto
5	Hami-ami-muré (Hamiama-muri)	Hamiama-muri	maseʔa/samia#maa_muri
6	Ore-ore-mua	ʔOreʔore-mua	kore#kore_muʔa
7	Ore-ore-muri	ʔOreʔore-muri	kore#kore_muri
8	Tamatea	Tamatea	tamatea
9	Ohuna	O Huna	funa
10	Oari	O Ari	ʔari/ʔari
11	Omaharu	O Maharu	maharu
12	Ohua	O Hua	fua
13	Omaidu (Maitu)	O Maitu	maitu
14	Ohodu (Hotu)	O Hotu	fotu/fotu
15	Omarae (Mara'i). Te-maramaati (FM)	O maraʔi	maa2#raŋi
16	Oturu-tea	O Turu-tea	turu#tea2
17	Raau-mua	Rāʔau-mua	raakau_muʔa
18	Raau-roto	Rāʔau-roto	raakau_roto
19	Raau-muri	Rāʔau-muri	raakau_muri
20	Ore-ore-mua	ʔOreʔore-mua	kore#kore_muʔa
21	Ore-ore-roto	ʔOreʔore-roto	kore#kore_roto
22	Ore-ore-muri	ʔOreʔore-muri	kore#kore_muri
23	Taaroa-mua	Taʔaroa-mua	taŋaroa_muʔa
24	Taaroa-roto	Taʔaroa-roto	taŋaroa_roto
25	Taaroa-muri	Taʔaroa-muri	taŋaroa_muri
26	O-Tane	O Tāne	taane
27	O-Roomie (Roonui)	O Roʔo-nui	roŋo#nui6
28	O-Roomaori	O Roʔo-Mauri	roŋo#mauri
29	O-mutu	O Mutu	mutu
30	O-Terieo	O Tireo	tireo/tireo

List 37: TAH2 (Paparā, Tahiti). The first of three lists compiled in Stimson (1928): "originally recorded by the High Chief of Papara, Teuraiterai i Taputuarai, known as Tati, le Grand, and was confided to the writer by his great-grand-daughter, Marautaaroa i Tahiti, ex-Queen of Tahiti" (*12*).

	Original	Analytical transcription	Normalized
1	Tirio	Tireo	tireo/tireo
2	Hiro-hiti	Hiro-hiti	firo#fiti
3	Hoata	Hoata	so#ʔata
4	Hamiama-mua	Hamiama-mua	maseʔa/samia#maa_muʔa
5	Hamiama-roto	Hamiama-roto	maseʔa/samia#maa_roto
6	Hamiama-muri	Hamiama-muri	maseʔa/samia#maa_muri
7	'Ore'ore-mua	ʔOreʔore-mua	kore#kore_muʔa
8	'Ore'ore-muri	ʔOreʔore-muri	kore#kore_muri
9	Tamatea	Tamatea	tamatea
10	Huna	Huna	funa
11	Rapu	Rapu	rapu
12	Maharu	Maharu	maharu
13	Hu'a	Hua	fua
14	Maitu	Maitu	maitu
15	Hotu	Hotu	fotu/fotu
16	Mara'i	Maraʔi	maa2#raŋi
17	Turu	Turu	turu
18	Arä'āu-tahi	Rāʔau-mua	raakau_muʔa
19	Arä'āu-roto	Rāʔau-roto	raakau_roto
20	Arä'āu-muri	Rāʔau-muri	raakau_muri
21	Ore'ore-mua	ʔOreʔore-mua	kore#kore_muʔa
22	Ore'ore-roto	ʔOreʔore-roto	kore#kore_roto
23	Ore'ore-muri	ʔOreʔore-muri	kore#kore_muri
24	Ta'aroa-mua	Taʔaroa-mua	taŋaroa_muʔa
25	Ta'aroa-roto	Taʔaroa-roto	taŋaroa_roto
26	Ta'aroa-muri	Taʔaroa-muri	taŋaroa_muri
27	Tāne	O Tāne	taane
28	Ro'o-nui	O Roʔo-nui	roŋo#nui6
29	Ro'o-mauri	O Roʔo-Mauri	roŋo#mauri
30	Mauri-mate	Mauri-mate	mauri#mate2

List 38: **TAH3** (Mai'ao, Tahiti). The second of three lists collected by Stimson (1928): "given by Paruparu, son of the hereditary High Chief of Maiao (Saunder's Island)" (12).

	Original	Normalized
1	Tireo	tireo/tireo
2	Hiro-hiti	firo#fiti
3	Hoata	so#ʔata
4	Hamiama-mua	maseʔa/samia#maa_muʔa
5	Hamiama-roto	maseʔa/samia#maa_roto
6	Hamiama-muri	maseʔa/samia#maa_muri
7	Tamatea-mua	tamatea_muʔa
8	Tamatea-roto	tamatea_roto
9	Tamatea-muri	tamatea_muri
10	Huna	funa
11	'Ari	ʔari/ʔari
12	Maharu	maharu
13	Hua	fua
14	Maitu	maitu
15	Hotu	fotu/fotu
16	Mara'i	maa2#raŋi
17	Turu	turu
18	Rā'āu-mua	raakau_muʔa
19	Rā'āu-roto	raakau_roto
20	Rā'āu-muri	raakau_muri
21	Ore'ore-mua	kore#kore_muʔa
22	Ore'ore-roto	kore#kore_roto
23	Ore'ore-muri	kore#kore_muri
24	Ta'aroa-mua	taŋaroa_muʔa
25	Ta'aroa-roto	taŋaroa_roto
26	Ta'aroa-muri	taŋaroa_muri
27	Tāne	taane
28	Ro'o-nui	roŋo#nui6
29	Ro'o-mauri	roŋo#mauri
30	Mutu	mutu

List 39: TAH4 (Mo’orea and Tahiti islands). The third of the three lists gathered by Stimson (1928), and which was “given by various high authorities of Tahiti and Moorea” (12).

	Original	Normalized
1	Tireo	tireo/tireo
2	Hiro-hiti	firo#fiti
3	Hoata	so#ʔata
4	Hamiama-tahi	maseʔa/samia#maa_tahi
5	Hamiama-roto	maseʔa/samia#maa_roto
6	Fa'aoti-Hamiama	faka3#ʔoti_maseʔa/samia#maa
7	Ore'ore-tahi	kore#kore_tahi
8	Fa'aoti-?ore?ore	faka3#ʔoti_kore#kore
9	Tamatea	tamatea
10	Huna	funa
11	Rapu	rapu
12	Mahoru	maharu
13	Hua	fua
14	Maitu	maitu
15	Hotu	fotu/fotu
16	Mara'i	maa2#raŋi
17	Turu-tea	turu#tea2
18	Rā'āu-tahi	raakau_tahi
19	Rā'āu-roto	raakau_roto
20	Fa'aoti-Rā'āu	faka3#ʔoti_raakau
21	?Ore'ore-tahi	kore#kore_tahi
22	Ore'ore-roto	kore#kore_roto
23	Fa'aoti-?ore?ore	faka3#ʔoti_kore#kore
24	Ta'aroa-tahi	taŋaroa_tahi
25	Ta'aroa-roto	taŋaroa_roto
26	Fa'aoti-Ta'aroa	faka3#ʔoti_taŋaroa
27	Tāne	taane
28	Ro'o-nui	roŋo#nui6
29	Ro'o-mauri	roŋo#mauri
30	Motu	mutu

List 40: TUA1 (Anaa island, Tuamotu). The entries in the Tuamotuan dictionary of Stimson (1964) mention at least two different lists of the nights of the moon from the island of Anaa. Stimson spent only three days on the island but reportedly collaborated with a native informant from Anaa for many years while he stayed in Tahiti (Marshall *apud* Stimson 1964: 39) (64). If we extract and summarize the relevant entries from the dictionary, we obtain the following information on the names of the nights from Anaa:

- Ari = 11th night
- Fakaata = 1st, 3rd, or 9th night, according to different lists
- Fakaoti raakau = 11th night, but island not given; may or may not be in the TUA1 list
- Hania-fakaoti = 14th night
- Hania-roto = 13th night
- Hania-tahi = 12th night
- Hiro = 3rd night
- Hiro-hiti = 2nd night; should not co-exist with previous
- Hotu = 15th night in one list; 17th night in another list
- Hua = 19th night according to two lists
- Huna = given as the 'thirtieth' (30th) night, but this must be a mistake for 13th: Huna is always located in positions 8th-11th in other calendars, never at the end
- Te-Kohuunga-o-Marama = 14th night in one list
- Korekore-fakaoti = 25th night; same as fakaoti-korekore in other islands.
- Korekore-rari = 23rd night
- Korekore-roto = 25th night in two lists; if this is correct, then this should be night 24th, before korekore-fakaoti and after korekore-rari
- Korekore-tahi = 23rd night, so apparently an alternative name for korekore-rari
- Maharu = 12th night (NB: given as from the island of ROI in the dictionary, but probably ANA (Anaa) is meant, because ROI has a separate sub-entry)
- Maaraŋi = 15th night and full moon in one list; 16th night in 'other lists'
- Mararo = 29th night in 'one list'; 30th night in 'one list'
- Motu = 30th night; no island specified, so it might be part of at least one Anaa list, if not all
- Oŋa = 12th night in 'one list'
- Penu = 15th or 17th night according to 'varying lists'
- Piriroa = 13th night in 'one list'
- Raakau-fakaoti = 22nd night according to 'two lists'; we deduce from the following entries that this was also the name of night 11th in 'two lists'
- Raakau-rari = 9th and 20th night (= there are two nights with the same name)
- Raakau-roro = 10th and 21st night (no island is given, but we must deduce this existed in two lists from Anaa)
- Rapu = one of the nights; no island is specified, but it is possible that it was part of the Anaa lists (in some empty slot?)
- Raukuru = 10th night in 'one list' (probably from the same list as the following)
- Rautangeea = 12 night in 'one list' (probably from the same list as the previous)
- Roŋo-nui = 27th or 28th night according to different calendars (island not given, but probably refers also to Anaa)
- Taaiva-fakaoti = 28th night

- Taaiva-rari = 26th night
- Taaiva-roto = 27th night
- Taaiva-tahi = 26th night, alternative name of Taaiva-rari
- Tamatea = 7th or 8th night, according to different lists
- Tika-raakau = 6th night in 'one' list
- Tuu = either 1st or 7th night, according to different lists
- Turu = 18th night, according to 'two' lists
- Turuma = one of the nights, so it can be in any position (= Turu? so discard?)
- Vaitu-fakaoti = 5th or 6th night, according to different lists
- Vaitu-rari = 4th or 5th night, according to different lists
- Vaitu-roto = 4th or 5th night, according to different lists
- Vari = 8th or 9th night, according to different lists

Additionally, we can deduce the following constraints:
- Ari = Vari and Hiro = Hiro-hiti: i.e., these forms should not co-exist in the same list.
- Fakaata (< **faka-ʔata* 'reflection'?): 1st or 3rd position is preferrable because it is probably cognate with Marquesan Tuu-haka(h)oata (< **faka-so*(-)*ʔata* 'cast shadow') and Hoata (< *soʔata* 'bright') from the beginning or end of other calendrical lists. For the etymology of these forms, see section S1C.
- Vaitu-rari means literally 'Vaitu One' and must come before Vaitu-roto 'Vaitu inside' and Vaitu-fakaoti 'Vaitu finalising.'

At least one list can be reconstructed that is fully consistent with this report, although in this version the night Huna appears unexpectedly as the final night of the sequence, a feature that is unlikely and should be excluded. The resulting list is as follows:

	Original	Normalized
1	Tuu	tuu
2	Hiro-hiti	firo#fiti
3	Fakaata	faka1#ʔata
4	Vaitu-rari	vaitu_rari
5	Vaitu-roto	vaitu_roto
6	Vaitu-fakaoti	vaitu_faka3#ʔoti
7	Tamatea	tamatea
8	Vari	ʔari/vari
9	Raakau-rari	raakau_rari
10	Raakau-roto	raakau_roto
11	Raakau-fakaoti	raakau_faka3#ʔoti
12	Hania-tahi	maseʔa/samia_tahi
13	Hania-roto	maseʔa/samia_roto
14	Hania-fakaoti	maseʔa/samia_faka3#ʔoti
15	Hotu	fotu/fotu
16	Maaraŋi	maa2#raŋi
17	Penu	penu
18	Turu	turu
19	Hua	fua
20	Raakau-rari	raakau_rari
21	Raakau-roto	raakau_roto
22	Raakau-fakaoti	raakau_faka3#ʔoti
23	Korekore-rari	kore#kore_rari
24	Korekore-roto	kore#kore_roto
25	Korekore-fakaoti	kore#kore_faka3#ʔoti
26	Taaiva-rari	taaiva_rari
27	Taaiva-roto	taaiva_roto
28	Taaiva-fakaoti	taaiva_faka3#ʔoti
29	Mararo	mararo
30	Huna (Mararo / Motu)	-

List 41: TUA2 (Fakahaina island, Tuamotu). Given by Audran (1929), who attributes it to Bernard Maui "from Kakahina" (*53*). This is most probably a misprint for "Fakahina." Rongomatitiro is surely Rongo-ma-titiro, which we notated as 'roŋo_titiro' without the particle.

	Original	Normalized
1	Rogomatitiro	roŋo_titiro
2	Hiro	firo
3	Hoata	so#ʔata
4	Hania-tahi	maseʔa/samia_tahi
5	Hania-roto	maseʔa/samia_roto
6	Hania-fakaoti	maseʔa/samia_faka3#ʔoti
7	Korekore-tahi	kore#kore_tahi
8	Korekore-fakaoti	kore#kore_faka3#ʔoti
9	O Tamatea	tamatea
10	O Vari	ʔari/vari
11	O Hua	fua
12	O Huna	funa
13	O Maharu	maharu
14	O Maitu	maitu
15	O Hotu	fotu/fotu
16	O Maragi	maa2#raŋi
17	O Turu	turu
18	Rakau tahi	raakau_tahi
19	Rakau roto	raakau_roto
20	Rakau fakaoti	raakau_faka3#ʔoti
21	Korekore tahi	kore#kore_tahi
22	Korekore roto	kore#kore_roto
23	Korekore fakaoti	kore#kore_faka3#ʔoti
24	Tagaroa-tahi	taŋaroa_tahi
25	Tagaroa-roto	taŋaroa_roto
26	Takaroa fakaoti (sic)	taŋaroa_faka3#ʔoti
27	O tikatika	tika/tikatika
28	O Rogo-nui	roŋo#nui6
29	O Rogo mauri	roŋo#mauri
30	O Kero	kero

List 42: TUA3 (Hao Island, Tuamotu). Gathered by Audran (1929), from Torohia a Fakirua "from Hao" (*53*). As in other Tuamotuan lists, a night called *Tinai ki te pō* is given in 31st position, which entails a set longer than the lunar cycle. Since this literally means "Kill(ing) in the night," it is most likely a description of the end of the moon cycle rather than a night of the month. Accordingly, we did not consider it for our purposes. Another particularity of this list is that two alternative names are given for the 2nd and 3rd nights, but they are redundant with the 3rd and 4th, respectively. More specifically: *O Hania-tahi* is given alongside *O Hoata* as the names of the 3rd night, yet the former surely did not coexist with *Te taiga na Hania* (4th), which means essentially the same: 'Hania One' or 'The Oneness of Hania'. Therefore, we did not consider these two deviations. Finally, "Mauri kere" must be a misprint for "Mauri kero."

	Original	Normalized
1	O Tipi	tipi
2	O Hoata [/] O Hiro	firo
3	O Hania-tahi [/] O Hoata	so#ʔata
4	Ta tahiga na Hania	tahi#ŋa1_maseʔa/samia
5	Te rotoga na Hania	roto#ŋa2_maseʔa/samia
6	Te fakaotiga na Hania	faka3#ʔoti#ŋa3_maseʔa/samia
7	Te tahiga na kore	tahi#ŋa1_kore
8	Te rotoga na kore	roto#ŋa2_kore
9	Te hak[a]otiga na kore	faka3#ʔoti#ŋa3_kore
10	O Vari	ʔari/vari
11	O Tamatea	tamatea
12	O Hua	fua
13	O Huna	funa
14	O Maharu	maharu
15	O Maitu	maitu
16	O Hotu	fotu/fotu
17	O Maragi	maa2#raŋi
18	O Turu (O Pahoro)	turu
19	Te tahiga na rakau	tahi#ŋa1_raakau
20	Te rotoga na rakau	roto#ŋa2_raakau
21	Te hakaotiga na rakau	faka3#ʔoti#ŋa3_raakau
22	Te tahiga na Taiva	tahi#ŋa1_taaiva
23	Te rotoga na Taiva	roto#ŋa2_taaiva
24	Te otiga na Taiva	ʔoti#ŋa3_taaiva
25	Te Tahiga na tagaroa	tahi#ŋa1_taŋaroa
26	Te rotoga na tagaroa	roto#ŋa2_taŋaroa
27	Te hakaotiga na tagaroa	faka3#ʔoti#ŋa3_taŋaroa
28	O Rogo-nui	roŋo#nui6
29	O Rogo mauri	roŋo#mauri
30	Mauri kere [Mauri kero]	mauri#kero
31	Tinai ki te po	-

List 43: TUA4 (Hao Island, Tuamotu). Gathered by Audran (1929), from Pou a Ganahoa, "from Hao" (*53*). The last night, "hari-kero," is certainly a mistake for the expected "Mauri kero" seen in other lists from Hao (see TUA3 and TUA5).

	Original	Normalized
1	O Tipi	tipi
2	O Hiro	firo
3	O Hoata	so#ʔata
4	Hania-tahi	maseʔa/samia_tahi
5	Hania-roto	maseʔa/samia_roto
6	Hania fakaoti	maseʔa/samia_faka3#ʔoti
7	Kore-tahi	kore_tahi
8	Kore-roto	kore_roto
9	Kore-fakaoti	kore_faka3#ʔoti
10	O Tamatea	tamatea
11	O Vari	ʔari/vari
12	O Huna	funa
13	O Hua	fua
14	O Mahuru [Maharu]	maharu
15	O Maitu	maitu
16	O Maragi	maa2#raŋi
17	O Hotu	fotu/fotu
18	Rakau tahi	raakau_tahi
19	Rakau roto	raakau_roto
20	Rakau fakaoti	raakau_faka3#ʔoti
21	Kore(-)tahi	kore_tahi
22	Kore-roto	kore_roto
23	Kore-fakaoti	kore_faka3#ʔoti
24	Hania-tahi	maseʔa/samia_tahi
25	Hania-roto	maseʔa/samia_roto
26	Hania-fakaoti	maseʔa/samia_faka3#ʔoti
27	Tahi tagaroa	tahi_taŋaroa
28	O Rogo-nui	roŋo#nui6
29	O Rogo mauri	roŋo#mauri
30	O hari-kero [mauri-kero]	mauri#kero

List 44: TUA5 (Hao Island, Tuamotu). Collected by Audran (1929), from F. Teregomaihiti, "from Hao" (*53*). Here we find "Nakore," reflecting surely *na Kore* 'of Kore,' as shown also by the presence of "Na Tagaroa" and "Na taiva" instead of plain "Tagaroa" and "Taaiva." The list has 32 items, yet in addition to discarding "Tinai ki te po" (see TUA3), we should probably assume that "O Rogo mauri tia" and "O Rogo mauri kakere" originally referred to the same night, i.e., plain Rongo-mauri.

	Original	Normalized
1	O Tipi	tipi
2	O Hoata	so#ʔata
3	Hania tahi	maseʔa/samia_tahi
4	Hania roto	maseʔa/samia_roto
5	Hania hakaoti	maseʔa/samia_faka3#ʔoti
6	Nakore tahi	kore_tahi
7	Nakore roto	kore_roto
8	Nakore hakaoti	kore_faka3#ʔoti
9	O Vari	ʔari/vari
10	O Tamatea	tamatea
11	O Hua	fua
12	O Huna	funa
13	O Maharu	maharu
14	O Maitu	maitu
15	O Hotu	fotu/fotu
16	O Maragi	maa2#raŋi
17	Pahoro	pahoro
18	Rakau tahi	raakau_tahi
19	Rakau roto	raakau_roto
20	Rakau hakaoti	raakau_faka3#ʔoti
21	Nakore tahi	kore_tahi
22	Nakore roto	kore_roto
23	Nakore hakaoti	kore_faka3#ʔoti
24	Na tagaroa tahi	taŋaroa_tahi
25	Na tagaroa roto	taŋaroa_roto
26	Na tagaroa hakaoti	taŋaroa_faka3#ʔoti
27	O Rogo mauri tia	roŋo#mauri#tia
28	O Rogo mauri kakere	roŋo#mauri#kakere
29	Na taiva tahi	taaiva_tahi
30	Na taiva roto	taaiva_roto
31	Na taiva hakaoti	taaiva_faka3#ʔoti
32	Tinai ki te po	-

List 45: TUA6 (Hao, Island Tuamotu). Collected by Audran (1929), from Huri a Tuteamaru, "from Hao" (*53*). As per above, "Tinai ki te po" was not counted as a night (see TUA3).

	Original	Normalized
1	O Tipi	tipi
2	O Hiro	firo
3	O Hoata	so#ʔata
4	Hania-tahi	maseʔa/samia_tahi
5	Hania-roto	maseʔa/samia_roto
6	Hania-fakaoti	maseʔa/samia_faka3#ʔoti
7	Kore-kore tahi	kore#kore_tahi
8	Korekore roto	kore#kore_roto
9	Korekore-fakaoti	kore#kore_faka3#ʔoti
10	O Tamatea	tamatea
11	O Vari	ʔari/vari
12	O Huna	funa
13	O Hua	fua
14	O Maharu	maharu
15	O Maitu	maitu
16	O Hotu	fotu/fotu
17	O Maragi	maa2#raŋi
18	O Turu	turu
19	Rakau-tahi	raakau_tahi
20	Rakau-roto	raakau_roto
21	Ra[ka]u-fakaoti	raakau_faka3#ʔoti
22	Korekore-tahi	kore#kore_tahi
23	Korekore-roto	kore#kore_roto
24	Korekore fakaoti	kore#kore_faka3#ʔoti
25	Tagaroa-tahi	taŋaroa_tahi
26	Tagaroa-roto	taŋaroa_roto
27	Tagaroa fakaoti	taŋaroa_faka3#ʔoti
28	O tika	tika/tika
29	O Rogonui	roŋo#nui6
30	O Rogomauri	roŋo#mauri
31	Mauri kero	mauri#kero
32	Tinai ki te po	-

List 46: TUA7 (Napuka Island, Tuamotu). Reported by Audran (1919: 39, 1929) (*53*, *65*). The final part of the list deserves comment. “Tinai po” in the 29th position is equivalent to “Tinai ki te po” in other lists and we discarded it (see TUA3); “tamu te aiahi” may also be a descriptive sentence rather than a night’s name, given that *ahiahi* means ‘evening, late afternoon’. However, it must be noticed that excluding these two items leaves us with a list of 29 nights. Finally, “O Hiro” appears in the last (31st) position, but probably it was the first night of the month.

	Original	Normalized
1	O Hoata	so#ʔata
2	E tahi-hania	tahi_maseʔa/samia
3	E roto-hania	roto_maseʔa/samia
4	Fakaoti-hania	faka3#ʔoti_maseʔa/samia
5	E tahi korekore	tahi_kore#kore
6	E roto korekore	roto_kore#kore
7	Fakaoti korekore	faka3#ʔoti_kore#kore
8	Ovari	ʔari/vari
9	O Tamatea	tamatea
10	O Hua	fua
11	O Mahuru	maharu
12	O Maitu	maitu
13	O Hotu	fotu/fotu
14	O rangi [Marangi?]	raŋi
15	O turu	turu
16	E tahi korekore	tahi_kore#kore
17	E roto korekore	roto_kore#kore
18	Fakaoti korekore	faka3#ʔoti_kore#kore
19	E tahi tagaroa	tahi_taŋaroa
20	E roto tagaroa	roto_taŋaroa
21	Fakaoti tagaroa	faka3#ʔoti_taŋaroa
22	E tahi rakau	tahi_raakau
23	E roto rakau	roto_raakau
24	Fakaoti rakau	faka3#ʔoti_raakau
25	E tiketike	tika/tikatika
26	O Rogo nui	roŋo#nui6
27	O Rogo maori	roŋo#mauri
28	Mauri Kero	mauri#kero
29	Tinai-po	-
30	Tamu te a[h]iahi	-
31	O Hiro	firo

List 47: TUA8 (Marokau, Tuamotu). Given to Audran (1929) by Ralph Teragireva, "from Marokau" (*53*). As per above, we did not count "Tinai ki te po" as a night (see TUA3).

	Original	Normalized
1	Otipi	tipi
2	Ohiro	firo
3	Ohoata	so#ʔata
4	O hania tahi	maseʔa/samia_tahi
5	O hania roto	maseʔa/samia_roto
6	O hania hakaoti	maseʔa/samia_faka3#ʔoti
7	Nakore tahi	kore_tahi
8	Nakore roto	kore_roto
9	Nakore hakaoti	kore_faka3#ʔoti
10	O Tamatea	tamatea
11	O vari	ʔari/vari
12	Ohuna	funa
13	Ohua	fua
14	Omaharu	maharu
15	O maitu	maitu
16	O maragi	maa2#raŋi
17	O hotu	fotu/fotu
18	O toru [Turu] (Pahoro)	turu
19	Hania-tahi	maseʔa/samia_tahi
20	Hania-roto	maseʔa/samia_roto
21	Hania hakaoti	maseʔa/samia_faka3#ʔoti
22	Kore tahi	kore_tahi
23	Kore roto	kore_roto
24	Kore-hakaoti	kore_faka3#ʔoti
25	Tagaroa tahi	taŋaroa_tahi
26	Tagaroa roto	taŋaroa_roto
27	Tagaroa hakaoti	taŋaroa_faka3#ʔoti
28	Rogo-nui	roŋo#nui6
29	Rogo-mauri	roŋo#mauri
30	Mauri Kero	mauri#kero
31	Tinai ki te po	-

List 48: TUA9 (Raroia, Tuamotu). Collected in Danielsson (1956: 187-8) (*66*).

	Original	Normalized
1	Hiro	firo
2	Hoata	so#ʔata
3	Hania tahi	maseʔa/samia_tahi
4	Hania roto	maseʔa/samia_roto
5	Hania fakaoti	maseʔa/samia_faka3#ʔoti
6	Korekore tahi	kore#kore_tahi
7	Korekore roto	kore#kore_roto
8	Korekore fakaoti	kore#kore_faka3#ʔoti
9	Tamatea	tamatea
10	Huna	funa
11	Rapu	rapu
12	Maharu	maharu
13	Hua	fua
14	Māitu	maitu
15	Hotu	fotu/fotu
16	Mārangi	maa2#raŋi
17	Penu	penu
18	Rākau tahi	raakau_tahi
19	Rākau roto	raakau_roto
20	Rākau fakaoti	raakau_faka3#ʔoti
21	Korekore tahi	kore#kore_tahi
22	Korekore roto	kore#kore_roto
23	Korekore fakaoti	kore#kore_faka3#ʔoti
24	Taaiva tahi	taaiva_tahi
25	Taaiva roto	taaiva_roto
26	Taaiva fakaoti	taaiva_faka3#ʔoti
27	Tikatika	tika/tikatika
28	Rogo nui	roŋo#nui6
29	Rogo mauri	roŋo#mauri
30	Mauri Kero	mauri#kero

S1B. Index of forms

Form	(Proto-)Language of normalized form and gloss	Reference
ʔaniwa	Marquesan; perhaps *ʔaniwa* < PEP **Kaniwa* ‘The Milky Way’(?)	https://pollex.eva.mpg.de/entry/kaniwa/
ʔari/howaru	Moriori Howaru (possibly a misprint for Howari?) most likely reflects **O Ari*, though we were conservative and treated it as a specific variant	See ‘ʔari/ʔari’ below
ʔari/vari	Tuamotuan and Rarotongan variant of *Ari*, most probably due to reinterpretation, after the word *vari* ‘menstrual blood’ (< **wari*)	https://pollex.eva.mpg.de/entry/wari/
ʔari/ʔari	PEP **ʔari*, probably identical with **ʔari* ‘clearly visible’; the Rennellese language has the derivative *ʔagiʔagi* as the name of the first night of the lunar month (new moon), but this may be an independent innovation rather than a trait shared by the Rapa Nui lists, where O Ari is also the first or one of the first nights (notice the reduplicated form, not seen in the Eastern Polynesian calendars); the Rennell Island used a ‘numbering’ type of the lunar calendar	https://pollex.eva.mpg.de/entry/qali1b/ https://pollex.eva.mpg.de/entry/qali.1a/
ʔata	PEP **ʔata* ‘reflection’	https://pollex.eva.mpg.de/entry/qata/ See also ‘faka1’ below
ʔavaiki	Rarotongan *ʔavaiki* (< PEP **sawaiki*), mythological name of Maori homeland	https://pollex.eva.mpg.de/entry/sawaiki/
ʔoti	PEP **ʔoti* ‘end(ed), finish(ed), complete(d)’	https://pollex.eva.mpg.de/entry/qoti.a/
ʔuri	Rapanui *ʔuri* ‘black, dark’	https://pollex.eva.mpg.de/entry/quli.2/
a	Rapanui particle of uncertain meaning in its context	
aio	Maori *āio*, perhaps identical with *āio* ‘be calm, at peace’	https://maoridictionary.co.nz/word/7346 https://maoridictionary.co.nz/word/122
aivaku	Marquesan; obscure and perhaps a misprint	
ariki	Maori ‘chief’ (< PEP **ʔariki*)	https://pollex.eva.mpg.de/entry/qariki/
atiati	Marquesan *atiati* (meaning and etymology obscure)	Dordillon (1931: 112) (*55*)
atua	PEP **Atua*, perhaps from **ʔAtua* ‘deity’	https://pollex.eva.mpg.de/entry/atua/ https://pollex.eva.mpg.de/entry/qatua/
aurei	Maori *aurei*; there is an identical word in Maori that means ‘cloak pins of ivory or greenstone’	https://maoridictionary.co.nz/word/15515
faa	PEP **faa* ‘four’	https://pollex.eva.mpg.de/entry/faa.1/
faka	PEP **faka*, causative prefix	https://pollex.eva.mpg.de/entry/faka-.1/
faka1	Reflex of the causative prefix as found with the forms ‘ʔata’ and ‘soʔata’ (see below) in Marquesan *Tu-hakahoata* and Tuamotuan *Fakaata*	https://pollex.eva.mpg.de/entry/faka-ata.b/ See also ‘so’ below

faka2	Reflex of the causative prefix as found with ‘pau’ in Marquesan *hakapao* ~ *hakapau* and Maori *whakapau* (a qualifier of night series)	https://maoridictionary.co.nz/word/9740 See also ‘pau’ below
faka3	Reflex of the causative prefix as found with ‘ʔoti’ in various lists and languages (a qualifier of night series)	https://pollex.eva.mpg.de/entry/faka-otib/ See also ‘ʔoti’ above
faka4	Reflex of the causative prefix as found with ‘piri’ in Maori *whakapiri* (used with the *Korekore* series in MAO1)	https://maoridictionary.co.nz/word/9751 See also ‘piri’ below
hetaumaro	Manihiki *Whetau-maro* and Rakahanga *etau maro*, of uncertain meaning	
firo	PEP **Firo*, perhaps the name of the god of misfortune (Tuamotuan Hiro, Maori Whiro, etc.)	https://pollex.eva.mpg.de/entry/firo.2/ Craig (1989: 121) (*14*)
fiti	PEP **fiti* ‘rise (celestial bodies)’	https://pollex.eva.mpg.de/entry/fiti.1b/
fitu	PEP **fitu* ‘seven’	https://pollex.eva.mpg.de/entry/fitu/
fotu (fotu/fotu)	PEP **Fotu*, perhaps ‘appear, emerge’	https://pollex.eva.mpg.de/entry/fotu.4/ https://pollex.eva.mpg.de/entry/fotu.1/
fonu (fotu/fonu)	Marquesan (*h*)*onu*: a variant of (*H*)*otu* (see previous) and homophonous with *honu* ‘turtle’ (< *PEP *fonu*); perhaps the result of reinterpretation	https://pollex.eva.mpg.de/entry/fonu.2/
fua	PEP **Fua*, perhaps ‘to bloom’	https://pollex.eva.mpg.de/entry/fua.3c/ https://pollex.eva.mpg.de/entry/fua.3a/
funa	PEP **Funa*, perhaps ‘hidden’	https://pollex.eva.mpg.de/entry/funa.2/ https://pollex.eva.mpg.de/entry/funa.1a/
hahani	Maori *hahani* (‘disparaging; scorn’?)	https://maoridictionary.co.nz/word/10713
haru	Rapanui *haru*, of obscure meaning (very likely a ‘corrupted’ form of *Maharu*?), unlikely to be identical with modern Rapanui *haru* ‘grab’	
hava	Marquesan *hava*, whose meaning is unclear in its compounded form with *Tū*	
heeheeia	Marquesan *Hee hee ia*, of obscure meaning and found only in one list (MRQ8); it is likely to be a misprint	
heŋa	Rapanui *heŋa* ‘bright(ness); to dawn’ (cf. also *heŋaheŋa* ‘reddish’)	https://pollex.eva.mpg.de/entry/sega.2/
hiva	Marquesan *hiva*, whose meaning is unclear in its compounded form with *Tū* (and *Pū*?) (see below); it might, however, be related to *hivahiva* ‘(silver-)gray’ < **siwa* ‘black,’ if it is a description of the waning moon	https://pollex.eva.mpg.de/entry/siwa.2/
huka	Unknown; probably a misprint	
inaira	Rapanui *Ina-ira*, literally ‘it is not there’	Horley *et al.* (2018) (*67*)
kaha	Mangarevan *kaha*; its meaning as a qualifier of *Korekore* is unclear	
kai	Maori *kai* ‘eat’	https://maoridictionary.co.nz/word/1894
kakere	Tuamotuan form found only in TUA5 and of unclear meaning; perhaps related to *kero* (see below)?	

kani	Maori *kani*, of unclear meaning in this context	
kero	PEP **kero* 'die, dying'	https://pollex.eva.mpg.de/entry/kelo1b/
kiokio	Maori *kiokio*, probably 'shade' in this context (it is the name one of the last nights of the lunar cycle)	Tregear (1891: 149) (*37*)
ko	Rapanui; repeated part of the morph *kore* in the partially reduplicated *Kokore* (see next)	
kore	PEP **kore* 'without, lacking,' part of the *(*Kore*)*kore* night series	https://pollex.eva.mpg.de/entry/kore-kore/ https://pollex.eva.mpg.de/entry/kore/
kumea	Marquesan form of uncertain meaning (although it probably contains the same morpheme as *Taukume*, see below)	
ma1	Tuamotu particle in the name *Rongo-ma-titiro*, probably with an inclusive sense ('and, plus, with')	https://pollex.eva.mpg.de/entry/ma.4/
ma2	Marquesan particle in *Honumaakau* (*Honu-ma-ʔĀkau*), which probably denotes the joining of the names *Honu* (*Hotu* < **Fotu*) and *ʔĀkau* (**Raʔakau*) in a single night; linguistically, it is cognate with the previous, but its use here is trivial and unrelated	https://pollex.eva.mpg.de/entry/ma.4/
maa	PEP *-*maa* 'numeral conjunction', joined to PEP **Maseʔa*	https://pollex.eva.mpg.de/entry/maa.1/
maa2	PEP **maa*; stative prefix indicating quality or state; used in the compound **Maa-raŋi*; meaning unclear; we separated it to distinguish between the Hawaiian variant forms *Mālani* ('maa2#raŋi') and *Māhealani* ('maseʔa2#raŋi')	https://pollex.eva.mpg.de/entry/ma-.1/
maharu	PEP **maharu* or similar	https://pollex.eva.mpg.de/entry/maharu/
mai	Maori *mai*, whose meaning in this context is unclear and may be a misprint for *nui*	
maitu	Raivavae, Rarotongan, Tahitian and Tuamotuan *Maitu*, probably identical with *māitu* 'a god, spirit'	https://pollex.eva.mpg.de/entry/maitu/
mararo	Tuamotuan *mararo*, which is synchronically obscure, but probably cognate with Hawaiian *malalo* 'below, underneath'	
maseʔa	See below maseʔa/maseʔa and maseʔa/samia	
maseʔa2	Hawaiian *māhea* 'hazy (moonlight);' this notation distinguishes the Hawaiian night *Mālani* ('maa2#raŋi') ~ *Māhealani* ('maseʔa2#raŋi') from the widespread name of the series **Maseʔa-maa* ~ **Samia*(-*maa*) (see next)	
maseʔa/maseʔa	PEP **Ma*(*a*)*seʔa*, probably 'faintly perceptible'	https://pollex.eva.mpg.de/entry/ma-hea/ https://pollex.eva.mpg.de/entry/ma-seqa/
maseʔa/samia	**Samia*(-*maa*), whence Tahitian *Hamiama*, Tuamotuan *Hania*, Tongarevan *Samia*, etc., probably originated as a phonological	

	variant of **Maseʔa*(-*maa*), through metathesis[1]	
matanui3	Maori *mata nui* 'big eye' used as a qualifier of *Ari* in one list	https://maoridictionary.co.nz/word/3752 https://pollex.eva.mpg.de/entry/nui.1/
mate1	Maori *mate* '(be) dead,' used in an extended name of the night Atua, which is not found at the end of the lunar cycle (hence this is treated as not cognate with mate2; see next)	https://pollex.eva.mpg.de/entry/mate.1a/ https://maoridictionary.co.nz/word/3845
mate2	PEP **mate* '(be) dead,' used with **Mauri* and **Roŋo* nights at the end of several lists, alluding to the end of the lunar cycle	https://pollex.eva.mpg.de/entry/mate.1a/
matofi	PEP **Matofi*, probably 'split in two,' referring to the appearance of the moon in second half of its cycle; it is also found in some numbering calendars from West Polynesia (S1D) and must be an archaism	https://pollex.eva.mpg.de/entry/ma-tofi.b/ https://pollex.eva.mpg.de/entry/ma-tofi.a/ Tregear (1891: 523) (*37*)
matua	Rapanui *matua*, perhaps *matuʔa* 'parent' or 'main, principal'	https://pollex.eva.mpg.de/entry/ma-tuqa.c/ https://pollex.eva.mpg.de/entry/ma-tuqa.b/
maure	PEP **Maure*, of uncertain meaning	https://pollex.eva.mpg.de/entry/maure/
mauri	PEP **Mauri*	https://pollex.eva.mpg.de/entry/mauri/
mawete	Maori, perhaps *mawete* 'loosened, released'	https://maoridictionary.co.nz/word/43014
mea	Rapanui *mea* 'light red'	https://pollex.eva.mpg.de/entry/mea.1a/
muri	PEP **muri* 'behind, after'	https://pollex.eva.mpg.de/entry/muri.1a/
mutu	PEP **mutu* 'end, ended'	https://pollex.eva.mpg.de/entry/mutu.1b/ https://pollex.eva.mpg.de/entry/mutu1a/
muʔa	PEP **muʔa* 'front, before'	https://pollex.eva.mpg.de/entry/muqa.a/
nonihape	Maori *Nonihape*, of uncertain meaning	
nui1	Mangarevan and Marquesan *nui* 'big' < PEP **nui* used with **Tuu* at the beginning of some lists	https://pollex.eva.mpg.de/entry/nui.1/
nui2	Maori *nui* 'big' < PEP **nui* used with *Tamatea* in some lists; trivial and not related to previous	https://pollex.eva.mpg.de/entry/nui.1/
nui3	Marquesan *nui* 'big' < PEP **nui* used as a qualifier of *Hotu*; trivial and not related to previous	https://pollex.eva.mpg.de/entry/nui.1/

[1] Stimson (1930: 276) identified as cognates MRQ *Maheama*, MGV *Ma*[*ʔ*]*ema*, TAH *Hamiāma*, Rurutu (Austral islands) *ʔamia*, TGV *Samia*, TUA *Hania* ~ *Hanīa* (for this last form see Stimson 1964: 117) (21, *64*). He assumed the following development: *Samia* > *Hamia* > *Hamia-ma*(*-tahi*, etc.) > *Mahiama* > *Maheama* / *Ma*[*ʔ*]*ema*. Yet this leaves the etymology of the name unexplained, as most forms are opaque. We suggest that the path was the opposite. The starting form is Proto-East Polynesian **ma-seʔa* '(be) perceptible' (POLLEX) (14), originating the structure **maseʔa*(*-maa*)-NUMERAL, where **maa* is a numeral connective particle that became lexicalized and fossilized as part of the names of nights only in some calendars. Thus, **Maseʔa*(*-maa*)- > MRQ *Mahea-ma* / MGV *Ma*[*ʔ*]*ema* (with **s* > *h*) but also > **Same*[*ʔ*]*a*(*-ma*), with metathesis (and followed by the irregular shift *e* > *i*) > TGV *Samia*, TAH *Hamiama* (with *e* > *i*), and TUA **Hamia* > *Hania* (irregular *m* > *n*). The etymology is supported by the semantics of Hawaiian *māhea* 'hazy (moonlight)', Rapanui *maʔeha* 'brightness; lighten/brighten up', and Tahitian *maheahea* 'turn pale; fade', keeping in mind that this series of nocturnal names referred to some of the first nights after the new moon, when the latter is barely visible.

nui4	Maori *nui* 'big' < PEP **nui* used as a qualifier of *Rākau*; trivial and not related to previous	https://pollex.eva.mpg.de/entry/nui.1/
nui5	Rapanui *nui* 'big' < PEP **nui* used as a qualifier of *Mauri*; trivial and not related to previous	https://pollex.eva.mpg.de/entry/nui.1/
nui6	PEP **nui* used as a qualifier of **Roŋo* trivial and probably not related to previous	https://pollex.eva.mpg.de/entry/nui.1/
nuku	Maori *nuku*, meaning 'wide extent' or 'Earth'	https://maoridictionary.co.nz/word/4494
ŋa	PEP *-*ŋa* 'nominalizing suffix'	https://pollex.eva.mpg.de/entry/-ga/
ŋa1	-*ŋa* 'nominalizing suffix' (< PEP *-*ŋa*) > in Tuamotuan *tahiŋa* 'oneness'	See previous
ŋa2	-*ŋa* 'nominalizing suffix' (< PEP *-*ŋa*) > in Tuamotuan *rotoŋa* 'inside, interior'	See previous
ŋa3	-*ŋa* 'nominalizing suffix' (< PEP *-*ŋa*) > in Tuamotuan *rotoŋa* 'inside, interior'	See previous
ŋa4	-*ŋa* 'nominalizing suffix' (< PEP *-*ŋa*) > in Manahiki-Rakahanga *mutuŋa* 'ending'	See previous
ŋana	Maori *ngana*, perhaps the non-reduplicated form of *ngangana* 'red, glowing'(?)	https://pollex.eva.mpg.de/entry/gana.1/ https://maoridictionary.co.nz/word/4553
ohau	Obscure modern Rapanui form	
ohea	Obscure modern Rapanui form	
ohuri	Obscure modern Rapanui form	
oika/oika	Maori *O*(*h*)*ika*¸ variant of next, of obscure meaning; if it is the conservative form, it might reflect **O Hika*, from PEP **s*/*hika* 'female genitals'	https://pollex.eva.mpg.de/entry/sika.3/
oika/oike	Maori *Oike*, of obscure meaning (see previous)	
okoro	Maori *Okoro* (also *Akoro*, in one list), of obscure meaning	https://maoridictionary.co.nz/word/4738
ono	PEP **ono* 'six'	https://pollex.eva.mpg.de/entry/ono/
otama	Maori *Otama* of obscure meaning (perhaps a corrupted form of *Tamatea*?)	
oue	Maori *Ōue*, of uncertain meaning	
pa	Maori and most probably *pā*; the meaning in the context of the calendar is uncertain	
pahoro	Tuamotuan *pahoro* 'a kind of parrotfish (*Scaridae forsteri*),' likely denoting a fish that could be found in the night of Turu, as this is a variant of that night's name	https://pollex.eva.mpg.de/entry/pahoro/
pau	PEP **pau* 'finished, ended'	https://pollex.eva.mpg.de/entry/pau1c/
penu	Tuamotuan *Penu*, whose meaning is unclear	
pewa	Maori *pewa* 'to be a new moon,' which is the name of the first night in one Maori list (MAO_WAI)	https://maoridictionary.co.nz/word/5536
piri	Maori *piri* 'stick, keep close'	https://maoridictionary.co.nz/word/5640
poipoi	Marquesan *poipoi*, of uncertain meaning; it may be a misprint for 'Koekoe'	

popo	Modern Rapanui form of uncertain meaning; it is reminiscent of Rapanui *pō* 'night' and *popohaga* '(to) dawn'	
puu	Probably Marquesan *pū* (*puu*) in *Puhiwa*, of uncertain meaning; a misprint is possible, as this is found in only one list (MRQ1) and three other Marquesan calendars have *Tuhiva* (MRQ2, MRQ5, MRQ7)	
raakau	PEP **Raakau*, probably identical with **raʔakau* 'tree, plant, wood'	https://pollex.eva.mpg.de/entry/raakau.1/ https://pollex.eva.mpg.de/entry/raqa-kau.a/ https://pollex.eva.mpg.de/entry/raqa-kaub/
raŋa	Rapanui *raŋa*, perhaps 'swarming (fish)'	https://pollex.eva.mpg.de/entry/laga.1b/
raŋi	PEP **raŋi*, probably 'sky,' in the night **Māraŋi* (*maa2#raŋi) and its variant Hawaiian *Māhealani* (*maseʔa2#raŋi)	https://pollex.eva.mpg.de/entry/lagi.2/
rapu	Rarotongan, Tahitian and Tuamotuan form of uncertain meaning; according to Stimson, Tahitian *rapu* refers to the "striated" condition of roe when it "is mature and ready to be deposited"	Stimson (1928: 336, n. 26) (12)
rari	Tuamotuan *rari* 'one'	Stimson (1964: 434) (*64*)
rawea	Maori *rawea*, of obscure meaning in this context	https://maoridictionary.co.nz/word/6653
rima	PEP **rima* 'five'	https://pollex.eva.mpg.de/entry/lima.a/
riro	Mangarevan *riro* 'gone'	https://pollex.eva.mpg.de/entry/lilo/ https://pollex.eva.mpg.de/entry/riro/
roa	Maori *roa* 'long'	https://maoridictionary.co.nz/word/6850
roŋo	PEP **roŋo*, probably identical with the name of one of the main Polynesian deities	https://pollex.eva.mpg.de/entry/rogo.2/ Craig (1989: 142-3, 231-2) (14)
roto	PEP **roto* 'inside'	https://pollex.eva.mpg.de/entry/loto.a/
rua	PEP **rua* 'two'	https://pollex.eva.mpg.de/entry/rua/
so	Lexicalized(?) prefix in PEP **soʔata* 'bright,' where we recognize PEP **ʔata* 'reflection;' we have notated 'so' separately because **ʔata* occurs without **so* in Tuamotuan *Fakaata* (TUA1), probably **faka-ʔata* 'reflect(ion)' (see faka1 below)	https://pollex.eva.mpg.de/entry/soata.3/ https://pollex.eva.mpg.de/entry/soqata/ https://pollex.eva.mpg.de/entry/faka-ata.b/
taaiva	Tuamotuan *Tāiva*, probably 'forsaken (for a long time)'	Stimson (1964: 485-6) (*64*)
taane	PEP **Taane*, a divine name (compare PEP **Taʔane* 'male')	https://pollex.eva.mpg.de/entry/taane/ https://pollex.eva.mpg.de/entry/taqane/ Craig (1989: 101-2) (14)
tahi	PEP **tahi* 'one'	https://pollex.eva.mpg.de/entry/tasi.1/
tai	Rapanui *tai*, perhaps 'ocean'	https://pollex.eva.mpg.de/entry/tahi.1/
takatakapuutea	Maori *Takatakapūtea*, a compound of uncertain meaning	https://maoridictionary.co.nz/word/46543
tamatea	Form attested in Maori, Rarotongan, Tahitian, Tongarevan, and Tuamotuan; the proto-form may be reconstructed as Proto-Tahitic (POLLEX) or 'Proto-Eastern-Polynesian Proximal', depending on the	https://pollex.eva.mpg.de/entry/tamatea/ Craig (1989: 257) (14)

	classification endorsed; in Maori and Tahitian it is also a divine name	
tanahau	Marquesan *Tana hau* is likely a misprint for *Tanaoa* ‘Tangaroa’ (see next), but we were conservative and transcribed it as found in the literature	
taŋaroa	PEP **Taŋaroa*, also the name of one of the main Polynesian deities	https://pollex.eva.mpg.de/entry/tagaroa.b/ https://pollex.eva.mpg.de/entry/tagaloa.a/
tapume	Rapanui *tapume*; it lacks etymology (and it is suspiciously similar to Marquesan *taukume*; see next)	
taukume	Marquesan taukume; see previous and ‘kume’	
tea1	Rapanui *tea* ‘white,’ used only in one list (RPN4), and only as the adjective following *haru* and *popo*	https://pollex.eva.mpg.de/entry/tea/
tea2	Tahitian *tea* ‘white,’ used as adjective modifying the name of the night *Turu*	https://pollex.eva.mpg.de/entry/tea/
tea3	Marquesan *tea* ‘white,’ used as a self-standing name in some lists	https://pollex.eva.mpg.de/entry/tea/
tia	Tuamotuan *tia*, added to Rongo-mauri in TUA5; perhaps *tia* ‘to shine, glitter’	Stimson (1964: 526) (*64*)
tika/tika	Tuamotuan *tika*, probably ‘to stand up, rise’	
tika/tikatika	Reduplicated variant of previous, possibly with the sense of ‘very elevated’	Cf. Stimson (1964: 532) (*64*)
tipi	Tuamotuan *Tipi*, perhaps meaning ‘glide along the surface’(?)	https://pollex.eva.mpg.de/entry/tipi.3/
tireo/tere	Moriori *tere*, a variant of *Tireo* (see below)	
tireo/tirea	Maori *Tirea*, a variant of *Tireo* (see next)	https://pollex.eva.mpg.de/entry/tireo/ https://maoridictionary.co.nz/word/8186
tireo/tireo	PEP **Ti*(*i*)*reo*, of obscure meaning	https://pollex.eva.mpg.de/entry/tireo/
tireo/tiroe	Rarotongan (probably borrowed into Tongarevan) *Tiroe*, a variant of *Tireo* (see previous)	https://pollex.eva.mpg.de/entry/tireo/
titiro	Tuamotuan *titiro*, of uncertain meaning	
toru	PEP **toru* ‘three’	https://pollex.eva.mpg.de/entry/tolu/
turu	PEP **turu*, whose etymological meaning (as a night’s name) is uncertain	https://pollex.eva.mpg.de/entry/turu/
tuu	We used the reconstruction given in POLLEX, **Tuu*, which is identical with the name of the god of creation; however, it likely (also) reflects PEP **tuʔu* ‘(be) upright’ alluding to the moon’s rise; this is the name of the first night(s) in several lists	https://pollex.eva.mpg.de/entry/tuu3/ Craig (1989: 121) (14) https://pollex.eva.mpg.de/entry/tuqu.1/
tuu2	Marquesa *tuu* in *Tuu Hiva*; even if this is from PEP **tuʔu* ‘(be) upright’, we treated this form as non-identical with the previous, because (1) its position in the list (24th) makes it unlikely to be related with **Tuu* as found at the beginning of many lists and (2) its relationship with *Puhiwa* is unclear.	

tvkirau/takirau	Maori *Takirau*, whose meaning is obscure; clearly equivalent to Marquesan *Tohi*[*ʔ*]*au*, though the phonological correspondence is irregular; the proto-form is likely **t*V*k*/*hirau*, which we annotated as 'tvkirau'	https://maoridictionary.co.nz/word/7283
tvkirau/tohiʔau	See previous	
vaitu	Tuamotuan *Vaitu*; according to Stimson (1964: 598) it should be a variant of *aitu* 'ghost', also a divine name, but this is phonologically irregular, and the term is suspiciously similar to Vaitu, the name of a month	Stimson (1964: 46, 598–9) (*64*) https://pollex.eva.mpg.de/entry/qaitu/
vaka	Marquesan *vaka*, perhaps not identical with *vaka* 'canoe,' which in the southern dialect is *vaʔa* (< **waka*)	https://pollex.eva.mpg.de/entry/waka.a/
vehi	Marquesan *vehi* (cognate with Mangarevan *veʔi*); it is reminiscent of Hawaiian *wehi* 'dark' and PEP **wesi* 'disturbed' and may therefore allude to the appearance of the moon in its waning phase	https://pollex.eva.mpg.de/entry/vehi/ https://pollex.eva.mpg.de/entry/fesi.2/
waheŋa	PEP **wahe-ŋa* > Marquesan *vaveka* ~ *vavena* 'middle'	https://pollex.eva.mpg.de/entry/wahe-ga/
waru	PEP **waru* 'eight'	https://pollex.eva.mpg.de/entry/walu.1/
whakahaehae	Maori *whakahaehae* ('ghostly, haunted'?), modifier of *Atua* in one list	https://maoridictionary.co.nz/word/9397 https://pollex.eva.mpg.de/entry/sae.2/
whakatehe	Maori *whakatehe*, used only in one list (MAO2) as a modifier of the Korekore series; it is formed by the causative prefix *whaka* (< **faka*) + *tehe*, which in this context is obscure	
whenua	Probably Maori *whenua* 'land'	https://maoridictionary.co.nz/word/10256 https://pollex.eva.mpg.de/entry/fenua.a/
whitikiraua	Maori compound word whose exact meaning (as a night's name) is unclear	
whiwhia	Maori word of uncertain meaning (perhaps a form of *whiwhi* 'entangle' or 'acquire'?)	https://pollex.eva.mpg.de/entry/fifi.2a/ https://maoridictionary.co.nz/word/10361

S1C. Description of calendrical lists from West Polynesia and other areas

Here we present data on the ‘nights of the moon’ in West Polynesia and additional areas where other Polynesian languages are spoken and discuss how these compare with the East-Polynesian lists.

Mugaba/Rennell (Rennell and Bellona, Solomon Islands): Reported in Elbert and Monberg (1965: 105-6, after Taupongi [1961] and Jotham Togaka of Labagu [1962]) (*68*). The reduplicated morpheme in the name of the first night, *ʔAngiʔangi* (< **ʔariʔari*) is cognate with Proto-East-Polynesian **ʔari*. However, the night's position is not consistent with the East-Polynesian derivatives of **ʔari*, except in the Rapanui lists, where it appears at the start of the lunar cycle. There are no non-trivial similarities with the East-Polynesian lists. Number words are used extensively (2nd-10th and 15th-28th nights).

1	*ʔAngiʔangi*
2	*Hakahaa*
3	*Hakangima*
4	*Hakaono*
5	*Hakahitu*
6	*Hakabangu*
7	*Hakaiba*
8	*Hakaangahungu*
9	*Tuuma'a tasi*
10	*Tuuma'a ngua*
11	*Malanga*
12	*Hakasumaagie*
13	*Kaukau*
14	*Mangiko saasaa*
15	*Mangiko ngua*
16	*Hakatongu*
17	*Hakahaa*
18	*Hakangima*
19	*Hakaono*
20	*Hakahitu*
21	*Hakabangu*
22	*Hakaiba*
23	*Hakaangahungu*
24	*Poo ngima*
25	*Poo haa*
26	*Poo tongu*
27	*Poo ngua*
28	*Poo tasi*
29	*Takoto tugitugi*
30	*Ngangue*

Mungiki/Bellona (Rennell and Bellona, Solomon Islands): Reported in Elbert and Monberg (1965: 105-6, after Taupongi [1961]) and Elbert (1975) (*68*, *69*).

1	*ʔAngiʔangi*
2	*Hakahaa*
3	*Hakangima*
4	*Hakaono*
5	*Hakahitu*
6	*Hakabangu*
7	*Hakaiba*
8	*Hakaangahungu*
9	*Tuuma'a tasi*
10	*Tuuma'a ngua*
11	*Malango*
12	*Kaukau*
13	*Mangiko saasaa*
14	*Mangiko ngua*
15	*Hakatongu*
16	*Hakahaa*
17	*Hakangima*
18	*Hakaono*
19	*Hakahitu*
20	*Hakabangu*
21	*Hakaiba*
22	*Hakaangahungu*
23	*Poo ngima*
24	*Poo haa*
25	*Poo tongu*
26	*Poo ngua*
27	*Poo tasi*
28	*Asongango*
29	*Nganguengue*

Nanumea (Tuvalu): From the entries in *Nanumea Lexicon*, published by Ranby (1980) (*70*), we can only piece together an incomplete list. Reconstructed entries are marked with an asterisk. There are no non-trivial similarities with the East-Polynesian lists. Number words are used extensively, up to *fitu* for 'seven'.

1	*kila te maahina* (New Moon)
2	*maalua*
3	*maatolu*
4	*maafaa te maahina*
5	*maalima* *(*te maahina*)
6	*maaono* *(*te maahina*)
7	*maafitu* *(*te maahina*) / *tuu tonu te maahina*
8	**maatahi te tai*
9	**maalua te tai*
10	**maatolu te tai*
11	*maafaa te tai*
12	**maalima te tai*
13	**maaono te tai*
14	*maafitu te tai / katoa te maahina / hulu te maahina* (Full Moon)
15	*ko matuku te tai*
16	*ko poolua te matukuga o te tai*
17	**ko pootolu te matukuga o te tai*
18	**ko pootolu te matukuga o te tai*
19	**ko poolima te matukuga o te tai*
20	**ko pooono te matukuga o te tai*
21	**ko poofitu te matukuga o te tai*
22	*poo tahi te ologa o te tai*
23	*poo lua te ologa o te tai*
24	*poo tolu te ologa o te tai*
25	*poo faa te ologa o te tai*
26	*poo lima te ologa o te tai*
27	*poo ono te ologa o te tai*
28	*poo fitu te ologa o te tai*
29	?

Niue: From the entries in Sperlich's dictionary of the Niue language (1997) (*71*), we learn only the following about the local names of the lunar nights:

- *Matahila* is the "night of the new moon". There are other expressions: *mahina pula mui*, though this seems to refer to the first quarter as a whole, and *mahina pula foou*
- The full moon is called *mahina kau* or *mahina kaulapalapa*. After the full moon, the nights are counted as follows: *pōuli taha* "first night after the full moon", and *pōuli ua* "second night after the full moon".
- The last night of the last quarter is called *hopomate* (the last quarter as whole is apparently called *mahina hopomate*).

Pukapuka (northern Cook): Reported in E. and P. Beaglehole (1938: 347) (*72*). The second night, *Po wakaata* ("Making shadows of the night"), contains a cognate of Proto-East-Polynesian **fakaʔata* 'cast a reflection/shadow', which is attested in TUA1 and related to *(*faka*)*soʔata*). The latter appears at the start of many East-Polynesian lists. As the list contains 31 nights, we suggest that the sentence labeled as "1" by the authors refers to the start of the lunar cycle rather than naming an independent night. Number words are used extensively, though not beyond *lima* 'five'. Much of the vocabulary reflects other names or descriptors of nights from the West Polynesian tradition but expressed here in longer phrases.

1	*Koa makatia e te la*: (the moon is) thrown out by the sun
2	*Po wakaata*: making shadows of the night
3	*Po pula*: the night is spotted
4	*Po lua*: the second night
5	*Po tolu*: the third night
6	*Po wa*: the fourth night
7	*Po lima*: the fifth night
8	*Koa tutu te maina*: the moon is erect , or stands high
9	*Koa itu wenua*: the land is divided into two (by the moon?)
10	*Koa tu mai loa ki lunga te maina* : the moon is on high
11	*Koa ngalue te maina*: the moon moves (higher)
12	*Koa wolo te maina*: the moon is big
13	*Koa wolo loa te maina*: the moon is very big
14	*Koa momona te maina*: the moon is fat
15	*Koa maliko te maina*: the moon shines or glimmers
16	*Koa wakaulu te maina*: the moon is overhead
17	*Koa yengie te maina*: ? (refers to the night when the crabs mate)
18	*Te lama yengi*: the yellow light , or the yellow torch
19	*Te uluaki kau pouli*: the first-born of the group of dark nights
20	*Wakatai*: to make the first dark night
21	*Wakalua*: to make the second dark night
22	*Wakatolu*: to make the third dark night
23	*Lama wakatau*: the (time for) torch fishing is equal (to the length of moonlight)
24	*Koa loa te po*: the night is long
25	*Koa ngalue ki ngake*: (moon) moves to the east
26	*Koa walai loa ki ngake*: (moon) tilts well to the east
27	*Koa kauliliki loa*: (the moon) is very small
28	*Koa wakatau wenake ma te ulu o te ata matua*: (moon) rises at the same time as the darkness before dawn
29	*Koa wakatau wenake ma te ata maoa*: (the moon) rises just at the first streaks of gray dawn
30	*Koa wenake ma te ata kena*: (the moon) rises just at gray dawn
31	*Koa wakatau wenake ma te la*: (the moon) rises with the sun

Samoa: Reported by an anonymous member of the Samoan Society (Anonymous 1928: 239) (*73*). Most of the list uses counting expressions.

1	*Fakatahi*
2	*Fakalua*
3	*Fakatolu*
4	*Fakafa*
5	*Fakalima*
6	*Fakaono*
7	*Fakafitu*
8	*Fakavalu*
9	*Fakaiva*
10	*Magafulu*
11	*Fakatasi*
12	*Fakalua*
13	*Utua*
14	*Malama*
15	*Fakatahi*
16	*Fakalua*
17	*Fakafulu*
18	*Fakafa*
19	*Fakalima*
20	*Fakatutupu*
21	*Magafulu*
22	*Poiva*
23	*Povalu*
24	*Pofitu*
25	*Poono*
26	*Polima*
27	*Fanouluata*
28	*Fanolotoata*
29	*Matekiluga*
30	*Fanoloa*

Sikaiana (an atoll in the Solomon Islands): After the Sikaiana dictionary of Donner (2012) (*74*), we gather only the following incomplete information about the local list:

- "The night of a full moon" is called *sseni ma tahi*; after this night, the nights of the moon are counted following a numerical sequence as *sseni ma lua*, *sseni ma tolu*, etc.
- From the 24th night onward the nights are counted as: *poo i ulu, poo i mata, poo i loto, poo i muli, poo i te mmana, tumaitu*. *Tumaitu*, the 29th (or 30th) night is the night of a new moon.

The list is therefore very similar to that of Takuu (see next).

Takuu (atoll off the east coast of Bougainville Island, Papua New Guinea): Reported in Molye (2011: 193, but see also 35, 78, 334) (*75*). The glosses suggest some uncertainty about the exact position of the new moon (28th or 29th night?). This may be linked to the issue that the list includes only 29 nights. The first nine nights form a series called *tuu*, glossed by Molye as "positioned within the lunar month" and "likely to be used if the speaker can see the moon, otherwise it is optional". This may be cognate with Proto-East-Polynesian **Tuu*, which names initial nights in East Polynesian lists (see S1A). It is unclear whether **Tuu* refers to a deity's name or the verb **tu(ʔ)u* '(to be) up', alluding to the rising moon (see S1C). The list also uses the particle *maa* as a conjunction in counting lunar phases of the moon (in addition to compound numbers): e.g. *tuu maa tasi* (lit. lunar cycle and one) "the first day of the lunar cycle". This is cognate with the **maa* conjunction found in East Polynesian forms such as **maseʔa/sameʔa-maa*-NUMBER (see form 'maa' in S1C). The 12th night includes the word *hakaoti* 'final(ize)', which has a counterpart in the East Polynesian lists, although this may be a trivial feature.

1	*tuu maa tasi*
2	*tuu maa rua*
3	*tuu maa toru*
4	*tuu maa haa*
5	*tuu maa rima*
6	*tuu maa ono*
7	*tuu maa hitu*
8	*tuu maa varu*
9	*tuu maa sivo*
10	*kaatoa*
11	(*Te marama raa ku*) *ttoe*
12	*suru hakaoti*
13	*hakatau tea*
14	*tamaa mua*
15	*hakatau nohoaki* (full moon)
16	*seni mua*
17	*seeni maa rua*
18	*seni maa toru*
19	*seni maa haa*
20	*seni maa rima*
21	*seni maa ono*
22	*seni maa hitu*
23	*seni maa varu*
24	*seni maa sivo*
25	(*ku*) *ttoe*
26	*suru hakaoti*
27	*hakatau tea*

28	*tuu maa itu* "(lit. spirits standing). Last night of the lunar cycle, night of the new moon, so-called because the moon is invisible to human eyes on this night."
29	(*Te marama raa ku*) *ara* "The moon is in the final night of its lunar cycle (i.e. it is a new moon)."

Tokelau, version 1: Collected in Macgregor (1937: 91–2) (*76*). Like the Samoan list, it primarily uses numerical counting expressions.

1	*Fakatasi*
2	*Fakalua*
3	*Fakatolu*
4	*Fakafa*
5	*Fakalima*
6	*Fakaono*
7	*Fakafitu*
8	*Fakavalu*
9	*Fakaiva*
10	*Mangafulu*
11	*Fakatasi*
12	*Fakalua*
13	*Utua* (“[The moon] is drawn up”)
14	*Malama* (Full Moon)
15	*Fakatasi*
16	*Fakalua*
17	*Fakatolu*
18	*Fakafa*
19	*Fakalima*
20	*Fakatutupu* (plural of “cause to grow”)
21	*Mangafulu*
22	*Po hiva*
23	*Po valu*
24	*Po fitu*
25	*Po ono*
26	*Po lima*
27	*Fanouluata* (“The [moon’s] head has perished in the shadow”)
28	*Fanolotoata* (“The [moon’s] heart has perished in the shadow”)
29	*Mate ki lunga* (“The [moon] dies above the horizon”)
30	*Fanoloa* (“[The night] of long death”)

Tokelau, version 2: Based on the entries of the Tokelau dictionary of Simona (1986) (*77*) and the list given in Hooper and Huntsman (1991: 165) (*78*).

1	*Fakatahi / Fakatahi o tua* (New Moon)
2	*Fakalua / Fakalua o tua*
3	*Fakatolu / Fakatolu o tua*
4	*Fakafaa / Fakafaa o tua*
5	*Fakalima / Fakalima o tua*
6	*Fakaona / Fakaona o tua*
7	*Fakafitu / Fakafitu o tua*
8	*Fakavalu / Fakavalu o loto*
9	*Fakaiva / Fakaiva o loto*
10	*Magafulu o loto*
11	*Fakatahi / Fakatahi o loto*
12	*Fakalua / Fakalua o loto*
13	*Utuaa*
14	*Maalama* (Full Moon)
15	*Fakatahi o namo / Ekematahi*
16	*Fakalua o namo*
17	*Fakatolu o namo*
18	*Fakafaa o namo*
19	*Fakalima o namo*
20	*Fakatutupu*
21	*Magafulu* (*o namo*) / *Poagafulu*
22	*Poo iva*
23	*Poo valu*
24	*Poo fitu*
25	*Poo ono*
26	*Poo lima*
27	*Fanouluata*
28	*Fanolotoata*
29	*Matekilunga*

Tonga: Collocott (1922: 15–16; 1925a,b) (*79–81*) published only partial information, which is completed and nuanced by the recent report of Perminow (2022: 178-9) (*82*). Differently from the list of Perminow, Collocott (1922: 170) also stated: "In counting the waning moons a nomenclature is employed similar to that used in reckoning the waxing moon, but in the reverse direction, *faka-valu*, eight-wise, *faka-fitu*, seven-wise, on down to *faka-ua*, two-wise which immediately precedes the night on which the moon is dead. This nomenclature seems to run parallel with the reckoning of the *ahoia* nights for a portion of the waning moon". One description of the first night is "First standing (*tu'u*) of the moon," which is reminiscent of East-Polynesian **Tuu*. The 19th and 20th nights feature the word *Matofi*, which, as noted by Collocott (1925a: 146) (*80*), has an East-Polynesian cognate. Overall, the list includes numerous alternative names, showing little standardization, and many of them are lengthy expressions.

	Reconstructed after Collocott (1922: 169-70; 1925a,b)	Perminow (2022: 178-9)
1	*Po ae faahikehe* ("Night of the spirit")	*Fua tu'u 'a e māhina* "First standing of the moon (or supporting the standing of the moon)" / *'Iloa 'e he fa'ahikehe* "Known by the other side/kind" / *Pō fa'ahikehe* "Night of the other side/kind"
2	*Po ae toutai* ("Sailor's night")	*Fakaua* "The second" / *'Iloa 'e he toutai* "Known by the fishermen/navigators" / *Pō toutai* "Night of the navigators/fishermen"
3	*Faka-ua* ("Two-wise")	*Fakatolu* "The third" / *'Iloa 'e he maama* "Known by the world/light" / *'Iloa 'e he tangata* "Known by man"
4	*Faka-tolu* (not given but inferrable)	*Fakafā* "The fourth"
5	*Faka-fa* (not given but inferrable)	*Fakanima* "The fifth"
6	*Faka-nima* (not given but inferrable)	*Fakaono* "The sixth"
7	*Faka-ono* (not given but inferrable)	*Fakafitu* "The seventh" / *Tu'u efiafi 'a e māhina* "The moon stands in the early evening"
8	*Faka-hitu*	*Fakavalu* "The eighth" / *Tu'u efiafi 'a e māhina* "The moon stands in the evening"
9	*Faka-valu / Bunifaga*	*Fakahiva* "The ninth"
10	*Fuofuavale* ("Incomplete")	*Fakahongofulu* "The tenth"
11	*Mahina katoa* ("All moon")	*Fua'aho* "Carrying/supporting day" / *'Aho punifanga* "The two sides facing one another"
12	*Fe-aho-aki* ("Caught between two days")	*Fuofua vale* "Incomplete/incompetent shape" / *Fakatauata* "Exposed to the twilight of dawn/moving toward dawn"
13	*Uluaki ahoia* (First "caught by daylight," "out all night")	*Fuofuanoa* "Unimportant shape/shape of no account" / *Māhina kātoa* "Whole/complete moon"
14	Second *ahoia* night / *Ma-tofi* (waning moon)	*Māhina kātoa* "Complete moon" / *Fē'ahoaki* "Reciprocal day" / *Fu'u maama lahi 'a e māhina* "The moon lights very much" / *'Uluaki māhina hopo* "First moonrise"
15	Third *ahoia* night	*Fakamāhina hopo* "Corresponding with moonrise"

16	(...)	*Fakamāhina hopo* “Corresponding with moonrise”
17	(...)	*Fakamāhina hopo* “Corresponding with moonrise”
18	(...)	*Kaupo'uli* “The dark ones”
19	(...)	*Kaupo'uli* “The dark ones”<br>/ *Matofi* “cut, hacked off/cuttings”
20	(...)	*Matofi tele* “Peeling the cuttings”
21	(...)	*'Aho ika* “Fish day”
22	(...)	*'Aho 'o e tafe* “Day of running water”<br>/ *Vaeua mālie 'a e māhina* “The moon is divided in two equal parts”<br>/ *Kalipa* “Fourth quarter”
23	(...)	*Kalipa* “V-shaped moon”
24	(...)	*Māhina vai* “Weak moon”
25	(...)	*Māhina vai* “Weak moon”
26	*Leke-leka-mate* (“almost dead?”)	*Fungaata* “The surface/top of the twilight of dawn”<br>/ *Lekeleka* “Small, low”
27	(...)	*Lekeleka mate* “Small, low and dying”
28	(...)	*Māhina 'asi vaivai* “A weakly appearing moon”
29	(...)	*Mate 'a e Māhina* “The moon is dead”

Vaitupu (Tuvalu): Reported in Kennedy (1929: 9) (*83*). As with other lists, counting expressions are prevalent. Apart from these trivial numerical forms, there are no names that compare to those in the East Polynesian lists.

1	*Sae ki tangata*—New moon. (*Sae*: appears—something that has been lost.)
2	*Ma lua*
3	*Ma tolu*
4	*Ma fa*
5	*Ma lima*
6	*Ma ono*
7	*Ma fitu*
8	*Ma valu*
9	*Ma hiva*
10	*Ma ngafulu*
11	*Fatatea*
12	*Te fou*
13	*Telamanganai* (Sunset and Moonrise) (*Se lama i te afiafi se lama i te vaveao*) (Impossible to go torch-fishing for flying-fish either in the evening or in the early morning)
14	*Te uluaki sengia* "1st night of disappearance (*Sengi*)"
15	*Sengia fakalua* "2nd night of disappearance"
16	*Sengia fakatolu* "3rd night of disappearance"
17	*Sengia fakafa* "4th night of disappearance"
18	*Sengia fakalima* "5th night of disappearance"
19	*Sengia fakaono* "6th night of disappearance"
20	*Sengia fakafitu* "7th night of disappearance"
21	*Sengia fakavalu* "8th night of disappearance"
22	*Sengia fakaiva* "9th night of disappearance"
23	*Sengia fakangafulu* "10th night of disappearance"
24	*Tuputasi* "1st day of moon in the morning sky"
25	*Tupulua* "2nd day of moon in the morning sky"
26	*Tuputolu* "3rd day of moon in the morning sky"
27	*Tokatulituli* "Sun following (*tuli*) the moon"
28	*Tuatua* "Sun and moon together"

S2 Dataset. (separate file: S2_ EPolyCal_dataset.csv)

Dataset (.csv spreadsheet) containing the 48 lunar calendrical lists of East Polynesia in normalized notation, as used for computational analysis.

References

(Used also in main text)

1. Kirch, P., Green, R., (2001). *Hawaiki*, *Ancestral Polynesia: An Essay in Historical Anthropology*. Cambridge: Cambridge University Press.
2. Wilmshurst, J. M., Hunt, T. L., Lipo, C. P. & Anderson, A. J. (2011). High-precision radiocarbon dating shows recent and rapid initial human colonization of East Polynesia. *Proceedings of the National Academy of Sciences* 108, 1815–1820, DOI: 10.1073/pnas.1015876108.
3. Ioannidis, A. G. et al. (2021). Paths and timings of the peopling of Polynesia inferred from genomic networks. *Nature* 597, 522–526, DOI: 10.1038/s41586-021-03902-8.
4. Wilson, W. H. (2021). East Polynesian Subgrouping and Homeland Implications Within the Northern Outlier–East Polynesian Hypothesis. *Oceanic Linguistics* 60(1), 36-71, DOI: 10.1353/ol.2021.0001
5. Pawley, A. (1966). Polynesian languages: a subgrouping based on shared innovations in morphology. *The Journal of the Polynesian Society* 75(1), 39–64.
6. Biggs, B. (1971), The Languages of Polynesia. In: T. A. Sebeok (ed.), *Linguistics in Oceania*. The Hague: Mouton De Gruyter.
7. Hiroa, T. R. [P. H. Buck] (1938). *Ethnology of Mangareva*. Bernice P. Bishop Museum Bulletin 157). Honolulu: Bernice P. Bishop Museum.
8. Burrows, E. G. (1938). *Western Polynesia, a Study in Cultural Differentiation*, Ethnological studies. Göteborg: Kaudern.
9. Horley, P. (2011). Lunar calendar in rongorongo texts and rock art of Easter Island. *Journal de la Société des Oceanistes* 132, 17-38.
10. Valério, M., Lastilla, L. & Ravanelli, R. (2022). The Rongorongo Tablet C: new technologies and conventional approaches to an undeciphered text. *Lingue e Linguaggio* 21, 333–367.
11. Valério, M. (2024). The Rongorongo 'Lunar Calendar' of Rapa Nui (Easter Island) and the type of script. In S. Ferrara, B. Montecchi, & M. Valério (Eds.), *Writing from invention to decipherment* (pp. 189–224). Oxford: Oxford University Press.
12. Stimson, J. F. (1928). Tahitian names for the nights of the moon. *The Journal of the Polynesian Society* 37, 326–337.
13. Best, E. (1922). *The Maori Division of Time*. Dominion Museum Monograph 4. Wellington: Dominion Museum.
14. Craig, R. D. (1989). *Dictionary of Polynesian Mythology*. New York: Greenwood Press.
15. Greenhill, S. J. & Clark, R. (2011). POLLEX-Online: The Polynesian Lexicon Project Online, *Oceanic Linguistics* 50(2): 551–559. Available from: https://pollex.eva.mpg.de/
16. Hiroa, T. R. [P. H. Buck] (1932). *Ethnology of Manihiki and Rakahanga*. Bernice P. Bishop Museum Bulletin 99. Honolulu: Bernice P. Bishop Museum.

17. Métraux, A. (1940). *Ethnology of Easter Island*. Bernice P. Bishop Museum Bulletin 160. Honolulu: Bernice P. Bishop Museum.
18. Green, R. (1966). Linguistic subgrouping within Polynesia: The implications for prehistoric settlement. *The Journal of the Polynesian Society* 75(1), 6–38.
19. Marck, J. (2000). *Topics in Polynesian Language and Culture History*. Canberra: Pacific Linguistics, Research School of Pacific and Asian Studies, The Australian National University.
20. Walworth, M. (2014). Eastern Polynesian: The Linguistic Evidence Revisited. *Oceanic Linguistics* 53(2), 256–272.
21. Stimson, J. F. (1930). A discussion of the hamzah and some allied aspects of Polynesian phonetics. *Journal of the Polynesian Society,* 39(3), 263–283.
22. Denoon, D. (2004). *The Cambridge History of the Pacific Islanders*. Cambridge: Cambridge University Press.
23. Hiroa, T. R. [P. H. Buck] (1932). *Ethnology of Tongareva*. Bernice P. Bishop Museum Bulletin 92. Honolulu: Bernice P. Bishop Museum.
24. Fisman, D., Grogin, J., Margalit, O. & Weiss, G. (2022). The Normalized Edit Distance with Uniform Operation Costs Is a Metric. In H. Bannai, J. Holub (Eds.), *33rd Annual Symposium on Combinatorial Pattern Matching, CPM 2022, June 27-29, 2022, Prague, Czech Republic*, (Schloss Dagstuhl - Leibniz-Zentrum für Informatik, 2022), vol. 223 of LIPIcs, pp. 17:1–17:17.
25. Li, Y. & Liu, B. (2007). A Normalized Levenshtein Distance Metric. *IEEE Transactions on Pattern Analysis and Machine Intelligence* 29(6), 1091–1095.
26. Bariffi, J., Bartz, H., Liva, G. & Rosenthal, J. (2022). On the Properties of Error Patterns in the Constant Lee Weight Channel. In A. Lapidoth & S. M. Moser (Eds.), *International Zurich Seminar on Information and Communication (IZS 2022). Proceedings*, 44–48. Zurich: ETH Zurich.
27. D. H. Huson, D. Bryant, The SplitsTree App: interactive analysis and visualization using phylogenetic trees and networks. *Nature Methods* 21, 1773–1774, DOI: 10.1038/s41592-024-02406-3 (2024).
28. Huson, D. H. & Bryant, D. (2024). The SplitsTree App: interactive analysis and visualization using phylogenetic trees and networks. *Nature Methods* 21, 1773–1774, DOI: 10.1038/s41592-024-02406-3.
29. Bryant, D. & Moulton, V. (2004). Neighbor-Net: An Agglomerative Method for the Construction of Phylogenetic Networks. *Molecular Biology and Evolution* 21(2), 255–265, DOI: 10.1093/molbev/msh018.
30. Holland, B. R., Huber, K. T., Dress, A. & Moulton, V. (2002). δ Plots: A Tool for Analyzing Phylogenetic Distance Data. *Molecular Biology and Evolution* 19(12), 2051–2059, DOI: 10.1093/oxfordjournals.molbev.a004030.
31. Gray, R. D., Bryant, D. & Greenhill, S. J. (2010). On the Shape and Fabric of Human History. *Philosophical Transactions of the Royal Society B: Biological Sciences* 365(1559), 3923–3933, DOI: 10.1098/rstb.2010.0162.
32. Saitou, N. & Nei, M. (1987). The Neighbor-joining Method: A New Method for Reconstructing Phylogenetic Trees. *Molecular Biology and Evolution* 4, 406–425, DOI: 10.1093/oxfordjournals.molbev.a040454
33. Tria, F. D. K., Landan, G. & Dagan, T. (2017). Phylogenetic Rooting Using Minimal Ancestor Deviation. *Nature Ecology & Evolution* 1, 0193, DOI: 10.1038/s41559-017-0193.

34. Waddell, P. J. & Azad, A. (2009). Resampling Residuals: Robust Estimators of Error and Fit for Evolutionary Trees and Phylogenomics. arXiv preprint arXiv:0912.5288. Available at: https://arxiv.org/abs/0912.5288.

(Only in this supporting document)

35. Edwards, A. (2015). *The orientation of sacred sites on Raivavae, The Austral Islands, French Polynesia (with a brief study of marae on Huahine & Raiatea)*. Flag #83 Expedition Report, May 2015. Technical Report. https://doi.org/10.13140/RG.2.2.34863.25762
36. Fornander, A. (1878). *The Polynesian race: Its origin and migrations, and the ancient history of the Hawaiian people to the times of Kamehameha I* (Vol. 1). London: Trübner.
37. Tregear, E. (1891). *Maori-Polynesian comparative dictionary*. Wellington: Lyon and Blair.
38. Andrews, L. (1836). *A vocabulary of words in the Hawaiian language*. Lahainaluna: Press of the High School.
39. Andrews, L. (1865). *A dictionary of the Hawaiian language: To which is appended an English-Hawaiian vocabulary and a chronological table of remarkable events*. Honolulu: H.M. Whitney.
40. Malo, D. (1951). *Hawaiian antiquities* (N. Emerson, Trans.). Honolulu: Bishop Museum Press. (Original work published 1838).
41. Langlas, C. M. (2017). Nā Pō o ka Malama: The "nights" of the Hawaiian month. *Palapala,* 1, 101–112.
42. Pukui, M. K., & Elbert, S. H. (1986). *Hawaiian dictionary: Hawaiian-English, English-Hawaiian* (Revised and enlarged ed.). Honolulu: University of Hawaii Press.
43. Andrews, L. and Parker, H. H. (1922). *A dictionary of the Hawaiian language.* Revised by Henry H. Parker. Honolulu: Board of Commissioners of Public Archives of the Territory of Hawaii.
44. Williams, H. W. (1928). The nights of the moon. *Journal of the Polynesian Society,* 37(3), 338–356.
45. Best, E. (1899). Notes on Maori mythology. *The Journal of the Polynesian Society,* 8(2), 93–121.
46. Williams, W. L. (1844). *A dictionary of the New Zealand language*. Paihia: N.Z.C.M. Society.
47. Fenton, F. D. (1885). *Suggestions for a history of the origin and migrations of the Maori people*. Auckland: H. Brett, Evening Star Office.
48. Firth, R. (1929). *Primitive economics of the New Zealand Maori*. London: Routledge.
49. Tregear, E. (1899). *Mangareva dictionary, Gambier islands*. Wellington: Government Printing Office.
50. Janeau, V.-F. (1908). *Essai de grammaire de la langue des îles Gambier ou Mangaréva*. Paris: Chadenat.
51. Shand, A. (1898). The Moriori people of the Chatham Islands: Their traditions and history. Chapter XIV – Tawhaki. *The Journal of the Polynesian Society,* 7(2), 73–88.
52. Tregear, E. (1889). The Moriori. *Transactions and Proceedings of the Royal Society of New Zealand,* 22, 75-79.
53. Audran, H. (1929). Étude comparative polynésienne concernant les noms des jours de lune. *Bulletin de la Société des Études Océaniennes,* 30, 260–261.
54. Handy, E. S. C. (1923). *The native culture in the Marquesas*. Honolulu: B. P. Bishop Museum.

55. Dordillon, R. I. (1931). *Grammaire et dictionnaire de la langue des Îles Marquises: Marquisien-Français*. Paris: Institut d'Ethnologie.
56. Shibata, N. (Ed.). (2003). *Penrhyn-English dictionary* (ELPR Publications Series A1-005). Kyoto: Nakanishi. https://penrhyn.cookislandsdictionary.com
57. Mokoroa, P. (1984). Maroro-Tu: The flyingfish return. In R. Crocombe (Ed.), *Atiu: An island community* (pp. 114-125). Suva: University of the South Pacific.
58. Gill, W. W. (1876). *Myths and songs from the South Pacific*. London: Henry S. King & Co.
59. Buse, J. (1996). *Cook Islands Maori dictionary with English-Cook Islands Maori finderlist*. Canberra: The Australian National University.
60. Thomson, W. J. (1891). Te Pito te Henua, or Easter Island. Report of the National Museum of Natural History for the year ending June 30, 1889. In *Annual reports of the Smithsonian Institution for 1889* (pp. 447–552). Washington, DC.
61. Englert, S. (1974). *La tierra de Hotu Matu'a: Historia, etnología, y lengua de la Isla de Pascua* (2nd ed.). Santiago de Chile: Universidad de Chile. (Original work published 1948).
62. Kondratov, A. M. (1965). The hieroglyphic signs and different lists in the manuscripts from Easter Island. In T. Heyerdahl & E. N. Ferdon Jr. (Eds.), *Reports of the Norwegian Archaeological Expedition to Easter Island and East Pacific* (Vol. 2, pp. 403–416). London: G. Allen & Unwin.
63. Ellis, W. (1829). *Polynesian researches, during residence of nearly six years in the South Sea Islands* (Vol. II). London: Fisher, Son, & Jackson.
64. Stimson, J. F. (1964). *A dictionary of some Tuamotuan dialects of the Polynesian language*. The Hague: Martinus Nijhoff.
65. Audran, H. (1919). Étude linguistique du dialecte particulier de Napuka. *Bulletin de la Société des Études Océaniennes,* 5, 36–41.
66. Danielsson, B. (1956). *Work and life on Raroia: An acculturation study from the Tuamotu group, French Oceania*. London: G. Allen & Unwin.
67. Horley, P., Davletshin, A., & Wieczorek, R. (2018). Rongo-Rongo: How many scripts were there on Easter Island? In Z. Jakubowska-Vorbrich (Ed.), *The sleep of reason produces monsters: Misconceptions about Easter Island in light of 21st century science* (pp. 323–468). Warsaw: Museum of the History of the Polish Popular Movement.
68. Elbert, S. H., Monberg, T. (1965). *Language and Culture of Rennell and Bellona Islands*. Honolulu: University of Hawaii Press.
69. Elbert, S. H. (1975). *Dictionary of the Language of Rennell and Bellona . Part 1 , Rennellese and Bellonese to English*. Copenhagen: Gyldendal National Museum of Denmark.
70. Ranby, P. (1980). *A Nanumea Lexicon*. Canberra: The Australian National University.
71. Sperlich , W. B. (ed.). (1997). *Tohi Vagahau Niue : Niue Language Dictionary* . Niue / Honolulu: Government of Niue / University of Hawai'i Press.
72. Beaglehole, E. and Beaglehole, P. (1938). *Ethnology of Pukapuka.* Honolulu: The Museum.
73. Anonymous (1928). The Samoan division of time. *Journal of the Polynesian Society*, 37(2), 228–240.
74. Donner, W. W. (2012). *Sikaiana Dictionary*. Available online at: https://research.library.kutztown.edu/sikaiana_dictionary/ (last access 29 September 2024).
75. Moyle, R. M. (2011). *Takuu grammar and dictionary: A Polynesian language of the South Pacific*. Canberra: Pacific Linguistics.
76. Macgregor, G. (1937). *Ethnology of Tokelau Islands*. Honolulu: Bishop Museum Press.

77. Simona, R. (1986). *Tokelau Dictionary*. New Zealand: Office of Tokelau Affairs.
78. Hooper, A. and Huntsman, J. (trans.) (1991). *Matagi Tokelau: History and Traditions of Tokelau*. Apia and Suva: Office of Tokelau Affairs and Institute of Pacific Studies, University of the South Pacific.
79. Collocott, E. E. V. (1922). *Tongan astronomy and calendar*. Honolulu: Bishop Museum Press.
80. Collocott, E. E. V. (1925a). Supplementary Tongan vocabulary. *Journal of the Polynesian Society*, 34(2), 146–169.
81. Collocott, E. E. V. (1925b). Supplementary Tongan vocabulary; also notes on measuring and counting, proverbial expressions, and phases of the moon (Continued). *Journal of the Polynesian Society*, 34(3), 193–213.
82. Perminow, A. A. (2022). *Engaging environments in Tonga: Cultivating beauty and nurturing relations in a changing world*. New York & Oxford: Berghahn Books.
83. Kennedy, D. G. (1929). Field notes on the culture of Vaitupu, Ellice Islands [Instalment No. 1, Memoir No. 9]. *Journal of the Polynesian Society*, 38(2), 1–7, 9–38.
